# Synthesis, *in vitro* enzyme activity and molecular docking studies of new benzylamine-sulfonamide derivatives as selective MAO-B inhibitors

**DOI:** 10.1080/14756366.2020.1784892

**Published:** 2020-06-30

**Authors:** Begüm Nurpelin Sağlık, Derya Osmaniye, Ulviye Acar Çevik, Serkan Levent, Betül Kaya Çavuşoğlu, Özlem Atlı Eklioğlu, Yusuf Özkay, Ali Savaş Koparal, Zafer Asım Kaplancıklı

**Affiliations:** a Department of Pharmaceutical Chemistry, Faculty of Pharmacy, Anadolu University, Eskişehir, Turkey; b Doping and Narcotic Compounds Analysis Laboratory, Faculty of Pharmacy, Anadolu University, Eskişehir, Turkey; c Department of Pharmaceutical Chemistry, Faculty of Pharmacy, Zonguldak Bülent Ecevit University, Zonguldak, Turkey; d Department of Pharmaceutical Toxicology, Faculty of Pharmacy, Anadolu University, Eskişehir, Turkey; e Open Education Faculty, Anadolu University, Eskişehir, Turkey

**Keywords:** Benzylamine, enzyme inhibition, heterocyclic ring, MAO enzymes, molecular docking

## Abstract

Many studies have been conducted on the selective inhibition of human monoamine oxidase B (*h*MAO-B) enzyme using benzylamine-sulphonamide derivatives. Using various chemical modifications on **BB-4h**, which was reported previously by our team and showed a significant level of MAO-B inhibition, novel benzylamine-sulphonamide derivatives were designed, synthesised, and their MAO inhibition potentials were evaluated. Among the tested derivatives, compounds **4i** and **4t** achieved IC_50_ values of 0.041 ± 0.001 µM and 0.065 ± 0.002 µM, respectively. The mechanism of *h*MAO-B inhibition by compounds **4i** and **4t** was studied using Lineweaver–Burk plot. The nature of inhibition was also determined to be non-competitive. Cytotoxicity tests were conducted and compounds **4i** and **4t** were found to be non-toxic. Molecular docking studies were also carried out for compound **4i**, which was found as the most potent agent, within *h*MAO-B catalytic site.

## 1. Introduction

Monoamine oxidase (MAO) is the enzyme responsible for catalysing the oxidative deamination of intracellular amines and monoamine neurotransmitters, which contributes to the regulation of the concentrations of these chemicals in the brain and in peripheral tissues^1^^,^^2^. MAOs, which are flavin adenine dinucleotide (FAD)-containing enzymes, are localised in the outer mitochondrial membranes of glial, neuronal, and other types of cells; they are particularly abundant in the liver and the brain. MAOs have two different isoforms, MAO-A and MAO-B, with 70% homology. The genes that code for the two isoforms are linked in opposite orientation on the X chromosome, differ in the specificity of their substrates and the selectivity of their inhibitors^3^. For example, MAO-B is selectively inhibited by selegiline, and utilises phenylethylamine and benzylamine as substrates. On the contrary, MAO-A is selectively inhibited by clorgiline, and utilises adrenaline, noradrenaline and serotonin as substrates. However, both isoforms may also act on the same substrates such as dopamine and tyramine^4^.

MAOs are of extensive pharmacological importance due to their roles in the metabolism of certain neurotransmitters. Selective MAO-A inhibitors are used clinically as antidepressants and anxiolytics, while MAO-B inhibitors are used to reduce the progression of Parkinson’s disease, and manage symptoms related to Alzheimer’s disease^5^. Moreover, MAO-catalyzed deamination reactions produce hydrogen peroxide as a byproduct, which may typically contribute to the oxidative stress state. In this context, MAO inhibitors are thought to act as neuroprotective agents in degenerative processes^6^^,^^7^.

Parkinson's disease (PD), which affects more than 5 million people worldwide, is the second most common disease after Alzheimer's disease. Considering the loss of nigrostriatal dopaminergic cells as a pathological hallmark of PD, therapeutic strategies have been established to boost the levels of dopamine in the brain^8–10^. Although dopamine is metabolised by both MAO isoforms, MAO-B is the more common isoform present in the basal ganglia and is therefore responsible for dopamine metabolism in this region^11^.

Currently, the Protein Data Bank contains more than 40 crystal structures of MAO (most of them MAO-B) in complex with different reversible and irreversible inhibitors, as observed through X-ray diffraction at refinements of 3.0–1.7 Å. Additionally, MAO-A shows a markedly different monopartite cavity (∼550 Å) compared to the bipartite cavity (290 Å) found in MAO-B. The “aromatic cage”—a hydrophobic binding pocket containing the FAD cofactor—is considered the active region^4^^,^^7^. The FAD is covalently attached to the cysteine residue of the protein, and the 8α-thioether linkage provides this connection. It is believed that the catalytic activity of the two tyrosine residues, Tyr398 and Tyr435, found in the *h*MAO-B structure is due to the polarisation of the amine N pair of the substrate^12^. Therefore, in designing a new inhibitor compound, it is desirable to have the amine group in the structure.

**Figure 1.**
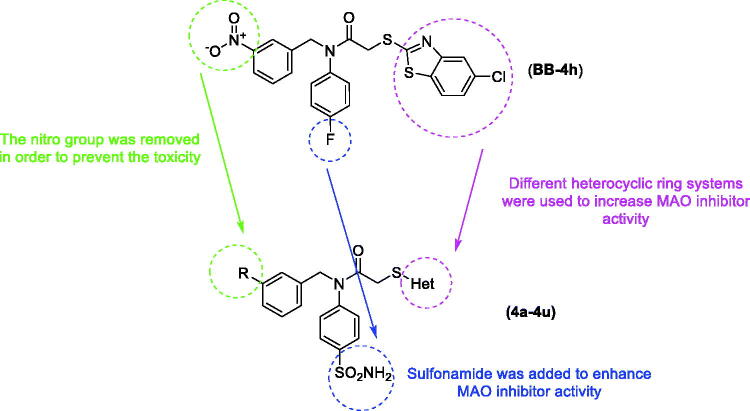
Design of the target compounds from compound **BB-4h**.

In light of the above-mentioned information, this study was conducted to develop new and potent MAO inhibitors. It has been thought that the proven MAO inhibition of benzylamine derivatives may provide MAO-B inhibitory activity due to strong interactions on the enzyme active side^13–15^. In our recent study^2^, we reported a new benzothiazole-benzylamine hybrid compound, 2-((5-chlorobenzothiazol-2-yl)thio)-*N*-(4-fluorophenyl)-*N*-(3-nitrobenzyl)acetamide (**BB-4h**), as shown in Figure 1, with significant IC_50_ (2.95 ± 0.09 µM) against MAO-B. Moreover, sulphonamides and various heterocyclic ring systems have been identified as inhibitors of MAO in previous studies^3^^,^^16–19^. Therefore, we considered the compound (**BB-4h**) as a lead compound, and we performed some modifications, such as removing nitro and fluoro groups, introducing a sulphonamide group, and changing heterocyclic rings in order to improve biological activity. Subsequently, 20 benzylamine derivatives containing a sulphonamide moiety and different heterocyclic ring moieties were synthesised, and their MAO inhibitory activities were evaluated in this study.

## 2. Experimental

### 2.1. Chemistry

All chemicals used in the synthesis studies were obtained from Merck Chemicals (Merck KGaA, Darmstadt, Germany) or Sigma-Aldrich Chemicals (Sigma-Aldrich Corp., St. Louis, MO). MP90 digital melting point apparatus (Mettler Toledo, Ohio) was used to determine the melting points of the resulting compounds and was presented uncorrected. A Bruker 300 MHz and 75 MHz digital FT-NMR spectrometer (Bruker Bioscience, Billerica, MA) in DMSO-*d_6_*, respectively recorded ^1^H NMR and ^13 ^C NMR spectra. In the NMR spectra, splitting patterns were determined recognised as follows: s: singlet; d: doublet; t: triplet; dd: double doublet; td: triple doublet; br.s.: bronsted singlet; m: multiplet. Coupling constants (*J*) are reported in units of Hertz (Hz). IRAffinity-1S Fourier transform IR (FTIR) spectrometer (Shimadzu, Tokyo, Japan) was used to record the IR spectra of the compounds. Mass spectra were recorded on an LCMS-IT-TOF (Shimadzu, Kyoto, Japan) by means of ESI method. Silica gel 60 F254 by TLC (Merck KGaA, Darmstadt, Germany) was used to control the purity of the obtained compounds.

#### 2.1.1. General procedure for the synthesis of the compounds

##### 2.1.1.1. Synthesis of 4-(benzylideneamino)benzenesulfonamide and 4-((4-methylbenzylidene)amino)benzenesulfonamide (**1a**, **1b**)

4-Aminobenzenesulfonamide (2.408 g, 0.014 mol) and benzaldehyde (1.427 ml, 0.014 mol) or 4-methylbenzaldehyde (1.680 g, 0.014 mol) were refluxed in EtOH (50 ml) for 48 h. Acetic acid was used as catalyst in this reaction. After completion of the reaction, the mixture was cooled, precipitated product was filtered and dried.

##### 2.1.1.2. Synthesis of 4-(benzylamino)benzenesulfonamide and 4-((4-methylbenzyl)amino)benzenesulfonamide (**2a**, **2b**)

4-(Benzylideneamino)benzenesulfonamide (**1a**) (3.328 g, 0.0128 mol) or 4-((4-methylbenzylidene)amino)benzenesulfonamide (**1b**) (3.507 g, 0.0128 mol) was dissolved in MeOH. Sodium borohydride was added to the reaction medium in portions of 0.5 moles. It was observed by controlling the end of the reaction with TLC that the reaction was complete when the total amount of sodium borohydride reached 1.5 moles. After completion of the reaction, the MeOH was removed by a rotavapor. The precipitated product was washed with deionised water to remove the excess of the sodium borohydride, dried, and recrystallized from EtOH.

##### 2.1.1.3. Synthesis of *N*-Benzyl-2-chloro-*N*-(4-sulfamoylphenyl)acetamide and 2-chloro-*N*-(4-methylbenzyl)-*N*-(4-sulfamoylphenyl)acetamide (**3a**, **3b**)

4-(Benzylamino)benzenesulfonamide (**2a**) (3.013 g, 0.0115 mol) or 4-((4-methylbenzyl)amino)benzenesulfonamide (**2b**) (3.174 g, 0.0115 mol) and triethylamine (TEA) (1.605 ml, 0.0115 mol) were dissolved in DMF (20 ml) and the reaction mixture was taken up in ice bath. The solution of chloroacetyl chloride (1.004 ml, 0.0126 mol) in DMF (10 ml) was added dropwise to the reaction mixture. After completion of the reaction, the mixture was poured into ice-water (50 ml), precipitated product was filtered, washed with deionised water, dried and recrystallized from EtOH.

##### 2.1.1.4. General procedure for the synthesis of target compounds (**4a**–**4u**)

*N*-Benzyl-2-chloro-*N*-(4-sulfamoylphenyl)acetamide (**3a**) (0.305 g, 0.0009 mol) or 2-chloro-*N*-(4-methylbenzyl)-*N*-(4-sulfamoylphenyl)acetamide (**3b**) (0.317 g, 0.0009 mol), heterocyclic substituted thiol derivatives (0.0009 mol) and sufficient quantity of potassium carbonate (K_2_CO_3_) (0.193 g, 0.0014 mol) were reacted in acetone for 3 h. After completion of the reaction, acetone was removed under reduced pressure, the residue was washed with water, dried, and recrystallized from EtOH.

###### *N*-Benzyl-2-((1-methyl-1*H*-imidazol-2-yl)thio)-*N*-(4-sulfamoylphenyl)acetamide (**4a**)

Yield: 85%, M.P. = 137–139 °C, FTIR (ATR, cm^−1^): 3340 (N–H), 2941 (C–H), 1666 (C = O), 709, 850. ^1^H-NMR (300 MHz, DMSO-*d_6_*): δ = 3.55 (3H, s, –CH_3_), 3.81 (2H, s, –CH_2_–), 4.92 (2H, s, -CH_2_), 6.90 (1H, s, Imidazole CH), 7.17–7.31 (6H, m, Imidazole CH, Monosubstitutedbenzene), 7.39 (2H, d, *J =* 8.3 Hz, 1,4-Disubstitutedbenzene), 7.78 (2H, d, *J =* 8.3 Hz, 1,4-disubstitutedbenzene). ^13 ^C-NMR (75 MHz, DMSO-*d_6_*): δ = 33.4, 37.9, 52.8, 123.8, 127.3, 127.7, 128.2, 128.8, 128.9, 129.1, 137.2, 139.7, 144.2, 144.5, 167.6. HRMS (m/z): [M + H]^+^ calcd for C_19_H_20_N_4_O_3_S_2_: 417.1055; found: 417.1040

###### *N*-Benzyl-2-((4-methyl-4*H*-1,2,4-triazol-3-yl)thio)-*N*-(4-sulfamoylphenyl)acetamide (**4b**)

Yield: 88%, M.P. = 201–203 °C, FTIR (ATR, cm^−1^): 3296 (N-H), 2976 (C–H), 1654 (C = O), 709, 852. ^1^H-NMR (300 MHz, DMSO-*d_6_*): δ = 3.55 (3H, s, –CH_3_), 4.01 (2H, s, –CH_2_–), 4.94 (2H, s, –CH_2_–), 7.18–7.31 (5H, m, Monosubstitutedbenzene), 7.42 (2H, s, –SO_2_NH_2_), 7.49 (2H, d, *J =* 8.5 Hz, 1,4-Disubstitutedbenzene), 7.81 (2H, d, *J =* 8.5 Hz, 1,4-Disubstitutedbenzene), 8.50 (1H, s, Triazole CH). ^13 ^C-NMR (75 MHz, DMSO-*d_6_*): δ = 31.2, 37.8, 52.9, 127.4, 127.8, 128.3, 128.9, 129.1, 137.1, 143.8, 144.5, 146.5, 149.1, 167.0. HRMS (m/z): [M + H]^+^ calcd for C_18_H_19_N_5_O_3_S_2_: 418.1002; found: 418.1001

###### *N*-Benzyl-2-((5-methyl-1,3,4-thiadiazol-2-yl)thio)-*N*-(4-sulfamoylphenyl)acetamide (**4c**)

Yield: 82%, M.P. = 164–166 °C, FTIR (ATR, cm^−1^): 3334 (N-H), 3047 (C-H), 1654 (C = O), 698, 740, 856. ^1^H-NMR (300 MHz, DMSO-*d_6_*): δ = 2.66 (3H, s, –CH_3_), 4.18 (2H, s, –CH_2_–), 4.97 (2H, s, –CH_2_–), 7.21–7.32 (5H, m, Monosubstitutedbenzene), 7.42 (2H, s, –SO_2_NH_2_), 7.53 (2H, d, *J =* 8.5 Hz, 1,4-Disubstitutedbenzene), 7.83 (2H, d, *J =* 8.5 Hz, 1,4-Disubstitutedbenzene). ^13 ^C-NMR (75 MHz, DMSO-*d_6_*): δ = 15.6, 38.3, 53.0, 127.4, 127.8, 128.3, 128.9, 129.1, 137.1, 143.9, 144.5, 164.6, 166.0, 166.6. HRMS (m/z): [M + H]^+^ calcd for C_18_H_18_N_4_O_3_S_3_: 435.0614; found: 435.0622

###### *N*-Benzyl-2-((1-methyl-1*H*-tetrazol-5-yl)thio)-*N*-(4-sulfamoylphenyl)acetamide (**4d**)

Yield: 86%, M.P. = 124–127 °C, FTIR (ATR, cm^−1^): 3305 (N-H), 2931 (C–H), 1651 (C = O), 702, 734, 848. ^1^H-NMR (300 MHz, DMSO-*d_6_*): δ = 3.95 (3H, s, –CH_3_), 4.19 (2H, s, –CH_2_–), 4.96 (2H, s, –CH_2_–), 7.20–7.32 (7H, m, Monosubstitutedbenzene, –SO_2_NH_2_), 7.51 (2H, d, *J =* 8.4 Hz, 1,4-Disubstitutedbenzene), 7.84 (2H, d, *J =* 8.4 Hz, 1,4-Disubstitutedbenzene). ^13 ^C-NMR (75 MHz, DMSO-*d_6_*): δ = 34.1, 38.3, 53.0, 127.5, 127.8, 128.3, 128.9, 129.0, 137.0, 144.1, 144.6, 153.8, 166.5. HRMS (m/z): [M + H]^+^ calcd for C_17_H_18_N_6_O_3_S_2_: 419.0955; found: 419.0956

###### *N*-Benzyl-2-((1-phenyl-1*H*-tetrazol-5-yl)thio)-*N*-(4-sulfamoylphenyl)acetamide (**4e**)

Yield: 81%, M.P. = 138–140 °C, FTIR (ATR, cm^−1^): 3356 (N-H), 2949 (C–H), 1651 (C = O), 734, 759, 848. ^1^H-NMR (300 MHz, DMSO-*d_6_*): δ = 4.27 (2H, s, –CH_2_–), 4.95 (2H, s, –CH_2_–), 7.21–7.32 (5H, m, Monosubstitutedbenzene), 7.51 (2H, d, *J =* 8.4 Hz, 1,4-Disubstitutedbenzene), 7.67 (7H, br.s., Monosubstitutedbenzene, -SO_2_NH_2_), 7.84 (2H, d, *J =* 8.4 Hz, 1,4-Disubstitutedbenzene). ^13 ^C-NMR (75 MHz, DMSO-*d_6_*): δ = 38.5, 53.0, 124.9, 124.9, 127.5, 127.8, 128.3, 128.9, 130.4, 130.6, 130.7, 131.2, 133.5, 137.0, 154.3, 166.3. HRMS (m/z): [M + H]^+^ calcd for C_22_H_20_N_6_O_3_S_2_: 481.1111; found: 481.1096

###### *N*-Benzyl-2-(pyridin-2-ylthio)-*N*-(4-sulfamoylphenyl)acetamide (**4f**)

Yield: 87%, M.P. = 106–108 °C, FTIR (ATR, cm^−1^): 3402 (N-H), 2929 (C-H), 1651 (C = O), 698, 763, 854. ^1^H-NMR (300 MHz, DMSO-*d_6_*): δ = 3.94 (2H, s, –CH_2_–), 4.96 (2H, s, –CH_2_–), 7.11 (1H, t, *J =* 5.9 Hz, Pyridine CH), 7.21–7.31 (6H, m, Monosubstitutedbenzene, Pyridine CH), 7.41 (2H, s, –SO_2_NH_2_), 7.52 (2H, d, *J =* 8.4 Hz, 1,4-Disubstitutedbenzene), 7.62 (1H, t, *J =* 8.5, Pyridine CH), 7.83 (2H, d, *J =* 8.4 Hz, 1,4-Disubstitutedbenzene), 8.36 (1H, d, *J =* 4.3 Hz, Pyridine CH). ^13 ^C-NMR (75 MHz, DMSO-*d_6_*): δ = 33.4, 52.9, 120.4, 122.0, 127.4, 127.7, 128.2, 128.9, 129.1, 137.1, 137.5, 143.6, 145.2, 149.7, 157.5, 168.1. HRMS (m/z): [M + H]^+^ calcd for C_20_H_19_N_3_O_3_S_2_: 414.0941; found: 414.0950

###### 2-(Benzoxazol-2-ylthio)-*N*-benzyl-*N*-(4-sulfamoylphenyl)acetamide (**4g**)

Yield: 80%, M.P. = 82–84 °C, FTIR (ATR, cm^−1^): 3363 (N-H), 2985 (C-H), 1651 (C = O), 704, 744, 846. ^1^H-NMR (300 MHz, DMSO-*d_6_*): δ = 4.25 (2H, s, –CH_2_–), 4.99 (2H, s, –CH_2_–), 7.26–7.29 (5H, m, Monosubstitutedbenzene), 7.31–7.35 (2H, m, Benzoxazole CH), 7.45 (2H, s, –SO_2_NH_2_), 7.58–7.64 (4H, m, 1,4-Disubstitutedbenzene, Benzoxazole CH), 7.88 (2H, d, *J =* 8.4, 1,4-Disubstitutedbenzene), ^13 ^C-NMR (75 MHz, DMSO-*d_6_*): δ = 36.9, 53.2, 110.7, 118.7, 124.8, 125.1, 127.6, 127.8, 128.2, 128.9, 129.2, 137.1, 141.6, 144.0, 144.6, 151.7, 164.3, 166.6. HRMS (m/z): [M + H]^+^ calcd for C_22_H_19_N_3_O_4_S_2_: 454.0890; found: 454.0880

###### 2-(Benzothiazol-2-ylthio)-*N*-benzyl-*N*-(4-sulfamoylphenyl)acetamide (**4h**)

Yield: 79%, M.P. = 88–90 °C, FTIR (ATR, cm^−1^): 3352 (N–H), 2941 (C–H), 1651 (C = O), 702, 758, 846. ^1^H-NMR (300 MHz, DMSO-*d_6_*): δ = 4.24 (2H, s, –CH_2_–), 4.99 (2H, s, –CH_2_–), 7.24 (5H, br.s., Monosubstitutedbenzene), 7.37 (1H, td, *J_1_*=1.0 Hz, *J_2_*=7.7 Hz, Benzothiazole CH) 7.44 (2H, s, -SO_2_NH_2_), 7.49 (1H, td, *J_1_*=1.1 Hz, *J_2_*=7.7 Hz, Benzothiazole CH), 7.62 (2H, d, *J =* 8.3 Hz, 1,4-Disubstitutedbenzene), 7.83 (1H, d, *J =* 8.0, Benzothiazole CH), 7.88 (2H, d, *J =* 8.4 Hz, 1,4-Disubstitutedbenzene), 8.01 (1H, d, *J =* 7.6 Hz, Benzothiazole CH). ^13 ^C-NMR (75 MHz, DMSO-*d_6_*): δ = 37.2, 53.1, 121.6, 122.4, 125.0, 126.8, 127.5, 127.8, 128.3, 128.9, 129.1, 135.2, 137.2, 143.8, 144.8, 152.9, 166.3, 166.8. HRMS (m/z): [M + H]^+^ calcd for C_22_H_19_N_3_O_3_S_3_: 470.0661; found: 470.0652

###### *N*-Benzyl-2-((5-chlorobenzothiazol-2-yl)thio)-*N*-(4-sulfamoylphenyl)acetamide (**4i**)

Yield: 85%, M.P. = 114–116 °C, FTIR (ATR, cm^−1^): 3473 (N-H), 2995 (C–H), 1645 (C = O), 702, 732, 846. ^1^H-NMR (300 MHz, DMSO-*d_6_*): δ = 4.22 (2H, s, –CH_2_–), 4.98 (2H, s, –CH_2_–), 7.24 (5H, br.s., Monosubstitutedbenzene), 7.41 (1H, d, *J* = 1.8 Hz, Benzothiazole CH) 7.44 (2H, s, –SO_2_NH_2_), 7.62 (2H, d, *J* = 8.3 Hz, 1,4-Disubstitutedbenzene), 7.88–7.90 (3H, m, Benzothiazole CH, 1,4-Disubstitutedbenzene), 8.04 (1H, d, *J =* 8.6 Hz, Benzothiazole CH). ^13 ^C-NMR (75 MHz, DMSO-*d_6_*): δ = 37.4, 53.1, 121.0, 123.8, 125.0, 127.6, 127.8, 128.3, 128.8, 129.2, 131.7, 134.1, 137.3, 143.9, 144.7, 153.7, 166.6, 169.2. HRMS (m/z): [M + H]^+^ calcd for C_22_H_18_ ClN_3_O_3_S_3_: 504.0272; found: 504.0250

###### *N*-Benzyl-2-((5-methoxybenzothiazol-2-yl)thio)-*N*-(4-sulfamoylphenyl)acetamide (**4j**)

Yield: 80%, M.P. = 170–172 °C, FTIR (ATR, cm^−1^): 3483 (N-H), 2939 (C-H), 1651 (C = O), 702, 734, 842. ^1^H-NMR (300 MHz, DMSO-*d_6_*): δ = 3.84 (3H, s, –OCH_3_), 4.23 (2H, s, –CH_2_–), 4.99 (2H, s, –CH_2_–), 7.01 (1H, dd, *J_1_*=2.5 Hz, *J_2_*=8.8 Hz, Benzothiazole CH), 7.25 (5H, br.s., Monosubstitutedbenzene), 7.33 (1H, d, *J* = 2.5 Hz, Benzothiazole CH) 7.43 (2H, br.s, -SO_2_NH_2_), 7.59 (2H, d, *J* = 8.4 Hz, 1,4-Disubstitutedbenzene), 7.86 (1H, d, *J =* 8.7, Benzothiazole CH), 7.88 (2H, d, *J =* 8.2 Hz, 1,4-Disubstitutedbenzene). ^13 ^C-NMR (75 MHz, DMSO-*d_6_*): δ = 37.3, 53.1, 56.0, 105.0, 114.2, 122.6, 126.8, 127.5, 127.8, 128.2, 128.9, 129.1, 137.2, 143.9, 144.7, 154.2, 159.2, 166.8, 167.2. HRMS (m/z): [M + H]^+^ calcd for C_23_H_21_N_3_O_4_S_3_: 500.0767; found: 500.0761

###### 2-((1-Methyl-1*H*-imidazol-2-yl)thio)-*N*-(4-methylbenzyl)-*N*-(4-sulfamoylphenyl)acetamide (**4k**)

Yield: 86%, M.P. = 153–155 °C, FTIR (ATR, cm^−1^): 3313 (N–H), 2920 (C–H), 1651 (C = O), 840. ^1^H-NMR (300 MHz, DMSO-*d_6_*): δ = 2.25 (3H, s, –CH_3_), 3.56 (3H, s, –CH_3_), 3.80 (2H, s, –CH_2_–), 4.87 (2H, s, –CH_2_–), 6.90 (1H, d, *J =* 1.1 Hz, Imidazole CH), 7.04–7.10 (4H, m, Methylbenzene), 7.21 (1H, d, *J =* 1.1 Hz, Imidazole CH), 7.37–7.41 (4H, m, –SO_2_NH_2_, 1,4-Disubstitutedbenzene), 7.79 (2H, d, *J =* 8.5 Hz, 1,4-Disubstitutedbenzene). ^13 ^C-NMR (75 MHz, DMSO-*d_6_*): δ = 21.1, 33.4, 37.9, 52.5, 123.8, 127.3, 128.3, 128.9, 129.0, 129.44, 134.1, 136.9, 140.0, 143.6, 144.7, 167.5. HRMS (m/z): [M + H]^+^ calcd for C_20_H_22_N_4_O_3_S_2_: 431.1206; found: 431.1199

###### 2-((4-Methyl-4*H*-1,2,4-triazol-3-yl)thio)-*N*-(4-methylbenzyl)-*N*-(4-sulfamoylphenyl)acetamide (**4l**)

Yield: 84%, M.P. = 229–231 °C, FTIR (ATR, cm^−1^): 3302 (N-H), 3049 (C–H), 1654 (C = O), 852. ^1^H-NMR (300 MHz, DMSO-*d_6_*): δ = 2.25 (3H, s, -CH_3_), 3.55 (3H, s, –CH_3_), 4.00 (2H, s, –CH_2_–), 4.90 (2H, s, -CH_2_-), 7.08 (4H, br.s., Methylbenzene), 7.35 (2H, br.s, -SO_2_NH_2_), 7.46 (2H, d, *J =* 8.5 Hz, 1,4-Disubstitutedbenzene), 7.81 (2H, d, *J =* 8.5 Hz, 1,4-Disubstitutedbenzene), 8.51 (1H, s, Triazole CH). ^13 ^C-NMR (75 MHz, DMSO-*d_6_*): δ = 21.1, 31.2, 37.9, 52.6, 127.4, 128.3, 129.0, 129.5, 134.0, 136.9, 144.1, 144.4, 146.5, 149.1, 167.0. HRMS (m/z): [M + H]^+^ calcd for C_19_H_21_N_5_O_3_S_2_: 432.1159; found: 432.1161

###### 2-((5-Methyl-1,3,4-thiadiazol-2-yl)thio)-*N*-(4-methylbenzyl)-*N*-(4-sulfamoylphenyl)acetamide (**4m**)

Yield: 79%, M.P. = 178–180 °C, FTIR (ATR, cm^−1^): 3288 (N-H), 3070 (C-H), 1651 (C = O), 850. ^1^H-NMR (300 MHz, DMSO-*d_6_*): δ = 2.25 (3H, s, –CH_3_), 2.66 (3H, s, –CH_3_), 4.16 (2H, s, –CH_2_–), 4.92 (2H, s, –CH_2_–), 7.09 (4H, br.s., Methylbenzene), 7.43 (2H, s, –SO_2_NH_2_), 7.51 (2H, d, *J =* 8.5 Hz, 1,4-Disubstitutedbenzene), 7.83 (2H, d, *J =* 8.5 Hz, 1,4-Disubstitutedbenzene). ^13 ^C-NMR (75 MHz, DMSO-*d_6_*): δ = 15.6, 21.1, 38.3, 52.7, 127.4, 128.3, 129.1, 129.5, 134.0, 136.9, 143.9, 144.5, 164.6, 166.0, 166.6. HRMS (m/z): [M + H]^+^ calcd for C_19_H_20_N_4_O_3_S_3_: 449.0770; found: 449.0750

###### 2-((1-Methyl-1*H*-tetrazol-5-yl)thio)-*N*-(4-methylbenzyl)-*N*-(4-sulfamoylphenyl)acetamide (**4n**)

Yield: 80%, M.P. = 154–156 °C, FTIR (ATR, cm^−1^): 3338 (N-H), 2947 (C–H), 1649 (C = O), 850. ^1^H-NMR (300 MHz, DMSO-*d_6_*): δ = 2.25 (3H, s, –CH_3_), 3.95 (3H, s, –CH_3_), 4.19 (2H, s, –CH_2_–), 4.91 (2H, s, –CH_2_–), 7.09 (4H, br.s., Methylbenzene), 7.33 (2H, br.s, –SO_2_NH_2_), 7.50 (2H, d, *J =* 8.4 Hz, 1,4-Disubstitutedbenzene), 7.84 (2H, d, *J =* 8.4 Hz, 1,4-Disubstitutedbenzene). ^13 ^C-NMR (75 MHz, DMSO-*d_6_*): δ = 21.1, 34.1, 38.4, 52.7, 127.5, 128.3, 129.1, 129.5, 133.9, 137.0, 144.1, 144.4, 153.8, 166.4. HRMS (m/z): [M + H]^+^ calcd for C_18_H_20_N_6_O_3_S_2_: 433.1111; found: 433.1106

###### *N*-(4-Methylbenzyl)-2-((1-phenyl-1*H*-tetrazol-5-yl)thio)-*N*-(4-sulfamoylphenyl)acetamide (**4o**)

Yield: 83%, M.P. = 87–90 °C, FTIR (ATR, cm^−1^): 3294 (N-H), 2924 (C–H), 1651 (C = O), 848. ^1^H-NMR (300 MHz, DMSO-*d_6_*): δ = 2.25 (3H, s, –CH_3_), 4.27 (2H, s, –CH_2_–), 4.92 (2H, s, –CH_2_–), 7.09 (4H, br.s., Methylbenzene), 7.46 (2H, br.s., –SO_2_NH_2_), 7.53 (2H, d, *J =* 8.4 Hz, 1,4-Disubstitutedbenzene), 7.67 (5H, br.s., Monosubstitutedbenzene), 7.86 (2H, d, *J =* 8.4 Hz, 1,4-Disubstitutedbenzene). ^13 ^C-NMR (75 MHz, DMSO-*d_6_*): δ = 21.1, 38.5, 52.7, 124.9, 127.5, 128.3, 129.2, 129.5, 130.6, 131.1, 133.5, 133.9, 137.0, 144.0, 144.3, 154.3, 166.2. HRMS (m/z): [M + H]^+^ calcd for C_23_H_22_N_6_O_3_S_2_: 495.1268; found: 495.1243

###### *N*-(4-Methylbenzyl)-2-(pyridin-2-ylthio)-*N*-(4-sulfamoylphenyl)acetamide (**4p**)

Yield: 77%, M.P. = 121–123 °C, FTIR (ATR, cm^−1^): 3408 (N-H), 2918 (C-H), 1645 (C = O), 848. ^1^H-NMR (300 MHz, DMSO-*d_6_*): δ = 2.25 (3H, s, –CH_3_), 3.93 (2H, s, -CH_2_-), 4.91 (2H, s, –CH_2_–), 7.09–7.13 (5H, m, Methylbenzene, Pyridine CH), 7.29 (1H, d, *J =* 8.1 Hz, Pyridine CH), 7.50 (2H, d, *J =* 8.4 Hz, 1,4-Disubstitutedbenzene), 7.62 (1H, td, *J_1_=*1.7 Hz, *J_2_=*7.7 Hz, Pyridine CH), 7.83 (2H, d, *J =* 8.4 Hz, 1,4-Disubstitutedbenzene). 8.36 (1H, d, *J =* 4.3 Hz, Pyridine CH). ^13 ^C-NMR (75 MHz, DMSO-*d_6_*): δ = 21.1, 33.5, 52.6, 120.4, 122.0, 127.3, 128.2, 129.1, 129.4, 134.4, 136.8, 137.1, 143.8, 145.1, 149.7, 157.5, 168.0. HRMS (m/z): [M + H]^+^ calcd for C_21_H_21_N_3_O_3_S_2_: 428.1097; found: 428.1092

###### 2-(Benzoxazol-2-ylthio)-*N*-(4-methylbenzyl)-*N*-(4-sulfamoylphenyl)acetamide (**4r**)

Yield: 85%, M.P. = 148–150 °C, FTIR (ATR, cm^−1^): 3388 (N-H), 2933 (C–H), 1666 (C = O), 856. ^1^H-NMR (300 MHz, DMSO-*d_6_*): δ = 2.25 (3H, s, –CH_3_), 4.24 (2H, s, –CH_2_–), 4.93 (2H, s, –CH_2_–), 7.07–7.11 (4H, m, Methylbenzene), 7.32–7.35 (2H, m, Benzoxazole CH), 7.55 (2H, d, *J =* 8.3 Hz, 1,4-Disubstitutedbenzene), 7.61–7.64 (2H, m, Benzoxazole CH), 7.88 (2H, d, *J =* 8.3 Hz, 1,4-Disubstitutedbenzene). ^13 ^C-NMR (75 MHz, DMSO-*d_6_*): δ = 21.1, 37.0, 52.9, 110.7, 118.7, 124.8, 125.1, 127.5, 128.3, 129.0, 129.5, 134.1, 136.9, 141.6, 144.1, 145.0, 151.7, 164.3, 166.5. HRMS (m/z): [M + H]^+^ calcd for C_23_H_21_N_3_O_4_S_2_: 468.1046; found: 468.1030

###### 2-(Benzothiazol-2-ylthio)-*N*-(4-methylbenzyl)-*N*-(4-sulfamoylphenyl)acetamide (**4s**)

Yield: 88%, M.P. = 170–172 °C, FTIR (ATR, cm^−1^): 3238 (N-H), 2918 (C-H), 1651 (C = O), 850. ^1^H-NMR (300 MHz, DMSO-*d_6_*): δ = 2.24 (3H, s, –CH_3_), 4.22 (2H, s, –CH_2_–), 4.93 (2H, s, –CH_2_–), 7.04–7.12 (4H, m, Methylbenzene), 7.37 (1H, td, *J_1_*=1.0 Hz, *J_2_*=7.7 Hz, Benzothiazole CH), 7.48 (1H, td, *J_1_*=1.1 Hz, *J_2_*=7.7 Hz, Benzothiazole CH), 7.56 (2H, d, *J =* 8.4 Hz, 1,4-Disubstitutedbenzene), 7.82 (1H, d, *J =* 8.0 Hz, Benzothiazole CH), 7.87 (2H, d, *J =* 8.4 Hz, 1,4-Disubstitutedbenzene), 8.00 (1H, d, *J =* 7.9 Hz, Benzothiazole CH). ^13 ^C-NMR (75 MHz, DMSO-*d_6_*): δ = 21.1, 37.3, 52.8, 121.6, 122.3, 125.0, 126.8, 127.4, 128.2, 129.1, 129.4, 134.2, 135.2, 136.9, 144.4, 144.5, 152.9, 166.3, 166.7. HRMS (m/z): [M + H]^+^ calcd for C_23_H_21_N_3_O_3_S_3_: 484.0818; found: 484.0796

###### 2-((5-Chlorobenzothiazol-2-yl)thio)-*N*-(4-methylbenzyl)-*N*-(4-sulfamoylphenyl)acetamide (**4t**)

Yield: 79%, M.P. = 109–111 °C, FTIR (ATR, cm^−1^): 3360 (N-H), 2927 (C-H), 1664 (C = O), 850. ^1^H-NMR (300 MHz, DMSO-*d_6_*): δ = 2.25 (3H, s, –CH_3_), 4.20 (2H, s, –CH_2_–), 4.94 (2H, s, –CH_2_–), 7.04–7.11 (4H, m, Methylbenzene), 7.40–7.43 (3H, m, -SO_2_NH_2_, Benzothiazole CH) 7.60 (2H, d, *J* = 8.3 Hz, 1,4-Disubstitutedbenzene), 7.85 (1H, d, *J =* 2.0 Hz, Benzothiazole CH), 7.90 (2H, d, *J =* 8.4 Hz, 1,4-Disubstitutedbenzene), 8.03 (1H, d, *J =* 8.6, Benzothiazole CH). ^13 ^C-NMR (75 MHz, DMSO-*d_6_*): δ = 21.1, 37.4, 52.8, 121.1, 123.7, 125.0, 127.6, 128.3, 129.3, 129.4, 131.7, 134.1, 134.2, 136.9, 144.0, 144.7, 153.7, 166.5, 169.2. HRMS (m/z): [M + H]^+^ calcd for C_23_H_20_ ClN_3_O_3_S_3_: 518.0428; found: 518.0393

###### 2-((5-Methoxybenzothiazol-2-yl)thio)-*N*-(4-methylbenzyl)-*N*-(4-sulfamoylphenyl)acetamide (**4u**)

Yield: 78%, M.P. = 82–84 °C, FTIR (ATR, cm^−1^): 3344 (N-H), 2933 (C-H), 1651 (C = O), 844. ^1^H-NMR (300 MHz, DMSO-*d_6_*): δ = 2.25 (3H, s, –CH_3_), 3.84 (3H, s, –OCH_3_), 3.84 (3H, s, –OCH_3_), 4.21 (2H, s, -CH_2_-), 4.94 (2H, s, -CH_2_-), 7.01 (1H, dd, *J_1_*=2.5 Hz, *J_2_*=8.8 Hz, Benzothiazole CH), 7.05–7.12 (4H, m, Methylbenzene), 7.32 (1H, d, *J =* 2.4 Hz, Benzothiazole CH), 7.43 (2H, br.s, –SO_2_NH_2_), 7.57 (2H, d, *J* = 8.4 Hz, 1,4-Disubstitutedbenzene), 7.84–7.89 (3H, m, Benzothiazole CH, 1,4-Disubstitutedbenzene). ^13 ^C-NMR (75 MHz, DMSO-*d_6_*): δ = 21.1, 37.3, 52.8, 56.0, 105.0, 114.2, 122.6, 126.8, 127.5, 128.3, 129.2, 129.5, 134.2, 136.9, 143.8, 144.7, 154.2, 159.1, 166.7, 167.2. HRMS (m/z): [M + H]^+^ calcd for C_24_H_23_N_3_O_4_S_3_: 514.0923; found: 514.0897.

### 2.2. MAO-A and MAO-B inhibition assay

Ampliflu™ Red (10-Acetyl-3,7-dihydroxyphenoxazine), *h*MAO-A, *h*MAO-B, peroxidase from horseradish, tyramine hydrochloride, H_2_O_2_, clorgiline and selegiline were acquired from Sigma-Aldrich (Steinheim, Germany) and retained under the proposed conditions by supplier. A Biotek Precision XS robotic system (USA) was used for all pipetting operations. Measurements were performed with the use of BioTek-Synergy H1 microplate reader (USA) based upon the fluorescence generated (excitation, 535 nm, emission, 587 nm) over a 30 min period, in which the fluorescence increased linearly.

Enzymatic assay was performed according to recent method pronounced by our research group^17^^,^^20–22^. Control, blank and all concentrations of obtained compounds were tested in quadruplicate and inhibition percent was calculated with following equation:

$$\text{\% Inhibition}=\frac{\left({\mathrm{FC}}_{t2\;}-{\text{ FC}}_{t1})\;-\;({\text{FI}}_{t2}-\;{\mathrm{FI}}_{t1}\right)}{{\mathrm{FC}}_{t2\;}-{\text{ FC}}_{t1}}\;\times \;100$$

FCt_2_: Fluorescence of a control well measured at t_2_ time, FCt_1_: Fluorescence of a control well measured at t_2_ time, FIt_2_: Fluorescence of an inhibitor well measured at t_2_ time, FIt_1_: Fluorescence of an inhibitor well measured at t_1_ time,

The IC_50_ values were calculated using a dose-response curve achieved by plotting the percentage inhibition versus the log concentration using GraphPad ‘PRISM’ software (version 5.0). The results were showed as mean ± SD.

### 2.3. Enzyme kinetic studies

The same materials were used in the MAO inhibition assay. The most active compounds 4i and 4t determined according to the result of the MAO inhibition assay were experienced in three different concentrations of IC_50_/2, IC_50_ and 2(IC_50_) in accordance with the assay assigned in our final study.^17^^,^^20–22^. All processes were evaluated in quadruplicate. The results were analysed as Lineweaver–Burk plots by means of Microsoft Office Excel 2013. The *V*_max_ values of the Lineweaver–Burk plots were replotted versus the inhibitor concentration, and the *K_i_* values were determined from the *x*-axis intercept as −*K_i_*.

### 2.4. Cytotoxicity assay

The NIH/3T3 mouse embryonic fibroblast cell line (ATCC^®^CRL-1658 ™, London, UK) was used for cytotoxicity assays. The incubation period of NIH/3T3 cells was based on the supplier's recommendation. NIH/3T3 cells were seeded at 1 × 10^4^ cells into each well of 96-well plates. MTT assay was carried out in accordance with the standards previously described manner^23^^,^^24^. The compounds were tested between 1 and 0.000316 mM concentrations. Inhibition % for each concentration was calculated according to the following formula and IC_50_ values were reported by plotting the% inhibition dose response curve against the compound concentrations tested.^23–25^.

% inhibition=100−(mean sample×100/mean solvent)

### 2.5. Prediction of ADME parameters and BBB permeability

Physicochemical parameters were performed with the use of *QikProp 4.8* software^26^ to predict pharmacokinetic profiles and BBB permeability of obtained compounds (**4a–4u**).

### 2.6. Molecular docking

A structure based *in silico* procedure was applied to discover the binding modes of compound **4i** to *h*MAO-B enzyme active site. The crystal structures of *h*MAO-B (PDB ID: 2V5Z)^27^, which was crystallised with safinamide, were retrieved from the Protein Data Bank server (www.pdb.org).

The structures of ligands were built using the *Schrödinger Maestro*^28^ interface and then were submitted to the *Protein Preparation Wizard* protocol of the *Schrödinger Suite 2016 Update 2*^29^. The ligands were prepared by the *LigPrep 3.8*^30^ to assign the protonation states at pH 7.4 ± 1.0 and the atom types, correctly. Bond orders were assigned, and hydrogen atoms were added to the structures. The grid generation was formed using *Glide 7.1*^31^. The grid box with dimensions of 20 Å × 20 Å × 20 Å was centred in the vicinity of the flavin (FAD) N5 atom on the catalytic site of the protein to cover all binding sites and neighbouring residues^32–34^. Flexible docking runs were performed with single precision docking mode (SP).

## 3. Result and discussion

### 3.1. Chemistry

**Scheme 1.**
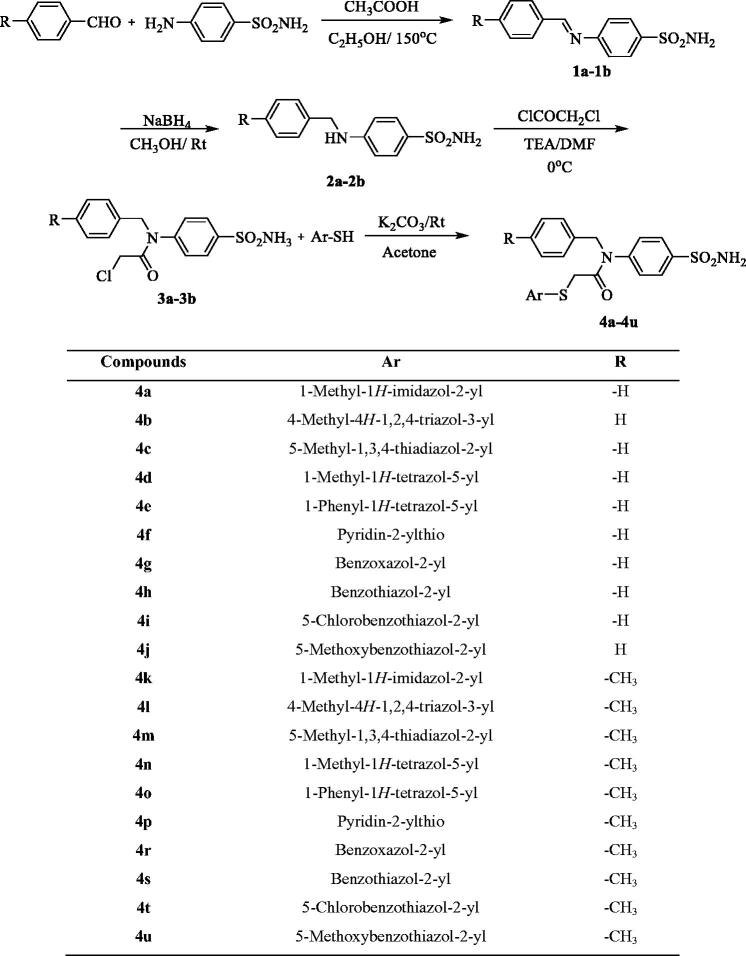
Synthesis way of the compounds **4a**–**4u**.

Various compounds, labelled **4a** to **4u**, were synthesised as outlined in Scheme 1. Initially, Schiff bases were prepared through the reaction of benzaldehyde (or 4-methylbenzaldehyde) and sulphanilamide. Then, benzylamine derivatives (**2a**, **2b**) were obtained by a reduction reaction. Acetylation of the benzylamine derivatives (**2a**, **2b**) gave compounds **3a** and **3b**. Finally, the target compounds (**4a**–**4u**) were obtained through a substitution reaction using acetylated benzylamine (**3a**, **3b**) and corresponding heterocyclic thiols.

The synthesised compounds were elucidated by instrumental analyses such as infra-red spectroscopy (IR), mass spectrometry (MS), and nuclear magnetic resonance (NMR). The N-H bond of the sulphonamide group appeared as IR bands between 3238 and 3483 cm^−1^, and as a singlet between 7.30 and 7.60 ppm on the ^1^H-NMR spectrum. The presence of the carbonyl group was shown by IR bands between 1645 cm^−1^ and 1666 cm^−1^, and a ^13 ^C-NMR peak over 160 ppm. The CH_2_ group bound to the nitrogen atom gave a singlet ^1^H-NMR peak around 4.90 ppm, and a ^13 ^C-NMR peak over 50 ppm. The other CH_2_ group between the carbonyl and sulphur groups was recorded in ^1^H-NMR peak around 4.20 ppm and a ^13 ^C-NMR peak between 33.4 and 38.5 ppm. The carbons of aromatic groups were observed from 105.0 to 166.7 ppm in the ^13 ^C-NMR spectrum, and the protons of the same groups were between 6.90 and 8.51 ppm in the ^1^H-NMR spectrum. In mass spectroscopy, the masses were found to differ by at most 5 ppm from the expected masses.

### 3.2. MAO inhibition

All the obtained benzylamine-sulphonamide derivatives **4a**–**4u** were investigated for their inhibitory activity against MAO isoforms using a previously described *in vitro* fluorometric method, which is based on the detection of H_2_O_2_ in a horseradish peroxidase-coupled reaction using 10-acetyl-3,7-dihydroxyphenoxazine (Amplex Red reagent)^17^^,^^20–22^.

The assay was carried out in two steps. The first step was carried out using 10^−3^ and 10^−4 ^M concentrations of all synthesised compounds and reference agents, namely selegiline and clorgiline. The enzyme activity results of first step are presented in Table 1. Then, the selected compounds that displayed more than 50% inhibitory activity at 10^−3^ and 10^−4 ^M concentrations were further tested, along with reference agents, at concentrations of 10^−5^ to 10^−9 ^M. The IC_50_ values of the test compounds and reference agents are presented in Table 2.

**Table 1.** % Inhibition of the synthesized compounds, clorgiline and selegiline against MAO-A and MAO-B enzymes.

Compounds	MAO-A % Inhibition		MAO-B % Inhibition	
	10^−3^ M	10^−4^ M	10^−3^ M	10^−4^ M
**4a**	35.123 ± 0.989	26.505 ± 0.856	92.298 ± 1.108	47.022 ± 0.958
**4b**	37.588 ± 0.714	20.125 ± 0.621	90.256 ± 1.304	86.301 ± 1.294
**4c**	40.577 ± 0.749	24.110 ± 0.610	91.102 ± 1.250	48.301 ± 0.855
**4d**	39.180 ± 0.650	29.515 ± 0.489	91.578 ± 1.247	79.679 ± 1.459
**4e**	41.528 ± 0.899	28.016 ± 0.714	90.120 ± 1.008	48.363 ± 0.721
**4f**	46.022 ± 0.863	30.114 ± 0.627	90.585 ± 1.388	83.327 ± 1.259
**4g**	47.199 ± 0.979	32.233 ± 0.621	94.662 ± 1.345	47.338 ± 0.963
**4h**	39.108 ± 0.821	20.332 ± 0.608	96.205 ± 1.105	48.755 ± 0.879
**4i**	56.321 ± 0.996	40.456 ± 0.782	94.859 ± 1.405	91.755 ± 1.258
**4j**	48.177 ± 0.825	37.362 ± 0.679	90.839 ± 1.245	45.097 ± 0.958
**4k**	39.347 ± 0.701	30.327 ± 0.582	79.521 ± 0.957	39.011 ± 0.702
**4l**	42.299 ± 0.630	25.208 ± 0.712	74.204 ± 1.052	43.798 ± 0.882
**4m**	37.356 ± 0.850	31.088 ± 0.729	76.308 ± 1.004	40.112 ± 0.799
**4n**	40.158 ± 0.970	21.525 ± 0.632	72.158 ± 0.959	38.254 ± 0.638
**4o**	44.158 ± 0.877	28.654 ± 0.509	68.332 ± 0.824	30.255 ± 0.721
**4p**	45.203 ± 0.971	24.854 ± 0.792	70.501 ± 1.071	35.619 ± 0.622
**4r**	42.388 ± 0.821	32.749 ± 0.697	67.550 ± 0.957	33.126 ± 0.798
**4s**	38.216 ± 0.734	32.997 ± 0.697	72.651 ± 1.002	40.299 ± 0.835
**4t**	52.628 ± 0.987	38.320 ± 0.503	92.588 ± 1.129	88.565 ± 1.204
**4u**	36.775 ± 0.678	29.859 ± 0.539	65.127 ± 0.985	35.956 ± 0.726
**Clorgiline**	99.411 ± 1.955	98.257 ± 1.824	–	–
**Selegiline**	–	–	98.258 ± 1.052	96.107 ± 1.165

**Table 2.** IC_50_ values of **4b**, **4d**, **4f**, **4i**, **4t** and selegiline against MAO-B.

Compounds	MAO-B % Inhibition							IC_50_ (µm)
	10^−3^ M	10^−4^ M	10^−5^ M	10^−6^ M	10^−7^ M	10^−8^ M	10^−9^ M	
**4b**	90.256 ± 1.304	86.301 ± 1.294	82.456 ± 1.107	76.901 ± 1.266	45.325 ± 0.974	32.608 ± 0.728	20.417 ± 0.683	0.188 ± 0.008
**4d**	91.578 ± 1.247	79.679 ± 1.459	76.245 ± 1.057	70.896 ± 1.006	47.498 ± 0.957	29.670 ± 0.723	15.157 ± 0.602	0.127 ± 0.005
**4f**	90.585 ± 1.388	83.327 ± 1.259	76.311 ± 1.114	73.203 ± 1.058	48.645 ± 0.957	32.499 ± 0.721	20.874 ± 0.623	0.146 ± 0.006
**4i**	94.859 ± 1.405	91.755 ± 1.258	78.629 ± 1.106	72.455 ± 1.115	65.167 ± 0.985	46.508 ± 0.892	24.475 ± 0.627	0.041 ± 0.001
**4t**	92.588 ± 1.129	88.565 ± 1.204	80.497 ± 1.157	74.698 ± 1.009	60.755 ± 1.164	36.131 ± 0.862	20.971 ± 0.594	0.065 ± 0.002
**Selegiline**	99.387 ± 1.385	95.629 ± 1.456	86.205 ± 1.200	78.324 ± 1.108	66.871 ± 1.056	42.875 ± 0.865	16.748 ± 0.596	0.037 ± 0.001

According to the enzyme inhibition results, none of the synthesised compounds showed a significant activity against *h*MAO-A enzyme. All of the obtained compounds displayed selective inhibition on *h*MAO-B. At 1 0 ^−3 ^M concentration, all of the compounds showed more than 50% inhibitory activity. Compounds **4b**, **4d**, **4f**, **4i** and **4t** could pass the second step of enzyme activity assay and the IC_50_ values of them were calculated by performing enzyme inhibition study at 10^−5^–10^−9 ^M concentration. The most active compounds, **4i** and **4t**, exhibited IC_50_ values of 0.041 ± 0.001 µM and 0.065 ± 0.002 µM, respectively, against *h*MAO-B, while the reference agent, selegiline, had an IC_50_ of 0.037 ± 0.001 µM.

These findings from the screening of inhibitory activities against *h*MAO-B revealed that the compounds containing 5-chlorobenzothiazole exhibited more potent inhibitory activity than the other obtained compounds as in the previously synthesised and reported **BB-4h** derivative, which has a 5-chlorobenzothiazole ring. Moreover, the increased inhibitory activity of the synthesised compounds, compared to that of **BB-4h**, is likely due to the contribution of the sulphonamide group, which displaced the fluorine group, and the removal of the nitro group from the structure.

### 3.3. Kinetic studies of enzyme inhibition

**Figure 2.**
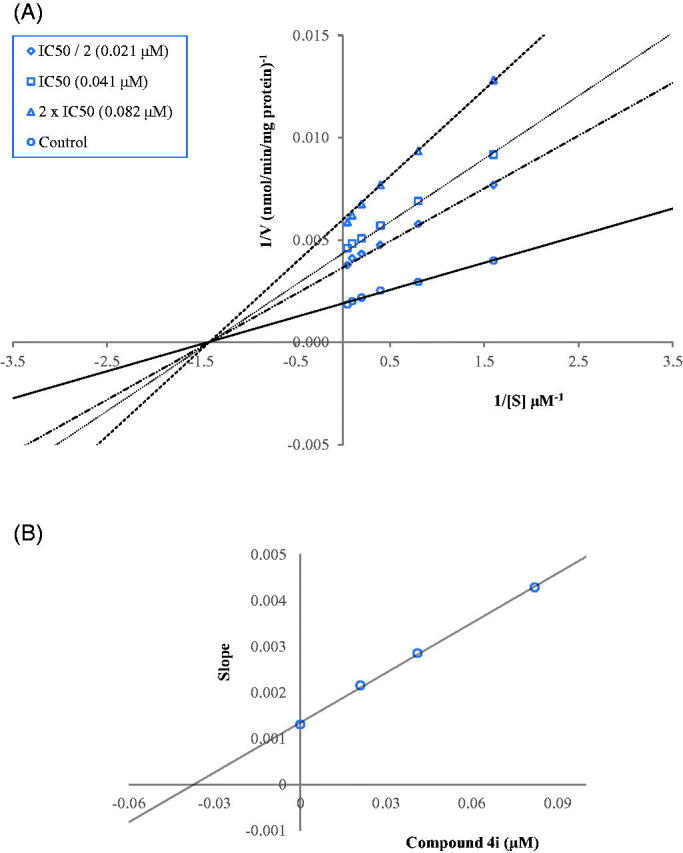
(A) Lineweaver–Burk plots for the inhibition of *h*MAO-B by compound **4i**. [S], substrate concentration (µM); V, reaction velocity (nmol/min/mg protein). Inhibitor concentrations are shown at the left. (B) Secondary plot for the calculation of the steady-state inhibition constant (K_i_) of compound **4i**. K_i_ was calculated as 0.036 µM.

**Figure 3.**
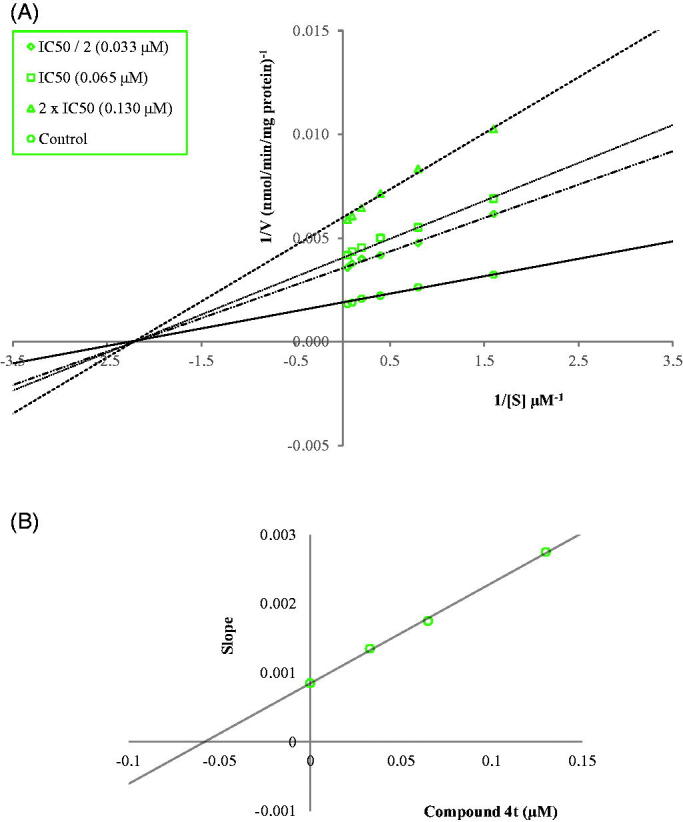
(A) Lineweaver–Burk plots for the inhibition of *h*MAO-B by compound **4t**. [S], substrate concentration (µM); V, reaction velocity (nmol/min/mg protein). Inhibitor concentrations are shown at the left. (B) Secondary plot for the calculation of the steady-state inhibition constant (K_i_) of compound **4t**. K_i_ was calculated as 0.055 µM.

Enzyme kinetics studies were performed to determine the mechanism of *h*MAO-B inhibition by using a procedure similar to that of the MAO inhibition assay. Compounds **4i** and **4t**, which were found to be the most potent agents, were included in these studies. In order to estimate the type of inhibition of these compounds, linear Lineweaver-Burk graphs were used. Substrate velocity curves in the absence and presence of compounds **4i** and **4t** were recorded. These compounds were prepared at concentrations of IC_50_/2, IC_50_, and 2(IC_50_) for enzyme kinetic studies. In each case, the initial velocity measurements were obtained at different substrate (tyramine) concentrations ranging from 20 μM to 0.625 μM. The secondary plots of slope (K_m_/V_max_) versus varying concentrations (0, IC_50_/2, IC_50_, and 2(IC_50_)) were created to calculate the K_i_ (intercept on the x-axis) value of these compounds. The graphical analyses of steady-state inhibition data for compounds **4i** and **4t** are shown in Figures 2 and 3.

The type of inhibition can be determined as either reversible or irreversible by using the Lineweaver-Burk plots. The reversible inhibition type can be classified as mixed-type, uncompetitive, competitive, or non-competitive^17^^,^^20–22^. According to Lineweaver–Burk plots, a graph that shows parallel lines without any cross-overs is observed in the uncompetitive type of inhibition. For mixed-type inhibition, a graph with lines that do not intersect at the x-axis or the *y*-axis is formed. Competitive inhibition is seen if the lines intersect on the *y*-axis, and the slopes and *x*-intercepts are different. On the contrary, non-competitive inhibition has the opposite result: the plotted lines have the same x-intercept but there are diverse slopes and *y*-intercepts. Therefore, as shown in Figures 2 and 3, compounds **4i** and **4t** are reversible and non-competitive inhibitors with similar inhibition features as the substrates. K_i_ values for compounds **4i** and **4t** were calculated as 0.036 and 0.055 μM, respectively, for the inhibition of *h*MAO-B.

Irreversible enzymatic inhibition involves covalent interactions between the substrate and the enzyme. In contrast, there are non-covalent interactions such as hydrophobic interactions, ionic bonds, and hydrogen bonds involved in reversible inhibition. In this type of inhibition, inhibitors bind to the enzymes without forming any chemical bonds; thus, the enzyme-inhibitor complex could be separated quickly because non-covalent interactions can form rapidly and break easily. Furthermore, reversible inhibitors have a lower risk of side effects than irreversible inhibitors owing to their non-covalent binding ability. Consequently, compounds **4i** and **4t**, whose inhibition types were determined to be reversible and non-competitive, have pharmaceutical importance in contrast to irreversible hydrazine-type MAO inhibitors.

### 3.4. Cytotoxicity

Compounds **4i** and **4t** displayed potent *h*MAO-B inhibition profiles and were further tested for toxicity using the MTT assay in the NIH3T3 cell line; the IC_50_ values of compounds **4i** and **4t** are shown in Table 3. Both compounds showed an IC_50_ value of >1000 µM against NIH3T3 cells, which was significantly higher than their IC_50_ values (0.041 and 0.065 µM) against *h*MAO-B. Consequently, compounds **4i** and **4t** were found to be non-cytotoxic at their effective concentrations against *h*MAO-B. This result further increases the biological importance of the compounds.

**Table 3.** The IC_50_ value of the compounds **4i** and **4t** against NIH/3T3 cell line.

Compounds	IC_50_ (µM) NIH/3T3 cell line	IC_50_ (µM) MAO-B enzyme
**4i**	>1000	0.041 ± 0.001
**4t**	>1000	0.065 ± 0.002

### 3.5. Prediction of ADME parameters and BBB permeability

Intrinsic pharmacological activity and low toxicological effects are not sufficient for a compound to become a drug nominee^35^. Most new drug nominees fail in clinical trials due to their reduced absorption, distribution, metabolism, and excretion (ADME) properties. These late-stage failures result in increased drug development costs^36^. The ability to identify problematic issues early can dramatically reduce the amount of wasted time and funds, and can streamline the overall development process. Therefore, the pharmacokinetic properties of new drug candidates are very important, and it is beneficial to assess them as early as possible in the drug development process^37^. ADME estimation can be used to focus on precursor compound optimisation thereby improving the preferred properties of a compound^38^. Predictions of ADME parameters of the obtained compounds (**4a**–**4u**) were performed using *QikProp 4.8* software^26^. The violations of Jorgensen’s “Rule of Three”^39^ and Lipinski’s rule of five^40^, which assess the ADME properties of new drug nominees, are crucial for the optimisation of a biologically active compound. The calculated ADME parameters, including molecular weight (MW), number of rotatable bonds (RB), dipole moment (DM), molecular volume (MV), number of hydrogen donors (DHB), number of hydrogen acceptors (AHB), polar surface area (PSA), octanol/water partition coefficient (log P), aqueous solubility (log S), apparent Caco-2 cell permeability (PCaco), number of likely primer metabolic reactions (PM), percent of human oral absorption (%HOA), and the violations of the rules of three (VRT) and five (VRF) are presented in Table 4. In keeping with Jorgensen’s “Rule of Three” and Lipinski’s rule of five, the obtained compounds (**4a**–**4u**) are in accordance with the set parameters as they did not cause more than one violation.

**Table 4.** Calculated ADME parameters of compounds **4a**–**4u**.

Comp.	MW	RB	DM	MV	DHB	AHB	PSA	logP	logS	PCaco	logBB	PMDCK	PM	%HOA	VRF	VRT
**4a**	416.51	8	9.694	1233.2	2	9	107.701	2.369	−4.323	141.478	−1.784	90.865	2	79.311	0	0
**4b**	417.5	8	12.125	1219.7	2	9.5	125.131	1.753	−4.058	57.916	−2.181	34.702	2	68.759	0	0
**4c**	434.55	8	7.1	1198.4	2	9.5	113.396	2.082	−3.755	148.509	−1.482	183.53	4	78.006	0	0
**4d**	418.49	8	11.923	1207.8	2	10.5	143.808	0.987	−3.669	26.232	−2.537	14.803	2	58.119	0	0
**4e**	480.56	8	9.94	1382	2	10.5	139.593	2.334	−4.976	40.763	−2.509	21.026	2	69.43	0	0
**4f**	413.51	8	6.179	1177.5	2	8.5	95.543	2.553	−3.681	271.256	−1.295	217.606	3	85.444	0	0
**4g**	453.53	8	5.536	1311	2	9.5	115.869	2.718	−5.106	117.016	−2.012	81.775	3	79.88	0	0
**4h**	469.59	8	6.263	1346.1	2	9	104.573	3.385	−5.787	158.686	−1.785	199.449	3	86.149	0	1
**4i**	504.04	8	4.786	1390	2	9	104.572	3.865	−6.5	158.573	−1.651	491.011	3	75.996	1	1
**4j**	499.62	9	5.132	1411.6	2	9.75	112.58	3.472	−5.883	187.797	−1.785	246.266	4	87.968	0	1
**4k**	430.54	8	10.106	1292.1	2	9	107.701	2.651	−4.837	141.478	−1.84	90.865	3	80.962	0	0
**4l**	431.53	8	12.578	1278.7	2	9.5	125.131	2.032	−4.565	57.916	−2.248	34.702	3	70.393	0	0
**4m**	448.57	8	10.406	1288.9	2	9.5	120.374	2.234	−4.621	75.534	−2.01	64.7	5	73.642	0	0
**4n**	432.51	8	12.376	1266.8	2	10.5	143.808	1.261	−4.166	26.232	−2.615	14.803	3	59.725	0	0
**4o**	494.59	8	9.925	1440.9	2	10.5	139.593	2.617	−5.493	40.763	−2.579	21.026	3	71.09	0	0
**4p**	427.54	8	6.246	1236.4	2	8.5	95.543	2.831	−4.186	271.256	−1.344	217.606	4	87.072	0	0
**4r**	467.56	8	5.601	1369.9	2	9.5	115.869	3.004	−5.628	117.016	−2.067	81.775	4	81.551	0	0
**4s**	483.62	8	6.255	1405	2	9	104.573	3.673	−6.316	158.686	−1.836	199.449	4	87.839	0	1
**4t**	518.06	8	4.857	1448.9	2	9	104.572	4.155	−7.033	158.573	−1.702	491.011	4	77.697	1	1
**4u**	513.64	9	4.95	1470.6	2	9.75	112.58	3.76	−6.411	187.797	−1.834	246.266	5	76.7	1	1

MW: Molecular weight RB: Number of rotatable bonds DM: Computed dipole moment MV: Total solvent-accessible volume DHB: Estimated number of hydrogen bond donors AHB: Estimated number of hydrogen bond acceptors PSA: Van der Waals surface area of polar nitrogen and oxygen atoms and carbonyl carbon atoms logP: Predicted octanol/water partition coefficient logS: Predicted aqueous solubility PCaco: Predicted apparent Caco-2 cell permeability logBB: Predicted brain/blood partition coefficient PMDCK: Predicted apparent MDCK cell permeability PM: Number of likely metabolic reactions %HOA: Predicted human oral absorption percent VRF: Number of violations of Lipinski’s rule of five. The rules are: MW < 500, logP < 5, DHB ≤ 5, AHB ≤ 10, Positive PSA value. VRT: Number of violations of Jorgensen’s rule of three. The three rules are: logS > -5.7, PCaco > 22 nm/s, PM < 7.

Drugs that specifically target the CNS must first pass the blood–brain barrier (BBB). Although the BBB is protective in nature, the use of drug candidates with CNS effects in a clinical setting is unlikely if such drug molecules are unable to penetrate it. Therefore, this feature should be examined earlier on in the drug discovery process. Accordingly, predicting the BBB permeability of new compounds is of great significance^41^. Thereby, the BBB permeability of the obtained compounds (**4a**–**4u**) was also evaluated using *QikProp 4.8* software^26^. Brain/blood partition coefficient (logBB) and apparent MDCK cell permeability (PMDCK) were calculated for this purpose. In keeping with the software estimates, the PMDCK values of <25 and >500 nm/sec are advised as poor and great for non-active transport of compounds. In order to assess for a compound’s capacity to pass through the BBB, logBB is the other significant parameter to consider, with recommended values between −3 and +1.2. The PMDCK and logBB values of the synthesised compounds are within the advised ranges as shown in Table 4. Thus, it can be postulated that the obtained compounds are capable of crossing the BBB, which is crucial for CNS-associated drugs.

Considering the results of the ADME and BBB permeability studies, the synthesised compounds have pharmacokinetic profiles that may be appropriate for clinical use.

### 3.6. Molecular docking

As observed in the MAO inhibition assay studies, compounds **4i** and **4t** were found to be the most active derivatives in the *h*MAO-B enzyme inhibition series. Furthermore, compound **4i** was determined to be the most potent agent with an IC_50_ value of 0.041 ± 0.001 µM; hence, docking studies were carried out to evaluate its inhibition capability *in silico*. Using the X-ray crystal structure of *h*MAO-B (PDB ID: 2V5Z)^27^, docking studies were performed and the binding modes of compound **4i** were assigned. Also, this compound was subjected to the molecular docking procedure with the X-ray crystal structure of *h*MAO-A (PDB ID: 2Z5X) to compare its binding modes on *h*MAO-A and *h*MAO-B enzymes. Unfortunately, it was determined that compound **4i** did not be settle down to the active site of *h*MAO-A enzyme (data not shown). Thus, no important and significant interactions were observed. Actually, this evidence is consistent with *in vitro* enzyme inhibition assay and supports the selective effect of compound **4i** and the other derivatives in the series on *h*MAO-B enzyme.

**Figure 4.**
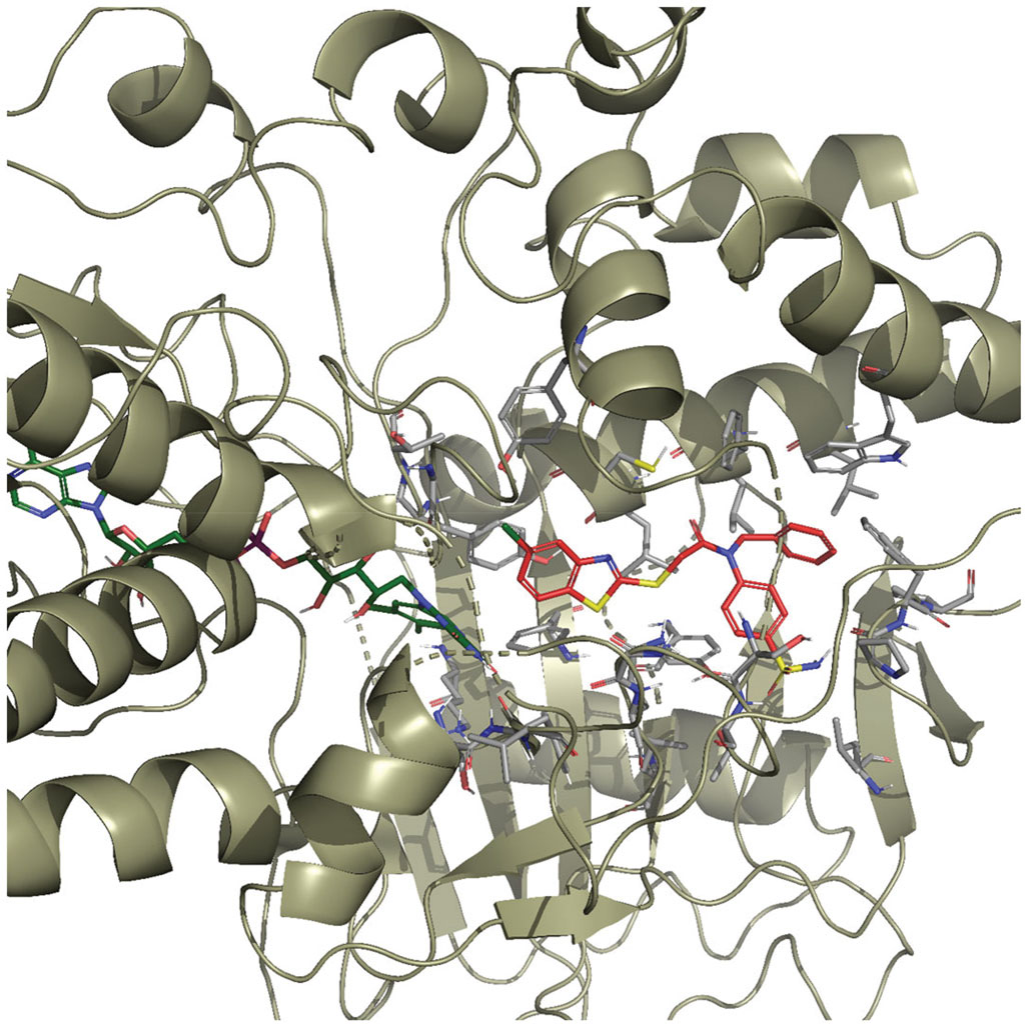
The three-dimensional pose of compound **4i** in the active region of *h*MAO-B (PDB ID: 2V5Z). The important residues in the active site and this compound are presented by tube model and coloured with grey and red, respectively.

**Figure 5.**
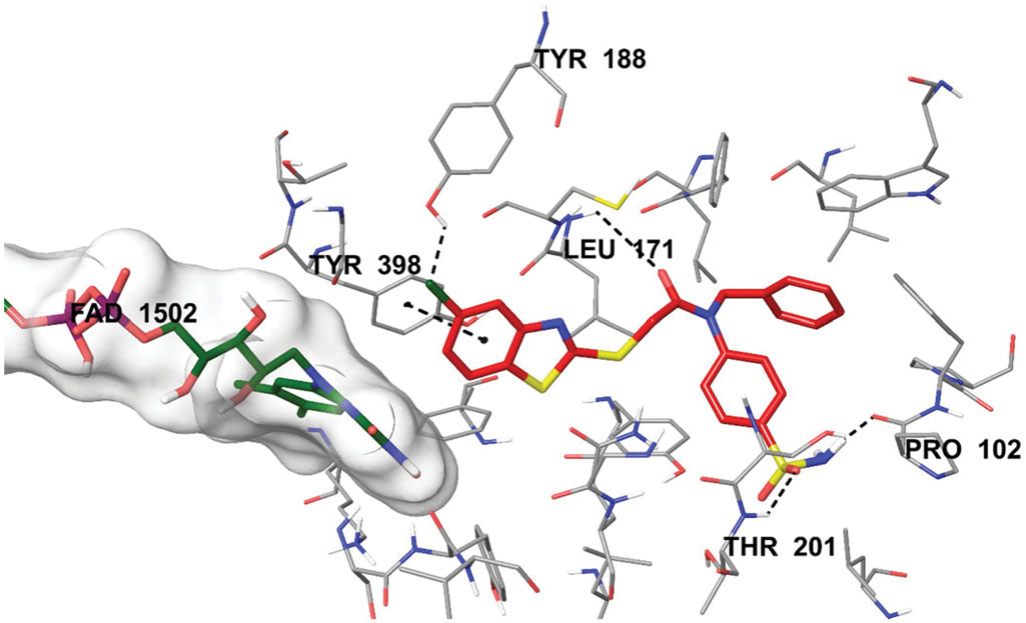
The three-dimensional interacting mode of compound **4i** in the active region of *h*MAO-B. The inhibitor and the important residues in the active site of the enzyme are presented by tube model. The FAD molecule is coloured dark green with tube model.

**Figure 6.**
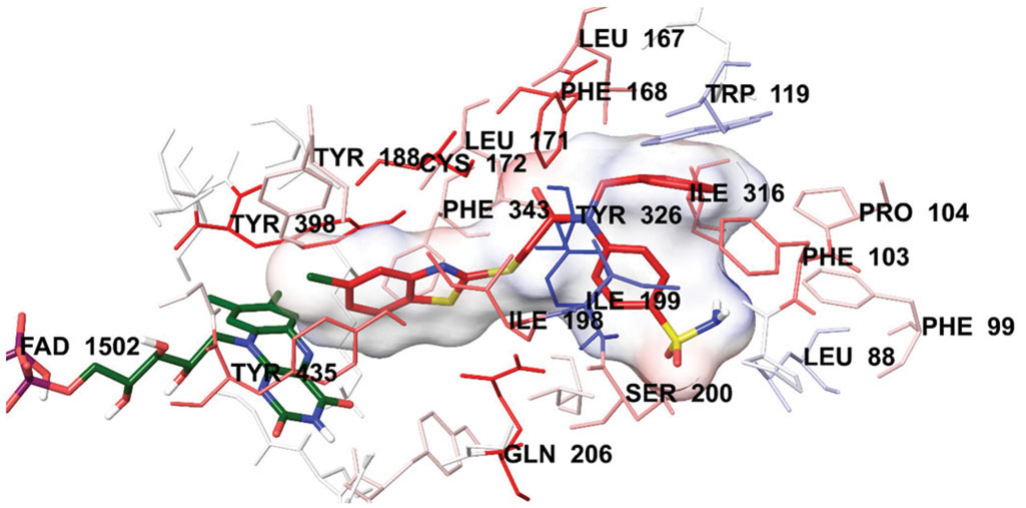
The van der Waals interaction of compound **4i** with active region of *h*MAO-B. The active ligand has a lot of favourable van der Waals interactions (red and pink).

**Figure 7.**
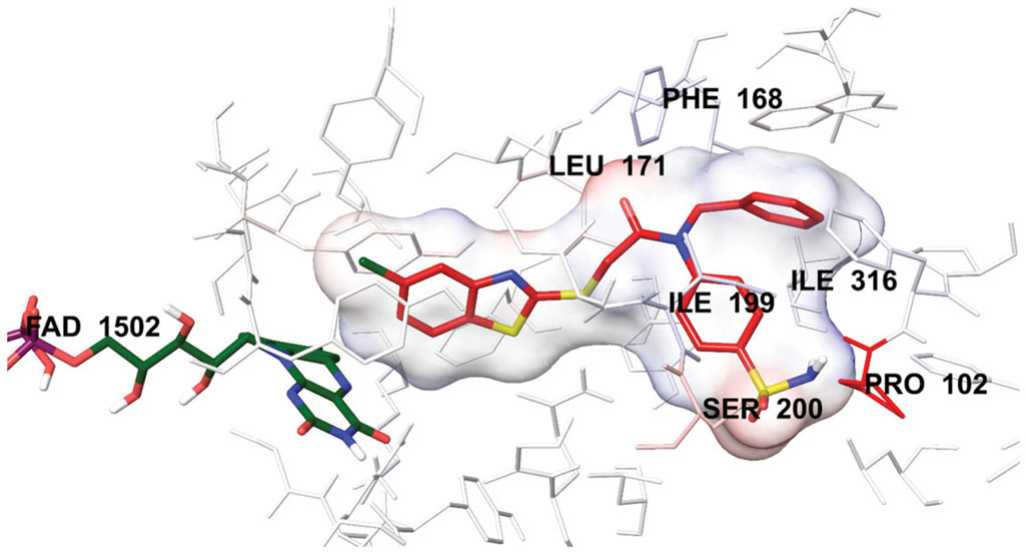
The electrostatic interaction of compound **4İ** with active region of *h*MAO-B. The residues are coloured (blue, red, and pink) according to the distance from ligand by Per-Residue Interaction panel.

The docking poses of this compound are presented in Figures 4–7. Compound **4i** adequately binds to the amino acid residues lining the cavity of *h*MAO-B enzyme and is located very near the FAD cofactor. When analysing the docking poses of this compound, it is clear that there is a π-π interaction, formation of three hydrogen bonds, and formation of a halogen bond. Compound **4i** has a sulphonamide group at the 4th position of the phenyl ring. This group is essential for polar interactions. The amino moiety of sulphonamide forms a hydrogen bond with the carbonyl of Pro102. In addition, there is another hydrogen bond between the oxygen atom of sulphonamide and the amino group of Thr201. As mentioned above, one of the main structural modifications of **BB-4b**, as previously reported by our research group^2^, is the substitution of the fluorine atom with a sulphonamide group (Figure 1). All the detected interactions of the sulphonamide group prove that the structural modification of compound **BB-4b** is a suitable approach. The addition of the sulphonamide group made a positive contribution to the MAO enzyme inhibition profile.

Another formation of hydrogen bond is observed between the carbonyl of the amide group in the structure and the amino group of Leu171. Compound **4i** has a benzothiazole ring as a heterocyclic ring. The benzene on the benzothiazole ring interacts with the phenyl of Thr398. Interaction with the Thr398 amino acid is very important in terms of catalytic activity, and the binding of inhibitor candidates in the substrate cavity of the MAO-B enzyme. This finding indicates that compound **4i** binds very effectively to the MAO-B enzyme active site.

The main structural difference between compound **4i** and the other compounds in the series is that it carries a chlorine atom at the 5th position of the benzothiazole ring. It is clearly observed in Figure 5 that this halogen atom establishes a halogen bond with the hydrogen of the hydroxyl group of Tyr188. This additional interaction ensures that it binds more strongly to the active site. Furthermore, all these interactions explain why compound **4i** exhibits a stronger inhibition profile than the other compounds.

In order to analyse the contribution of van der Waals and electrostatic interactions in binding to the enzyme active site, docking studies were performed using *Glide*, according to the Per-Residue Interaction panel. Figures 6 and 7 present the van der Waals and electrostatic interactions of compound **4i**. As shown in the figures, this compound has favourable van der Waals interactions with Leu88, Phe99, Phe103, Pro104, Tyr119, Leu167, Phe168, Leu171, Cys172, Tyr188, Ile198, Ile199, Ser200, Gln206, Ile316, Tyr326, Phe343, Tyr398 and Tyr435, which are displayed in pink and red colours as described in the user guide of *Glide*^31^. Similarly, promising electrostatic contributions of compound **4i** have been determined with Pro102, Phe168, Leu171, Ile199, Ser200 and Ile316 amino acids.

## 4. Conclusion

In conclusion, a new series of benzylamine-sulphonamide derivatives were designed, and their inhibition profile of MAO isozymes was evaluated. None of the synthesised compounds displayed a remarkable enzyme activity on *h*MAO-A enzyme. All of the compounds showed selectivity against *h*MAO-B enzyme. Among the obtained compounds, labelled **4i** and **4t** derivatives were found to be most active agents. Compound **4i**, which contained 5-chlorobenzothiazole ring, was determined to be the most effective inhibitor candidate with an IC_50_ value of 0.041 ± 0.001 µM. It is thought that the 5-chlorobenzothiazole ring and sulphonamide groups were very essential for inhibiting *h*MAO-B enzyme by docking studies. Hence, these findings showed that the new benzylamine-sulphonamide derivatives inhibited *h*MAO-B enzyme and suggested that benzylamine-sulphonamide derivatives could be improved in future studies with modifications to design and gain more potent MAO enzyme inhibitor candidates.

## Supplementary Material

Supplemental Material
