# Supplementary material for: Synthesis, in vitro enzyme activity and molecular docking studies of new benzylamine-sulfonamide derivatives as selective MAO-B inhibitors
Source: J Enzyme Inhib Med Chem. 2020 Jun 30;35(1):1422–32. doi: 10.1080/14756366.2020.1784892 (PMC7821958; doi:10.1080/14756366.2020.1784892)
Supplement: Supplemental Material [file IENZ_A_1784892_SM2422.pdf]

SUPPLEMENTARY MATERIAL

Synthesis, *in vitro* enzyme activity and molecular docking studies of new benzylamine-sulfonamide derivatives as selective MAO-B inhibitors

Begüm Nurpelin Sağlık[a,b*], Derya Osmaniye[a,b], Ulviye Acar Çevik[a,b], Serkan Levent[a,b], Betül Kaya Çavuşoğlu[c], Özlem Atlı Eklioğlu[d], Yusuf Özkay[a,b], Ali Savaş Koparal[e], Zafer Asım Kaplancıklı[a]

[a] *Department of Pharmaceutical Chemistry, Faculty of Pharmacy, Anadolu University, 26470 Eskişehir, Turkey*

[b] *Doping and Narcotic Compounds Analysis Laboratory, Faculty of Pharmacy, Anadolu University, 26470 Eskişehir, Turkey*

[c] *Department of Pharmaceutical Chemistry, Faculty of Pharmacy, Zonguldak Bülent Ecevit University, 67600 Zonguldak, Turkey*

[d] *Department of Pharmaceutical Toxicology, Faculty of Pharmacy, Anadolu University, 26470 Eskişehir, Turkey*

[e] *Open Education Faculty, Anadolu University, 26470 Eskişehir, Turkey*

* Corresponding author.

E-mail address: bnsaglik@anadolu.edu.tr (B.N. Sağlık).

Tel: +90-222-3350580/3774 *Fax*: +90-222-3350750.

Address: Anadolu University, Faculty of Pharmacy, Department of Pharmaceutical Chemistry, 26470, Eskişehir, Turkey.

DOPNALAB

Item	Value
Acquired Date&Time	3.05.2018 12:54:20
Acquired by	System Administrator
Filename	C:\Users\dopnalab\Desktop\derya\ydo sensi\ydo-11.ispd
Spectrum name	ydo-11
Sample name	ydo-1
Sample ID	
Option	
Comment	
No. of Scans	10
Resolution	4 [cm-1]
Apodization	Happ-Genzel

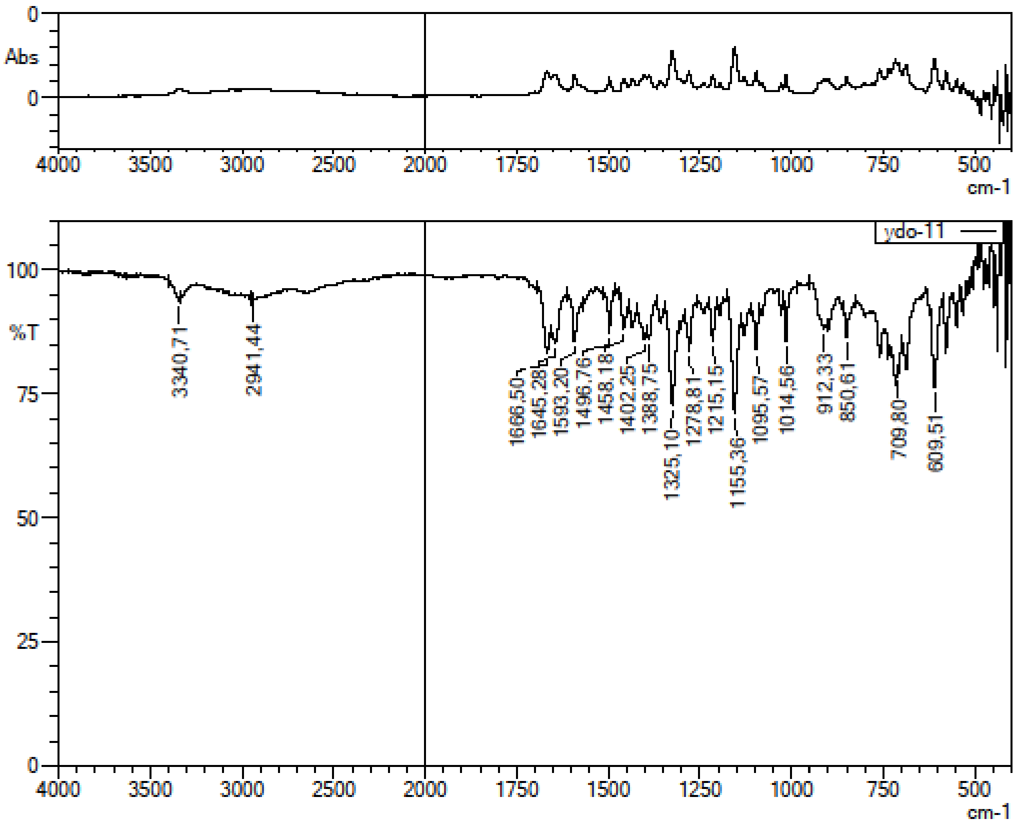

Spectra 1. IR spectra of compound **4a**

Data File: C:\LabSolutions\Data\Analiz\derya\YDO-1_21.lcd

Elmt	Val.	Min	Max	Elmt	Val.	Min	Max	Elmt	Val.	Min	Max	Elmt	Val.	Min	Max	Use Adduct
H	1	5	40	O	2	2	3	S	2	0	3	Ru	2	0	0	H
C	4	0	35	F	1	0	0	Cl	1	0	2	I	3	0	0	
N	3	3	6	P	3	0	0	Br	1	0	0					

Error Margin (ppm):	5	DBE Range:	6.0 - 21.0	Electron Ions:	both
HC Ratio:	unlimited	Apply N Rule:	yes	Use MSn Info:	yes
Max Isotopes:	3	Isotope RI (%):	1.00	Isotope Res:	9000
MSn Iso RI (%):	10.00	MSn Logic Mode:	AND	Max Results:	500

Event#: 1 MS(E+) Ret. Time : 2.667 -> 2.800 Scan# : 401 -> 421

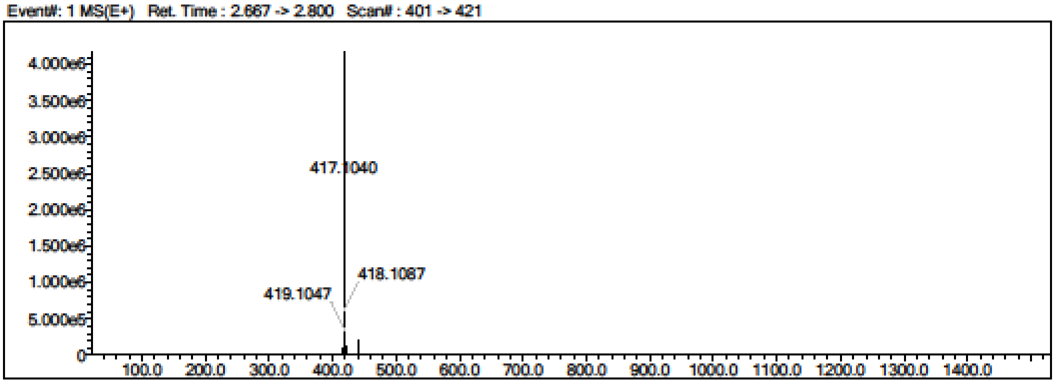

Measured region for 417.1040 m/z

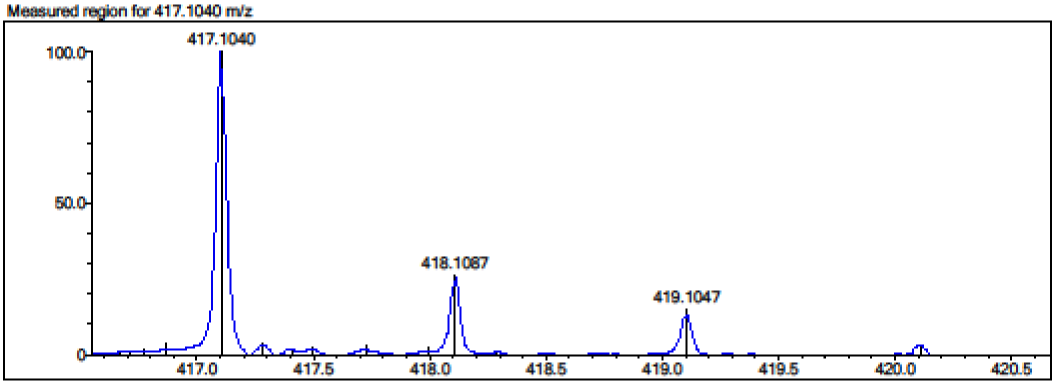

C19 H20 N4 O3 S2 [M+H]+ : Predicted region for 417.1050 m/z

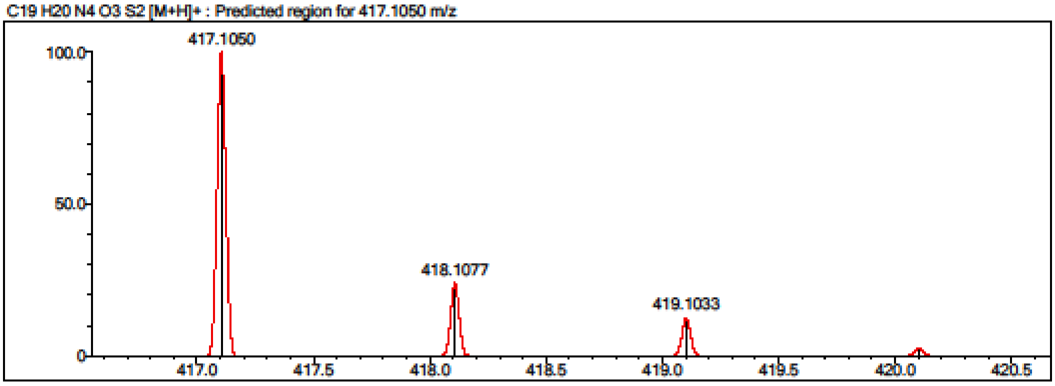

Rank	Score	Formula (M)	Ion	Meas. m/z	Pred. m/z	Df. (mDa)	Df. (ppm)	Iso	DBE
1	85.17	C19 H20 N4 O3 S2	[M+H]+	417.1040	417.1050	-1.0	-2.40	88.26	12.0

Spectra 2. HRMS spectra of compound **4a**

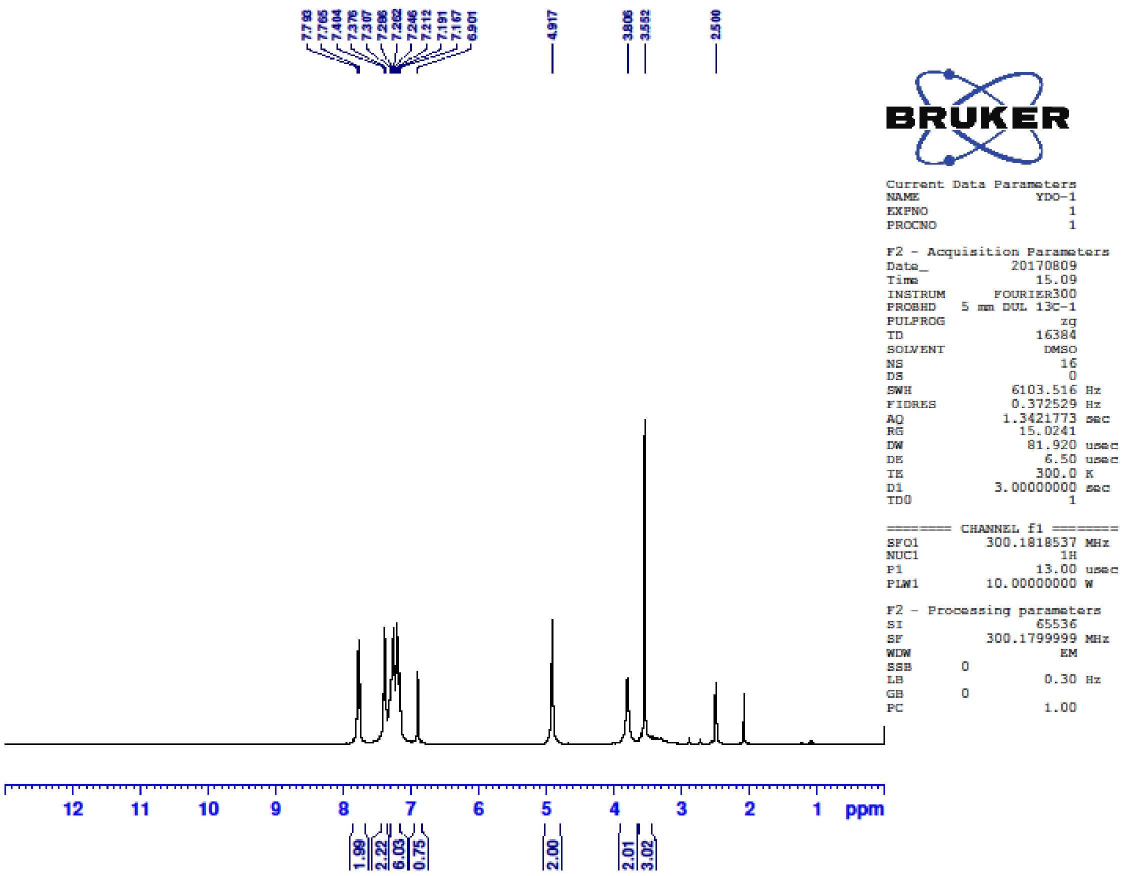

Spectra 3. ^{1}H-NMR spectra of compound **4a**

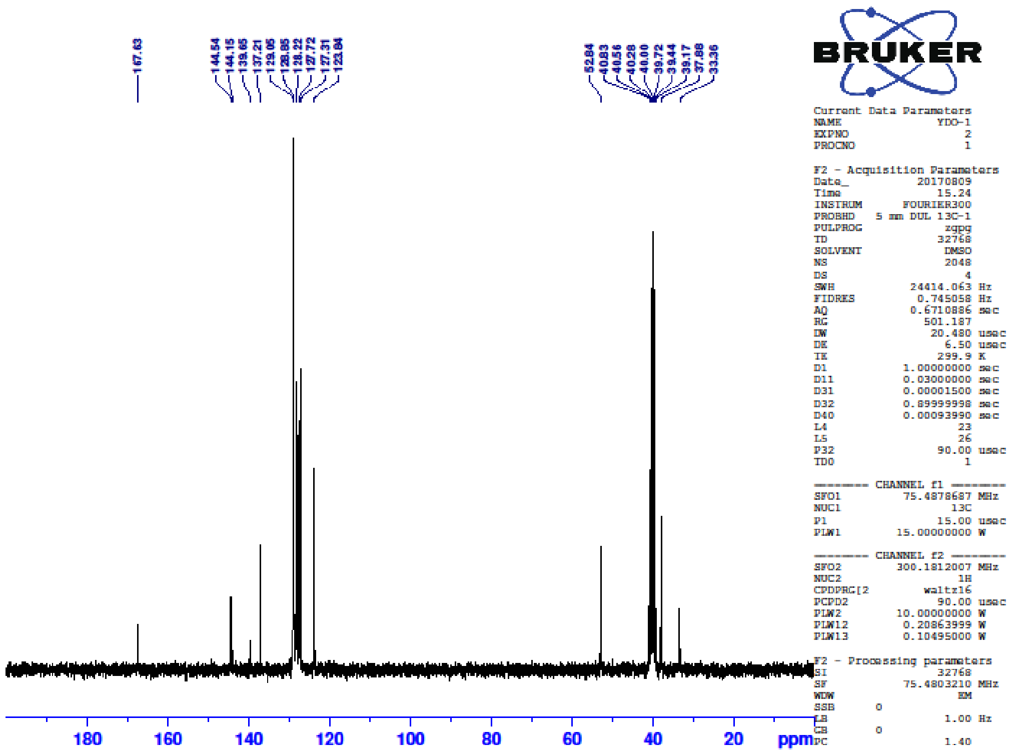

Spectra 4. ^{13}C-NMR spectra of compound **4a**

DOPNALAB

Item	Value
Acquired Date&Time	3.05.2018 13:07:24
Acquired by	System Administrator
Filename	C:\Users\dopnalab\Desktop\derya\ydo sensi\ydo-31.ispd
Spectrum name	ydo-31
Sample name	ydo-3
Sample ID	
Option	
Comment	
No. of Scans	10
Resolution	4 [cm-1]
Apodization	Happ-Genzel

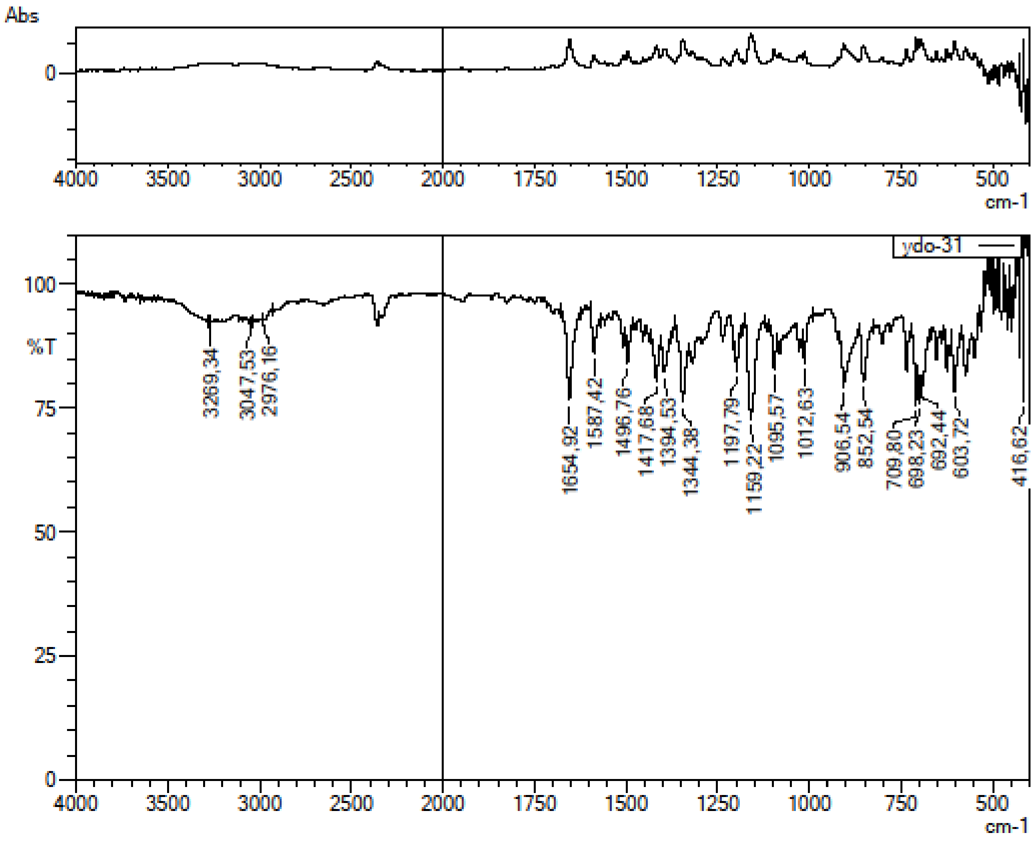

Spectra 5. IR spectra of compound **4b**

Formula Predictor Report - YDO-3_22.lcd Page 1 of 1

Data File: C:\LabSolutions\Data\Analiz\derya\YDO-3_22.lcd

Elmt	Val.	Min	Max	Elmt	Val.	Min	Max	Elmt	Val.	Min	Max	Elmt	Val.	Min	Max	Use Adduct
H	1	5	40	O	2	2	3	S	2	0	3	Ru	2	0	0	H
C	4	0	35	F	1	0	0	Cl	1	0	0	I	3	0	0	
N	3	3	6	P	3	0	0	Br	1	0	0					

Error Margin (ppm):	5	DBE Range:	6.0 - 21.0	Electron Ions:	both
HC Ratio:	unlimited	Apply N Rule:	yes	Use MSn Info:	yes
Max Isotopes:	3	Isotope RI (%):	1.00	Isotope Res:	9000
MSn Iso RI (%):	10.00	MSn Logic Mode:	AND	Max Results:	500

Event#: 1 MS(E+) Ret. Time : 4.560 -> 4.667 Scan# : 685 -> 701

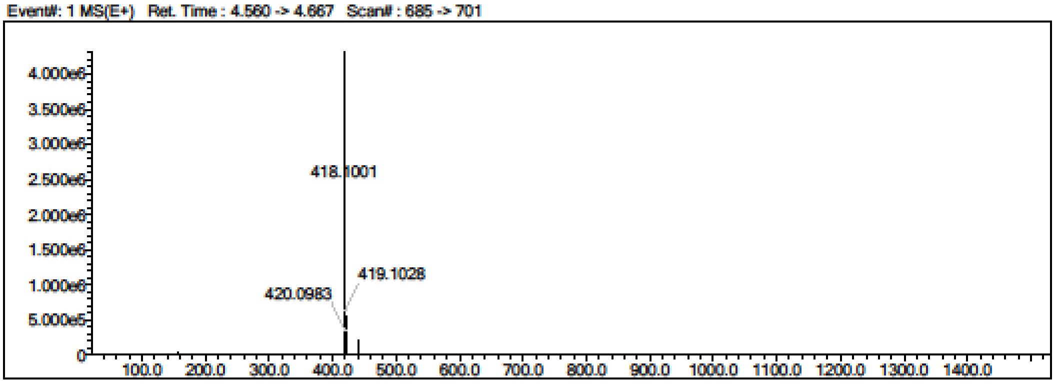

Measured region for 418.1001 m/z

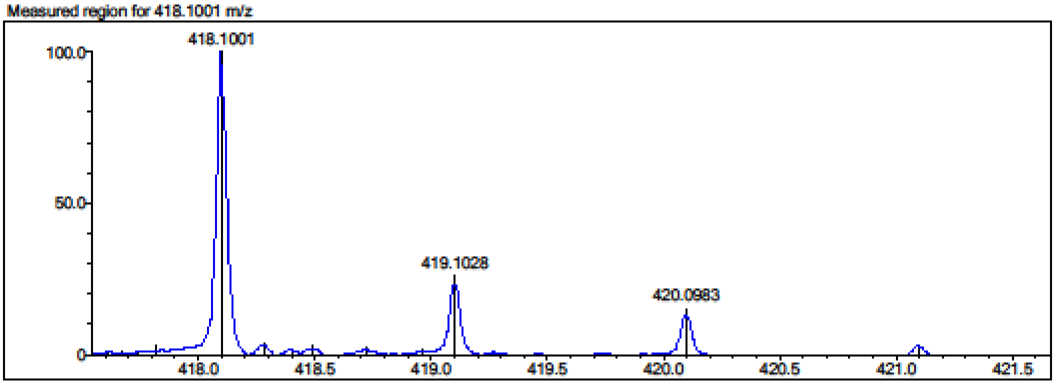

C18 H19 N5 O3 S2 [M+H]+ : Predicted region for 418.1002 m/z

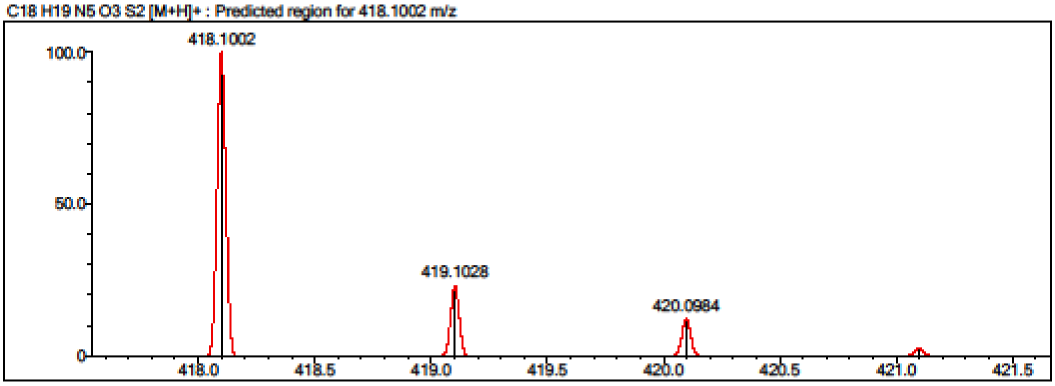

Rank	Score	Formula (M)	Ion	Meas. m/z	Pred. m/z	Df. (mDa)	Df. (ppm)	Iso	DBE
1	90.03	C18 H19 N5 O3 S2	[M+H]+	418.1001	418.1002	-0.1	-0.24	90.03	12.0

Spectra 6. HRMS spectra of compound **4b**

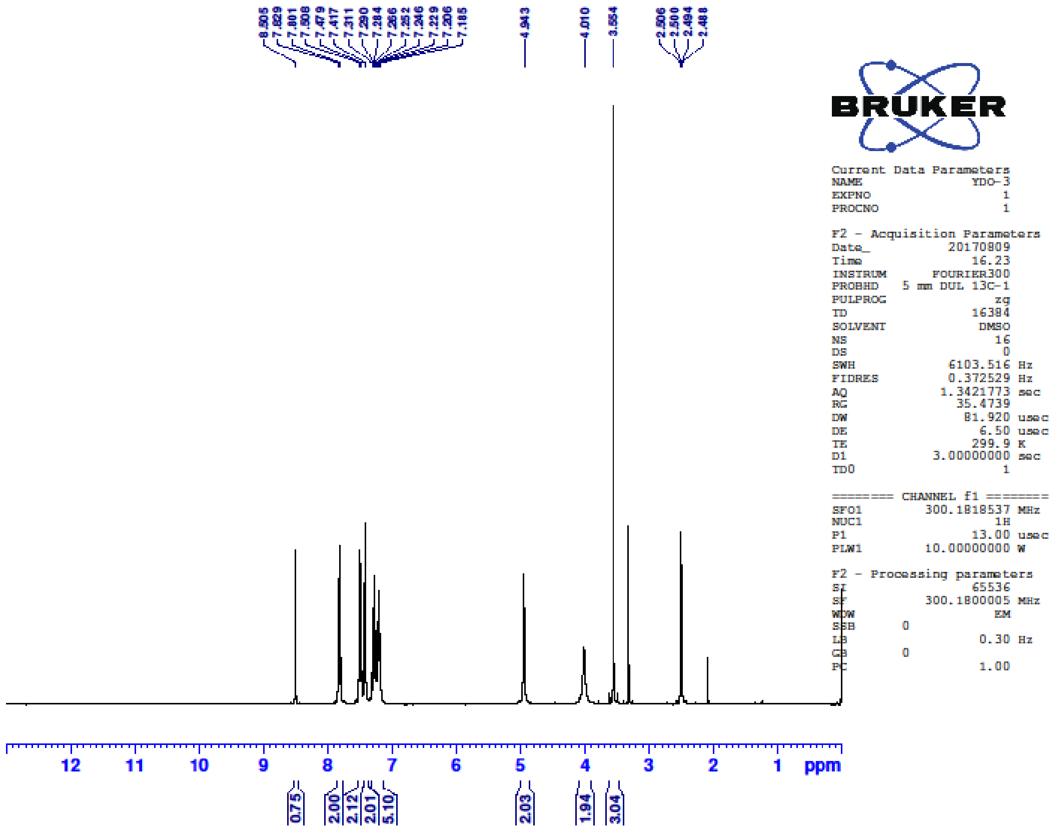

Spectra 7. ^{1}H-NMR spectra of compound **4b**

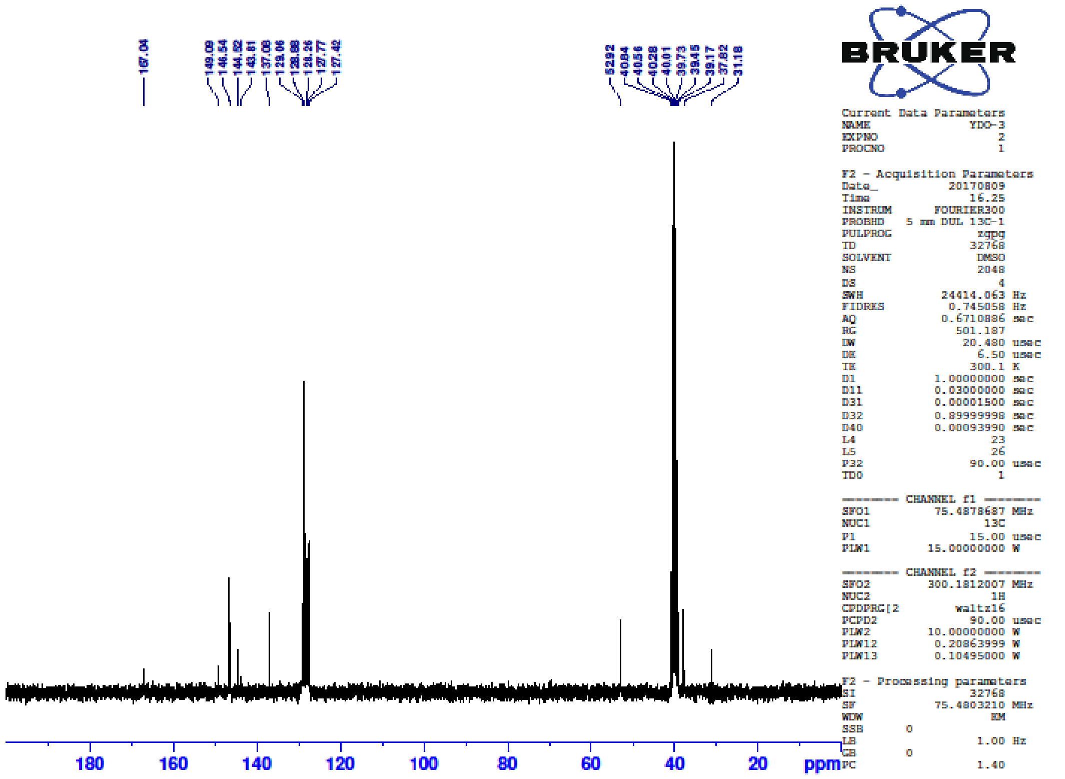

Spectra 8. ^{13}C-NMR spectra of compound **4b**

DOPNALAB

Item	Value
Acquired Date&Time	3.05.2018 13:12:40
Acquired by	System Administrator
Filename	C:\Users\dopnalab\Desktop\derya\ydo sensi\ydo-51.ispd
Spectrum name	ydo-51
Sample name	ydo-5
Sample ID	
Option	
Comment	
No. of Scans	10
Resolution	4 [cm-1]
Apodization	Happ-Genzel

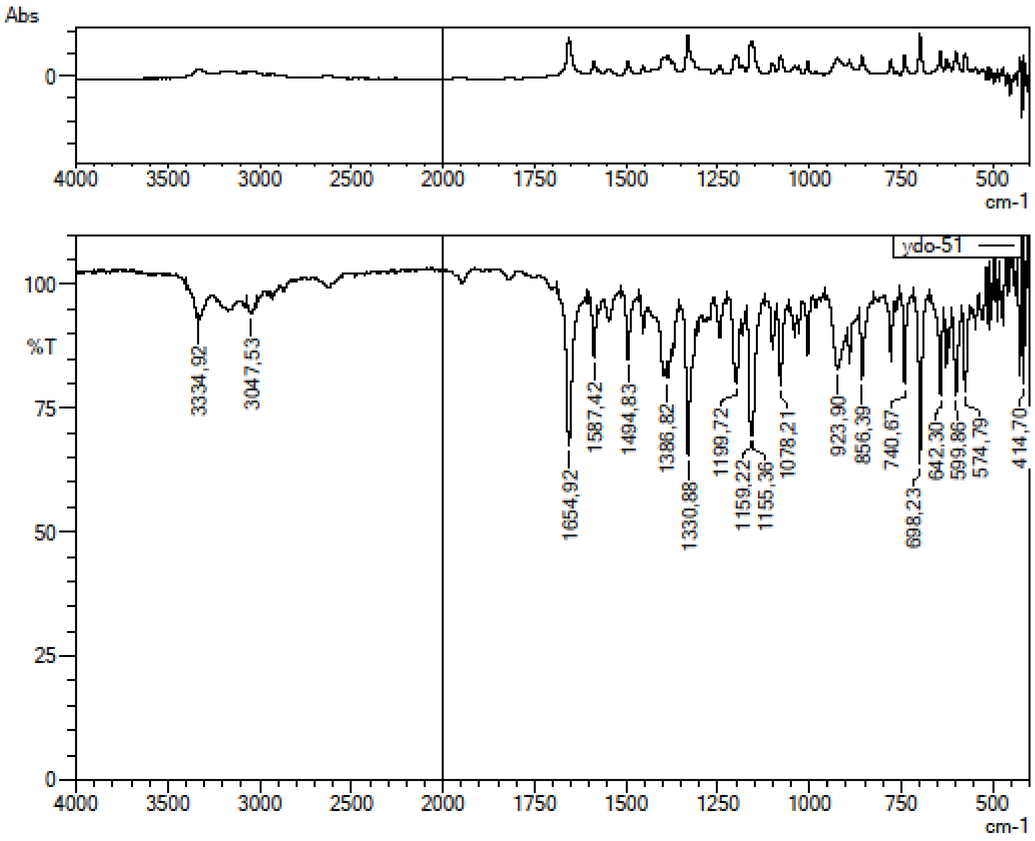

Spectra 9. IR spectra of compound **4c**

Formula Predictor Report - YD0-5_23.lcd

Data File: C:\LabSolutions\Data\Analiz\derya\YD0-5_23.lcd

Elmt	Val.	Min	Max	Elmt	Val.	Min	Max	Elmt	Val.	Min	Max	Elmt	Val.	Min	Max	Use Adduct
H	1	5	40	O	2	2	3	S	2	0	3	Ru	2	0	0	H
C	4	0	35	F	1	0	0	Cl	1	0	0	I	3	0	0	
N	3	3	6	P	3	0	0	Br	1	0	0					

Error Margin (ppm): 5
HC Ratio: unlimited
Max Isotopes: 3
MSn Iso RI (%): 10.00

DBE Range: 6.0 - 21.0
Apply N Rule: yes
Isotope RI (%): 1.00
MSn Logic Mode: AND

Electron Ions: both
Use MSn Info: yes
Isotope Res: 9000
Max Results: 500

Event#: 1 MS(E+) Ret. Time : 5.333 -> 5.453 Scan# : 801 -> 819

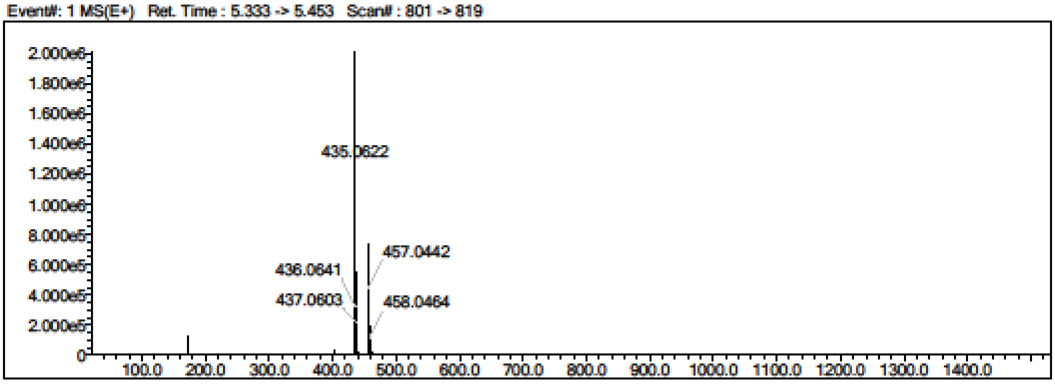

Measured region for 435.0622 m/z

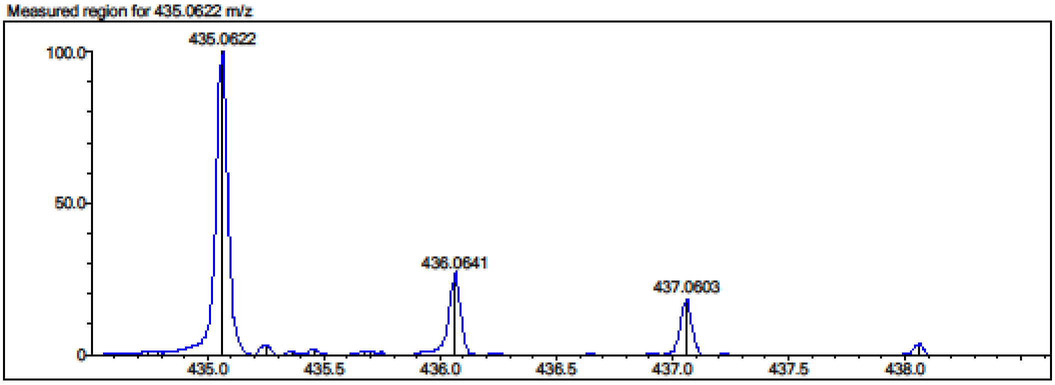

C18 H18 N4 O3 S3 [M+H]+ : Predicted region for 435.0614 m/z

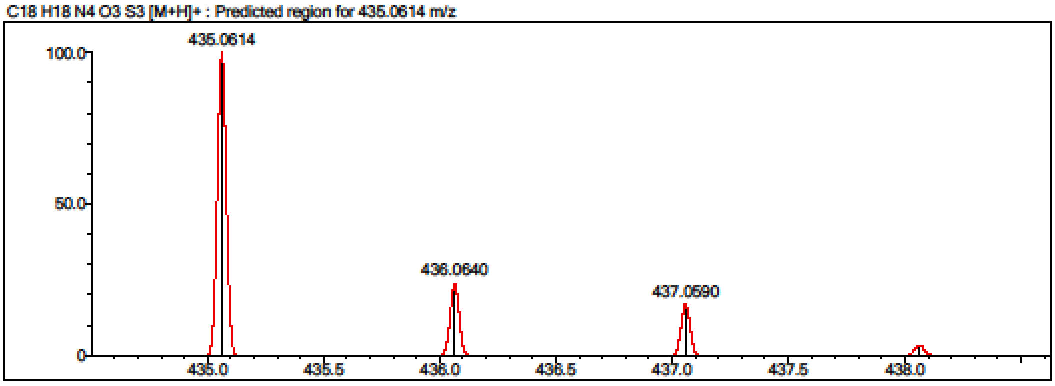

Rank	Score	Formula (M)	Ion	Meas. m/z	Pred. m/z	Df. (mDa)	Df. (ppm)	Iso	DBE
1	97.90	C18 H18 N4 O3 S3	[M+H]+	435.0622	435.0614	0.8	1.84	100.00	12.0

Spectra 10. HRMS spectra of compound **4c**

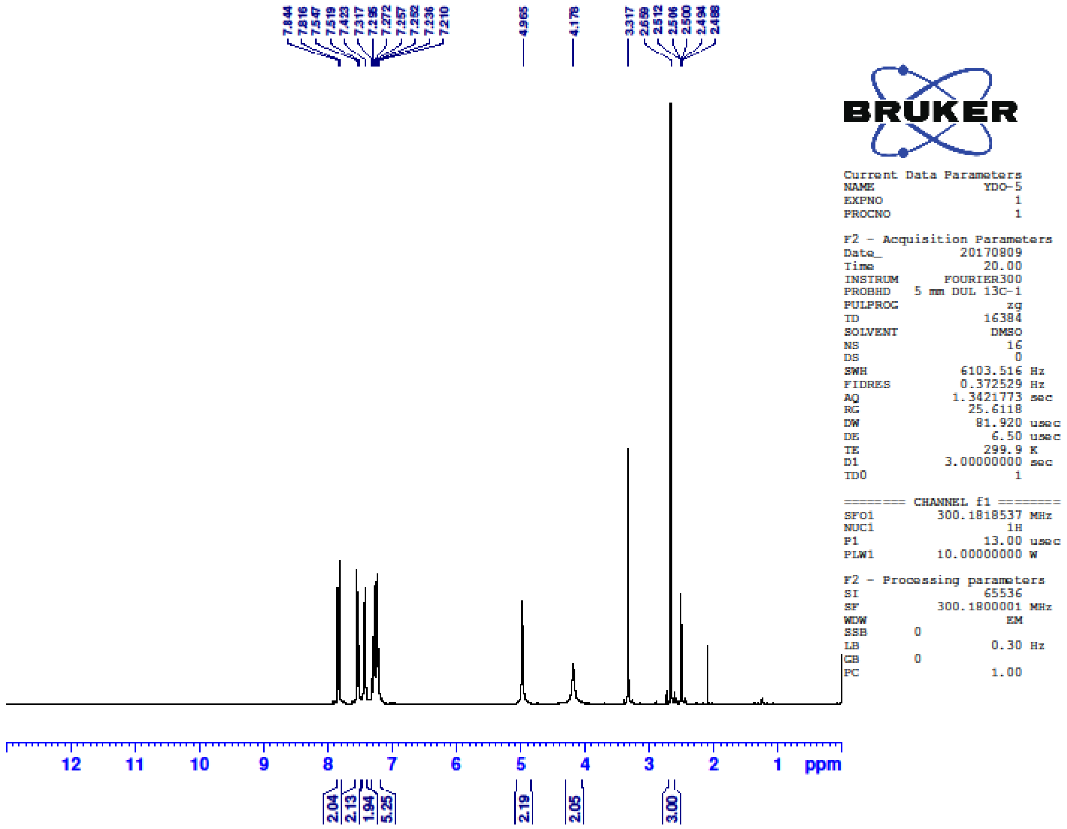

Spectra 11. ^{1}H-NMR spectra of compound **4c**

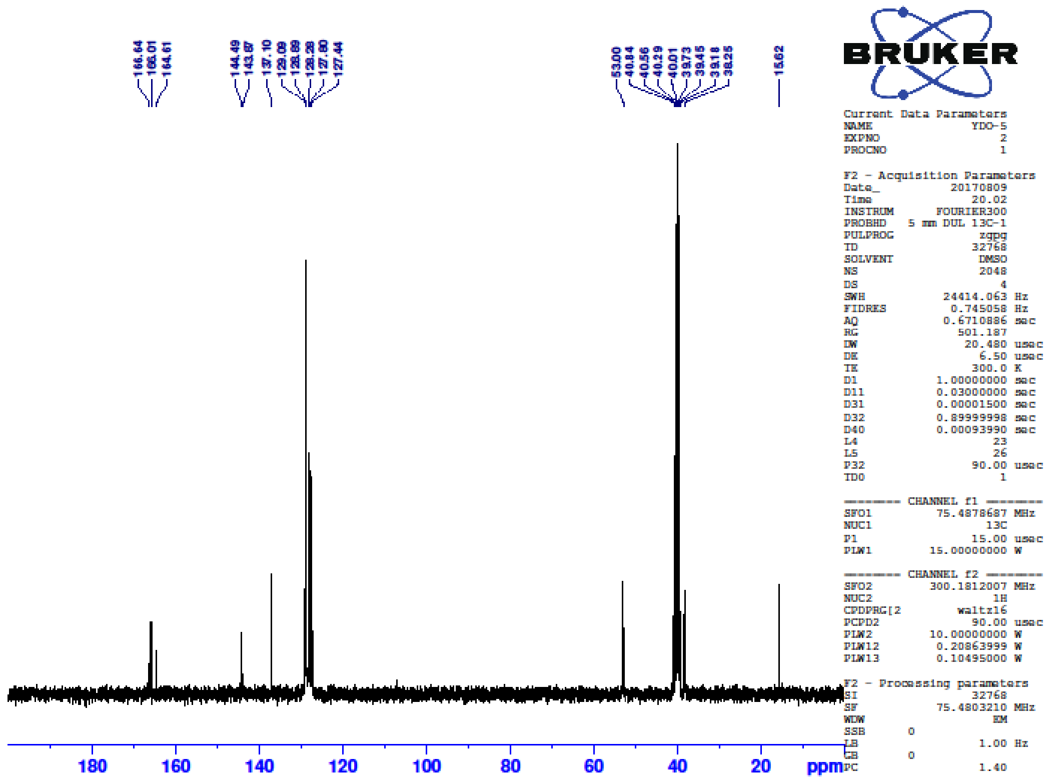

Spectra 12. ^{13}C-NMR spectra of compound **4c**

DOPNALAB

Item	Value
Acquired Date&Time	3.05.2018 13:16:07
Acquired by	System Administrator
Filename	C:\Users\dopnalab\Desktop\derya\ydo sensi\ydo-61.ispd
Spectrum name	ydo-61
Sample name	ydo-6
Sample ID	
Option	
Comment	
No. of Scans	10
Resolution	4 [cm-1]
Apodization	Happ-Genzel

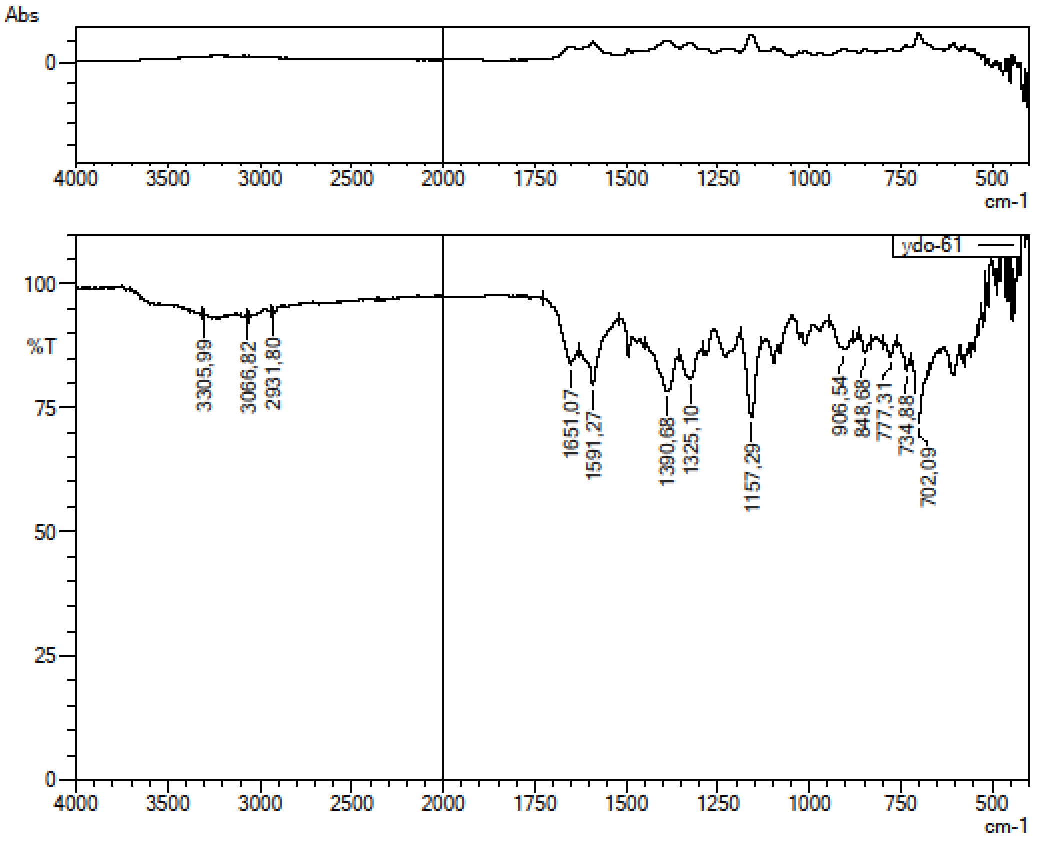

Spectra 13. IR spectra of compound **4d**

Formula Predictor Report - YD0-6_24.lcd Page 1 of 1

Data File: C:\LabSolutions\Data\Analiz\derya\YD0-6_24.lcd

Elmt	Val.	Min	Max	Elmt	Val.	Min	Max	Elmt	Val.	Min	Max	Elmt	Val.	Min	Max	Use Adduct
H	1	5	40	O	2	2	3	S	2	0	3	Ru	2	0	0	H
C	4	0	35	F	1	0	0	Cl	1	0	0	I	3	0	0	
N	3	3	6	P	3	0	0	Br	1	0	0					

Error Margin (ppm):	5	DBE Range:	6.0 - 21.0	Electron Ions:	both
HC Ratio:	unlimited	Apply N Rule:	yes	Use MSn Info:	yes
Max Isotopes:	3	Isotope RI (%):	1.00	Isotope Res:	9000
MSn Iso RI (%):	10.00	MSn Logic Mode:	AND	Max Results:	500

Event#: 1 MS(E+) Ret. Time : 5.200 -> 5.400 Scan# : 781 -> 811

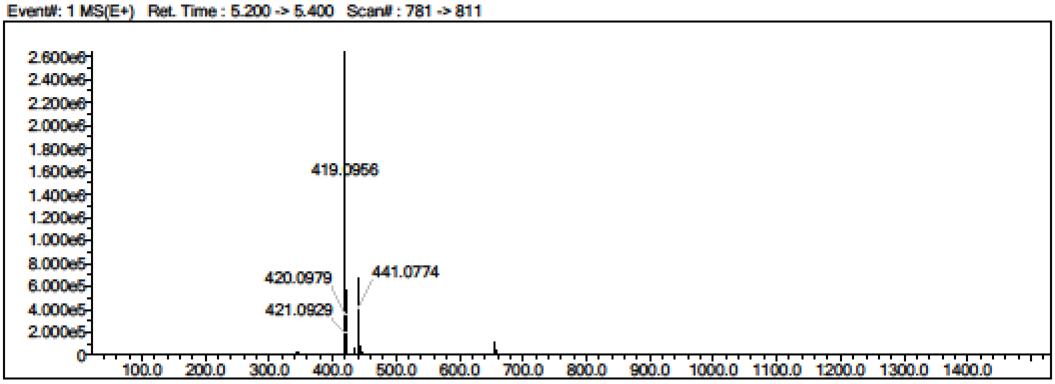

Measured region for 419.0956 m/z

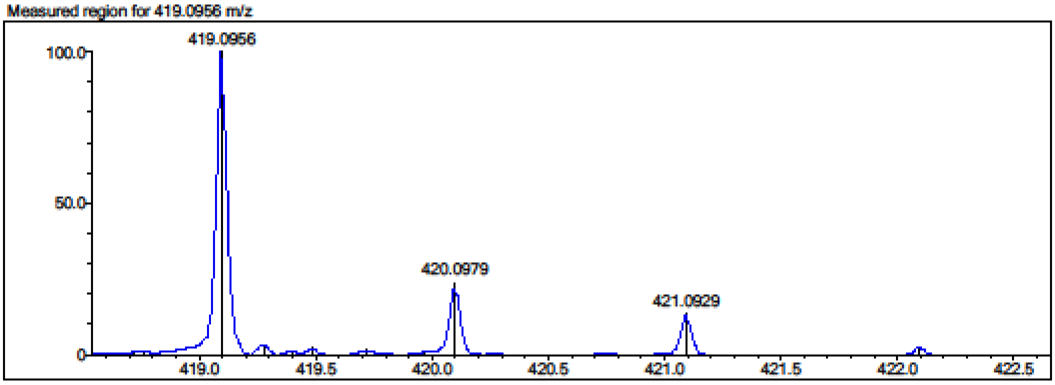

C17 H18 N6 O3 S2 [M+H]+ : Predicted region for 419.0955 m/z

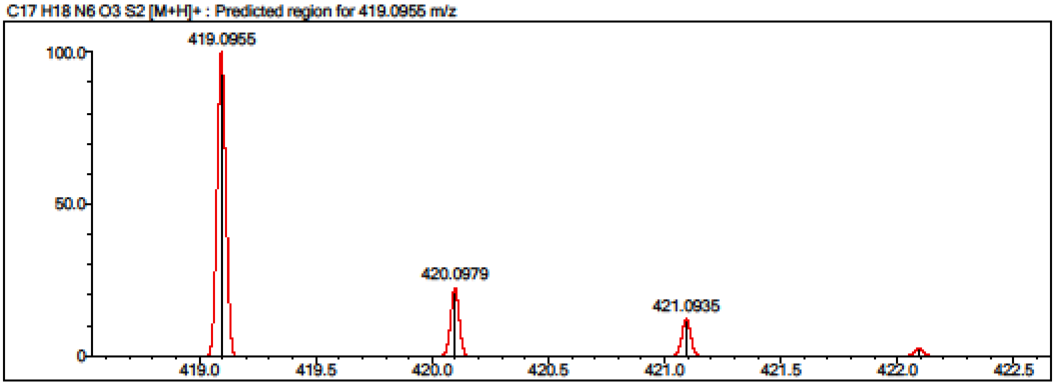

Rank	Score	Formula (M)	Ion	Meas. m/z	Pred. m/z	Df. (mDa)	Df. (ppm)	Iso	DBE
1	95.49	C17 H18 N6 O3 S2	[M+H]+	419.0956	419.0955	0.1	0.24	95.49	12.0

Spectra 14. HRMS spectra of compound **4d**

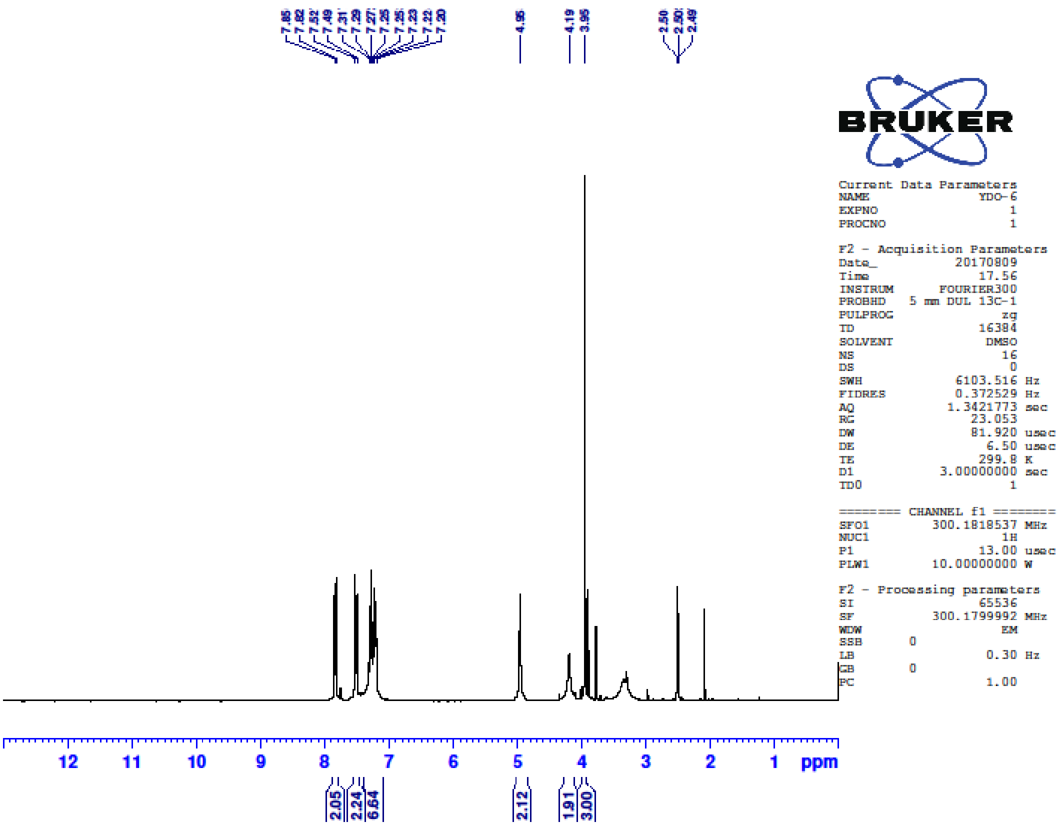

Spectra 15. ^{1}H-NMR spectra of compound **4d**

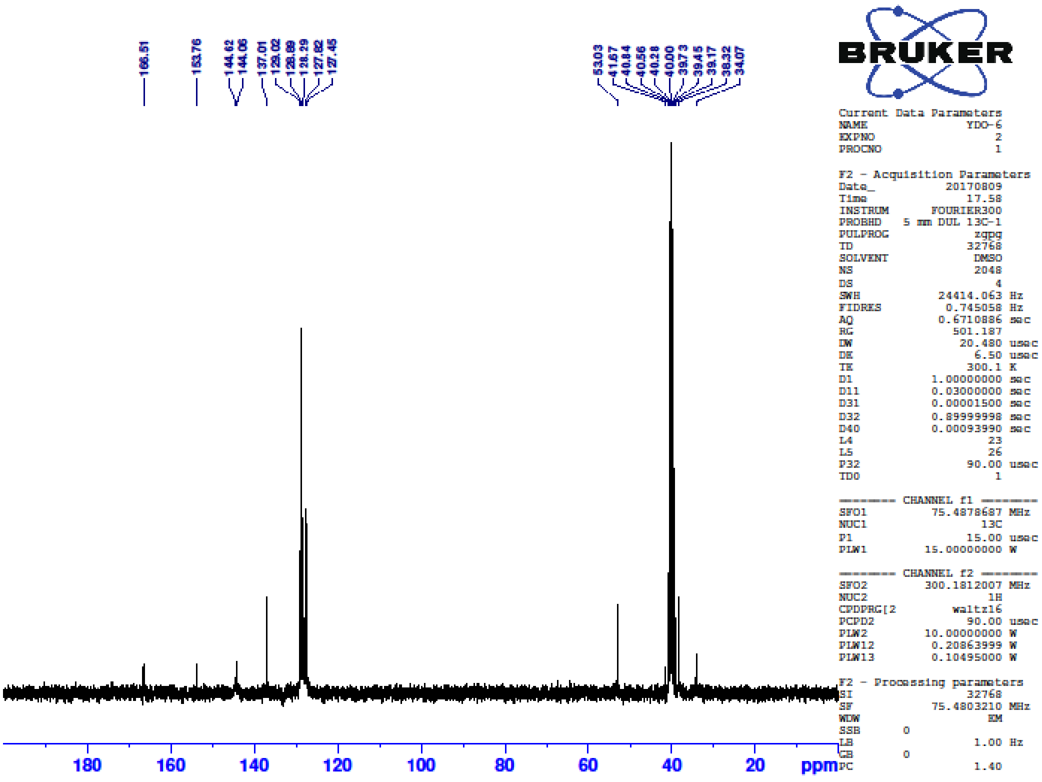

Spectra 16. ^{13}C-NMR spectra of compound **4d**

DOPNALAB

Item	Value
Acquired Date&Time	3.05.2018 13:18:36
Acquired by	System Administrator
Filename	C:\Users\dopnalab\Desktop\derya\ydo sensi\ydo-71.ispd
Spectrum name	ydo-71
Sample name	ydo-7
Sample ID	
Option	
Comment	
No. of Scans	10
Resolution	4 [cm-1]
Apodization	Happ-Genzel

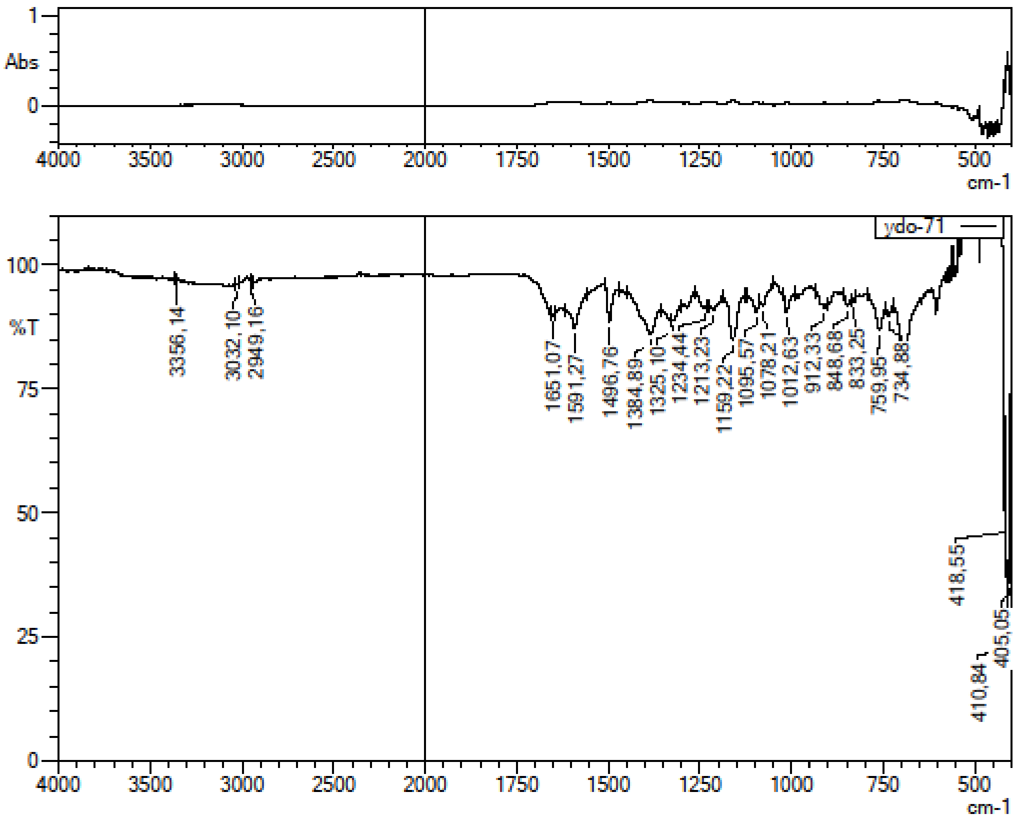

Spectra 17. IR spectra of compound **4e**

Data File: C:\LabSolutions\Data\Analiz\derya\YD0-7_25.lcd

Elmt	Val.	Min	Max	Elmt	Val.	Min	Max	Elmt	Val.	Min	Max	Elmt	Val.	Min	Max	Use Adduct
H	1	5	40	O	2	2	3	S	2	0	3	Ru	2	0	0	H
C	4	0	35	F	1	0	0	Cl	1	0	0	I	3	0	0	
N	3	3	6	P	3	0	0	Br	1	0	0					

Error Margin (ppm):	5	DBE Range:	6.0 - 20.0	Electron Ions:	both
HC Ratio:	unlimited	Apply N Rule:	yes	Use MSn Info:	yes
Max Isotopes:	3	Isotope RI (%):	1.00	Isotope Res:	9000
MSn Iso RI (%):	10.00	MSn Logic Mode:	AND	Max Results:	500

Event#: 1 MS(E+) Ret. Time : 6.240 -> 6.347 Scan# : 937 -> 953

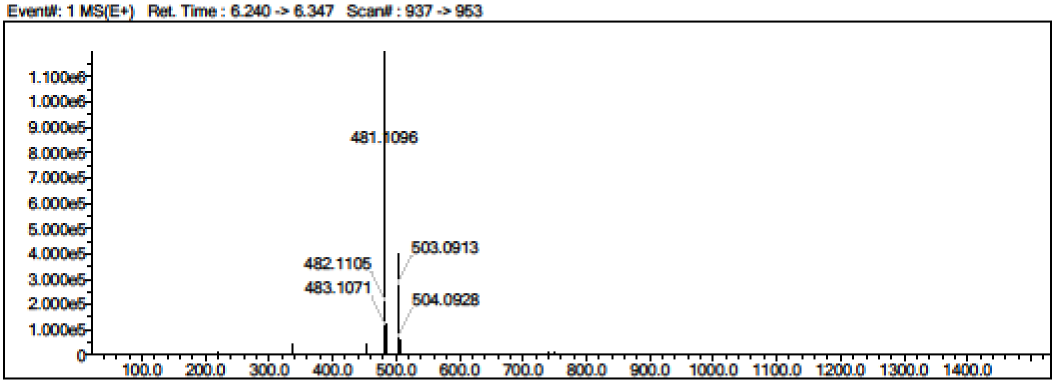

Measured region for 481.1096 m/z

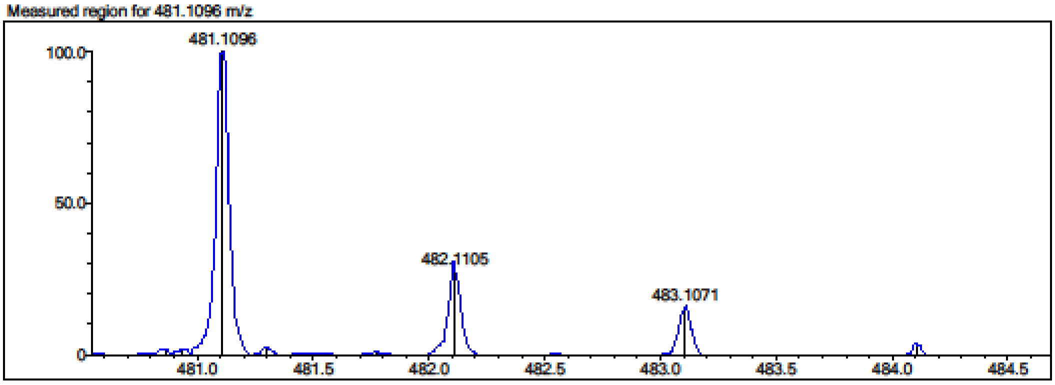

C22 H20 N6 O3 S2 [M+H]+ : Predicted region for 481.1111 m/z

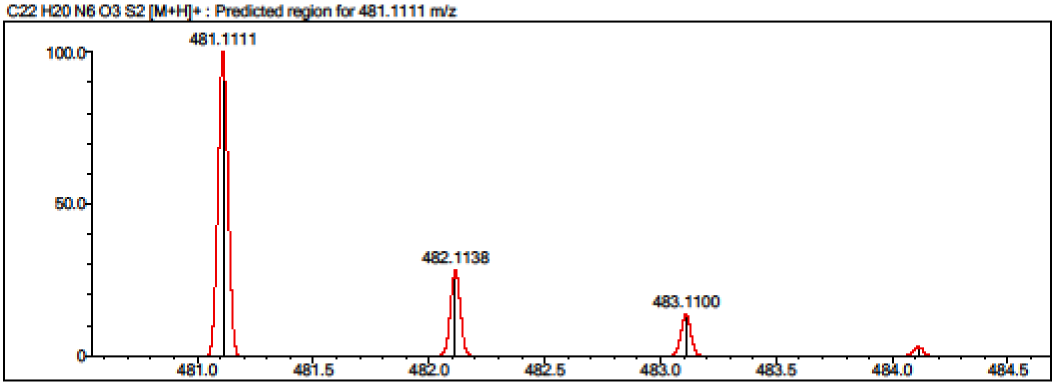

Rank	Score	Formula (M)	Ion	Meas. m/z	Pred. m/z	Df. (mDa)	Df. (ppm)	Iso	DBE
1	87.52	C22 H20 N6 O3 S2	[M+H]+	481.1096	481.1111	-1.5	-3.12	92.42	16.0

Spectra 18. HRMS spectra of compound **4e**

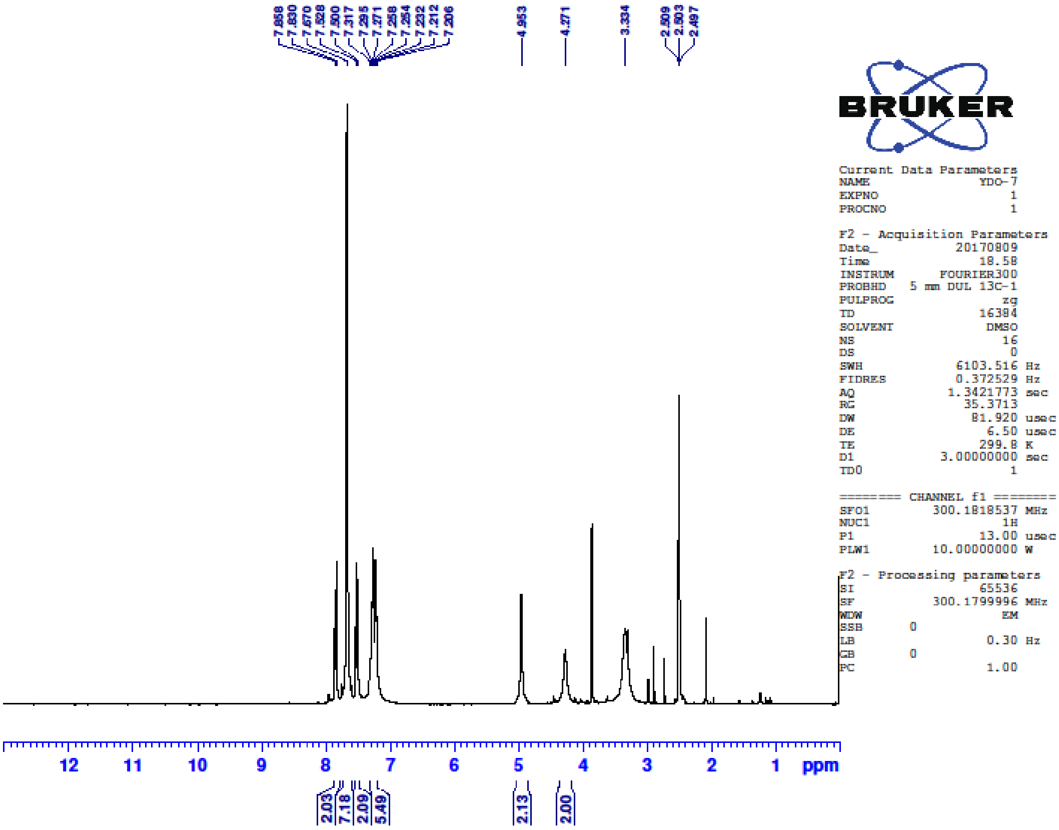

Spectra 19. ^{1}H-NMR spectra of compound **4e**

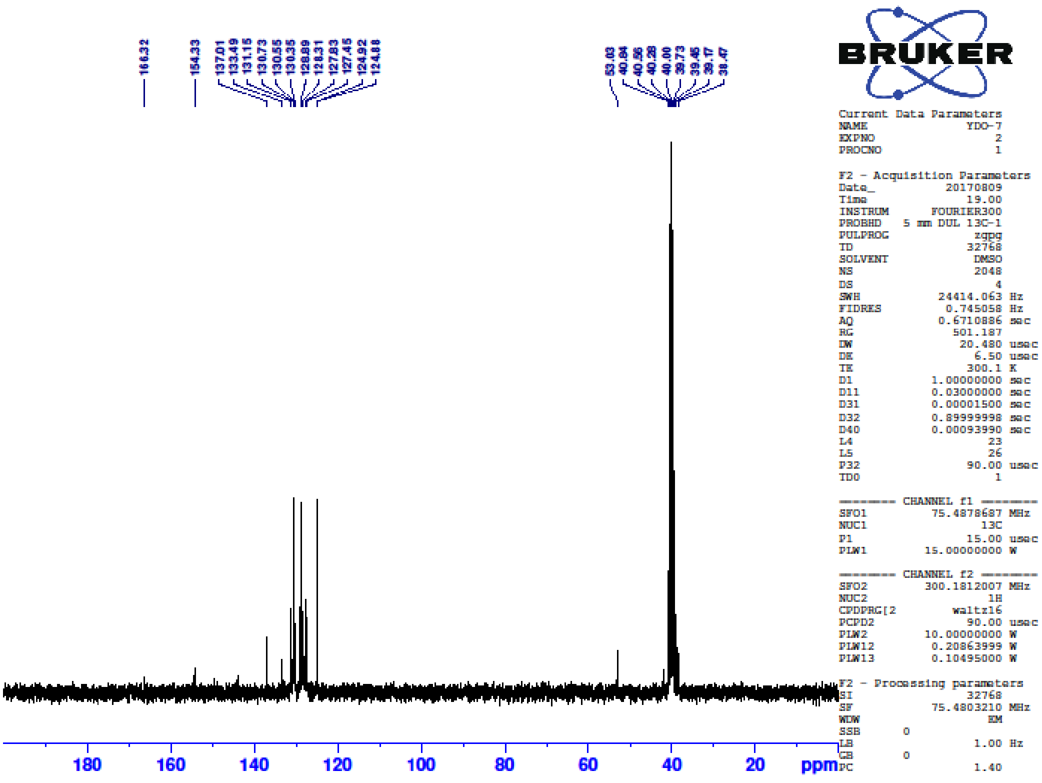

Spectra 20. ^{13}C-NMR spectra of compound **4e**

DOPNALAB

	Item	Value
	Acquired Date&Time	3.05.2018 13:21:44
	Acquired by	System Administrator
	Filename	C:\Users\dopnalab\Desktop\derya\ydo sensi\ydo-81.ispd
	Spectrum name	ydo-81
	Sample name	ydo-8
	Sample ID	
	Option	
	Comment	
	No. of Scans	10
	Resolution	4 [cm-1]
	Apodization	Happ-Genzel

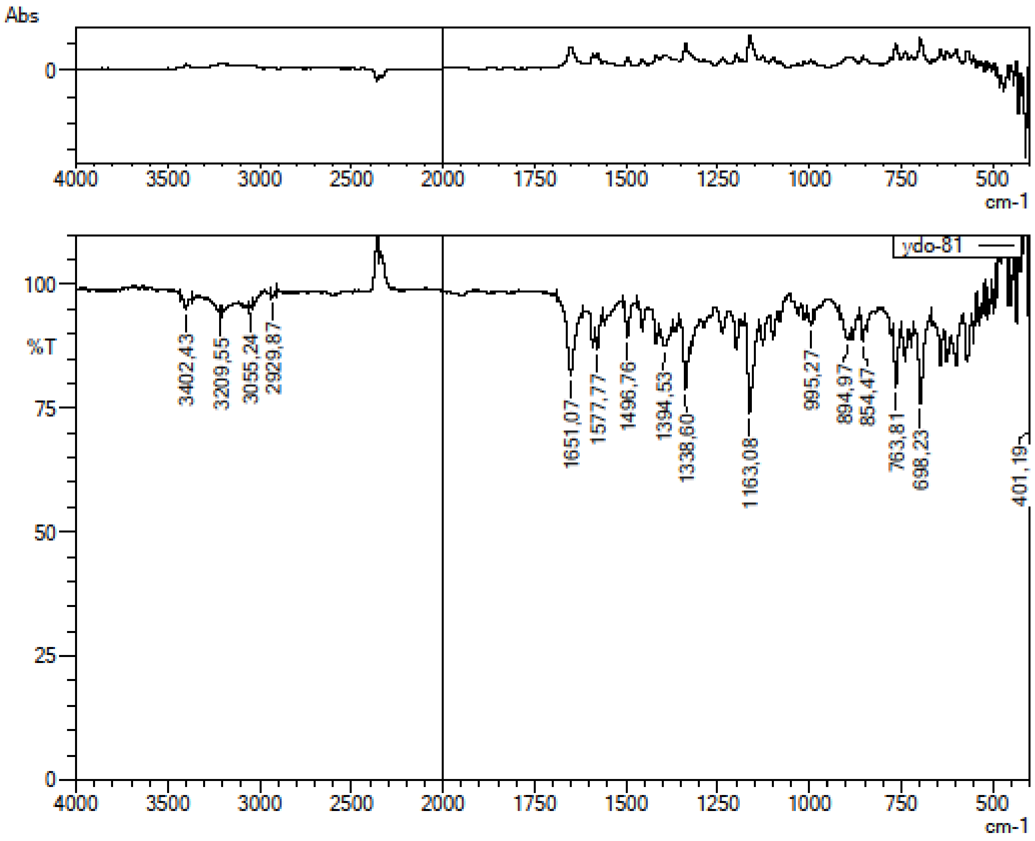

Spectra 21. IR spectra of compound **4f**

Data File: C:\LabSolutions\Data\Analiz\derya\YD0-8_26.lcd

Elmt	Val.	Min	Max	Elmt	Val.	Min	Max	Elmt	Val.	Min	Max	Elmt	Val.	Min	Max	Use Adduct
H	1	5	40	O	2	2	3	S	2	0	3	Ru	2	0	0	H
C	4	0	35	F	1	0	0	Cl	1	0	0	I	3	0	0	
N	3	3	6	P	3	0	0	Br	1	0	0					

Error Margin (ppm): 5
HC Ratio: unlimited
Max Isotopes: 3
MSn Iso RI (%): 10.00

DBE Range: 6.0 - 20.0
Apply N Rule: yes
Isotope RI (%): 1.00
MSn Logic Mode: AND

Electron Ions: both
Use MSn Info: yes
Isotope Res: 9000
Max Results: 500

Event#: 1 MS(E+) Ret. Time : 5.680 -> 5.787 Scan# : 853 -> 869

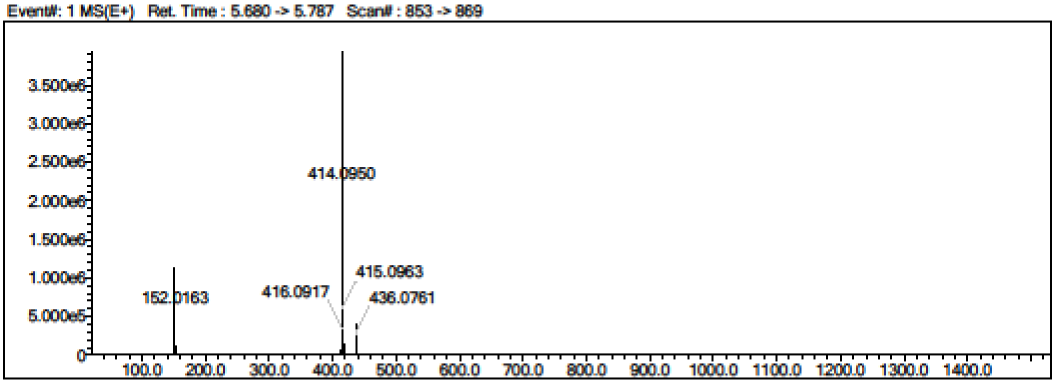

Measured region for 414.0950 m/z

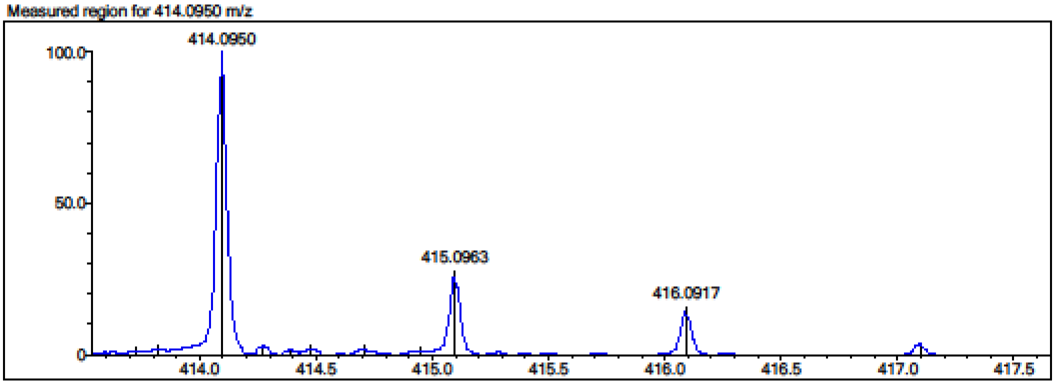

C20 H19 N3 O3 S2 [M+H]+ : Predicted region for 414.0941 m/z

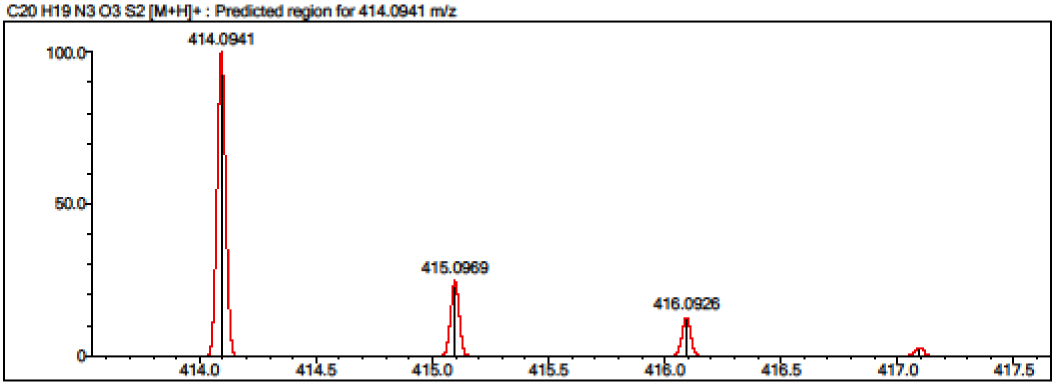

Rank	Score	Formula (M)	Ion	Meas. m/z	Pred. m/z	Df. (mDa)	Df. (ppm)	Iso	DBE
1	88.22	C20 H19 N3 O3 S2	[M+H]+	414.0950	414.0941	0.9	2.17	90.88	13.0

Spectra 22. HRMS spectra of compound **4f**

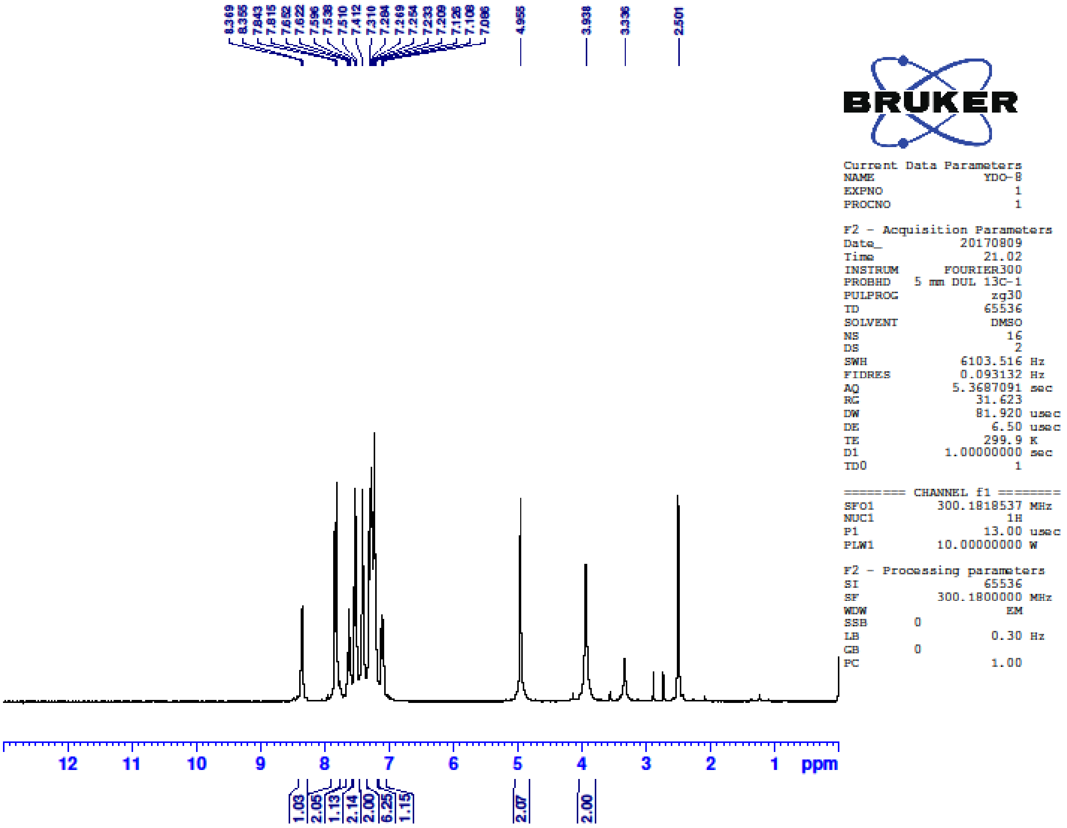

Spectra 23. ^{1}H-NMR spectra of compound **4f**

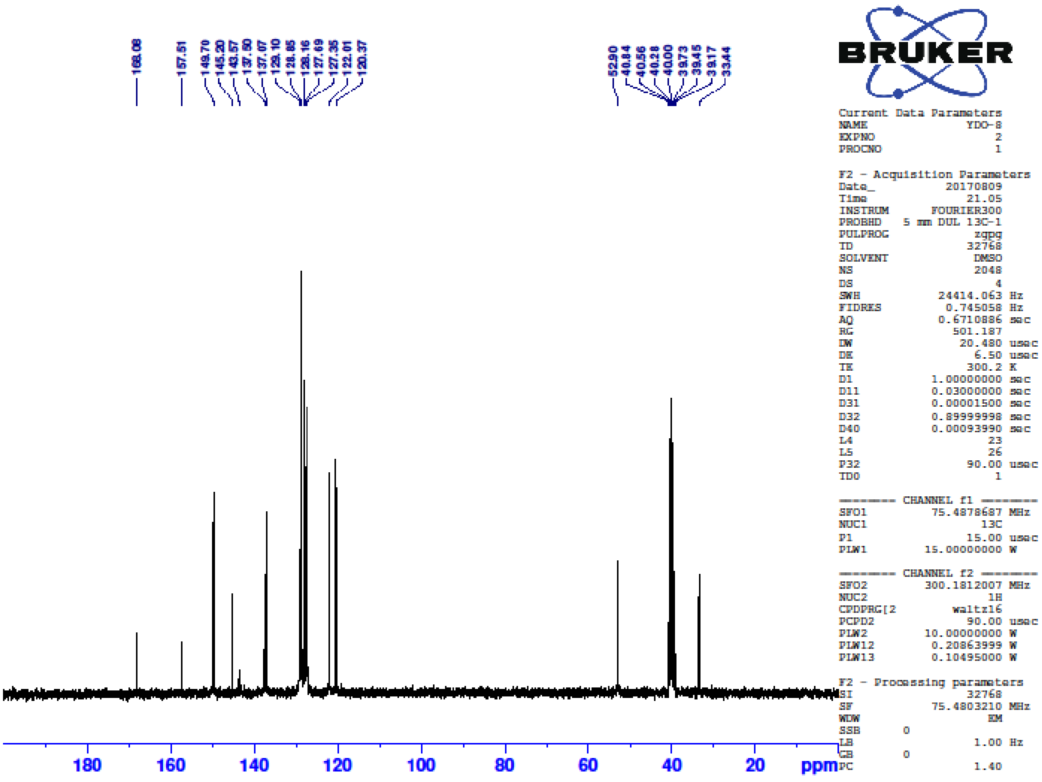

Spectra 24. ^{13}C-NMR spectra of compound **4f**

DOPNALAB

	Item	Value
	Acquired Date&Time	3.05.2018 13:25:25
	Acquired by	System Administrator
	Filename	C:\Users\dopnalab\Desktop\derya\ydo sensi\ydo-101.ispd
	Spectrum name	ydo-101
	Sample name	ydo-10
	Sample ID	
	Option	
	Comment	
	No. of Scans	10
	Resolution	4 [cm-1]
	Apodization	Happ-Genzel

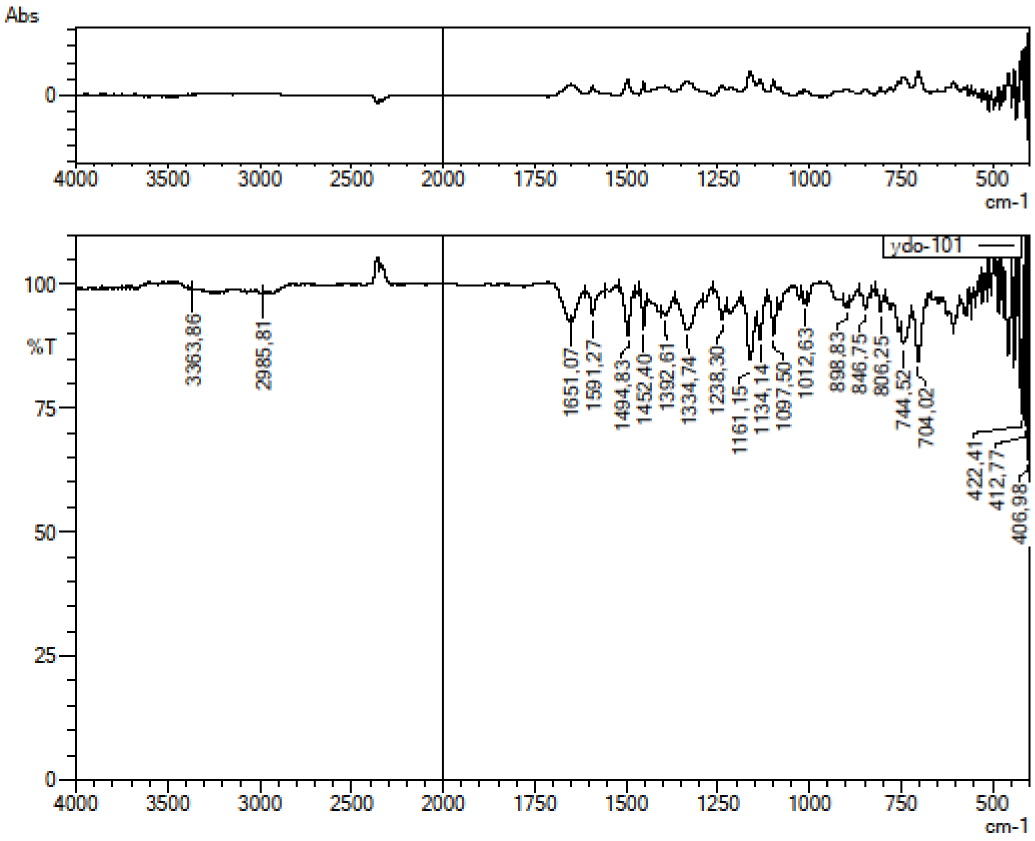

Spectra 25. IR spectra of compound **4g**

Data File: C:\LabSolutions\Data\Analiz\derya\YD0-10_28.lcd

Elmt	Val.	Min	Max	Elmt	Val.	Min	Max	Elmt	Val.	Min	Max	Elmt	Val.	Min	Max	Use Adduct
H	1	5	40	O	2	2	4	S	2	0	3	Ru	2	0	0	H
C	4	0	35	F	1	0	0	Cl	1	0	0	I	3	0	0	
N	3	3	6	P	3	0	0	Br	1	0	0					

Error Margin (ppm): 5
HC Ratio: unlimited
Max Isotopes: 3
MSn Iso RI (%): 10.00

DBE Range: 6.0 - 20.0
Apply N Rule: yes
Isotope RI (%): 1.00
MSn Logic Mode: AND

Electron Ions: both
Use MSn Info: yes
Isotope Res: 9000
Max Results: 500

Event#: 1 MS(E+) Ret. Time : 6.320 -> 6.413 Scan# : 949 -> 963

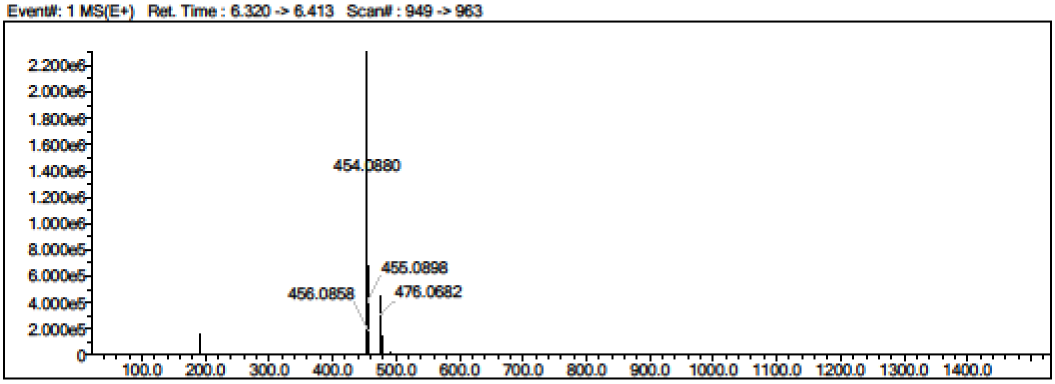

Measured region for 454.0880 m/z

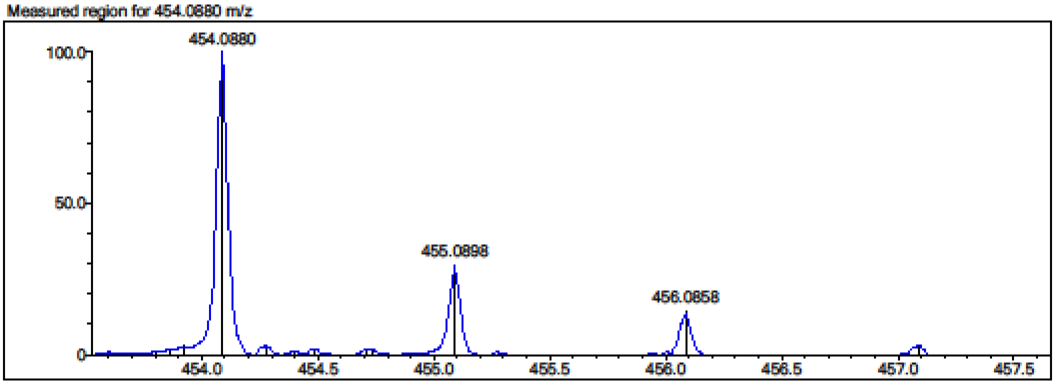

C22 H19 N3 O4 S2 [M+H]+ : Predicted region for 454.0890 m/z

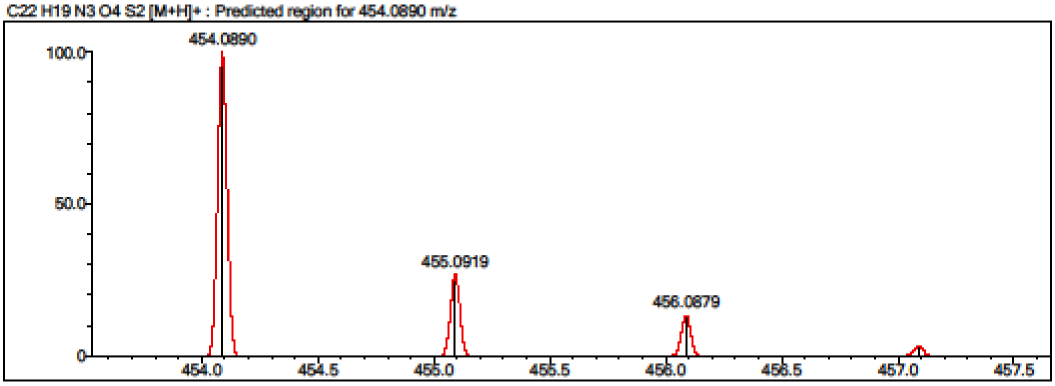

Rank	Score	Formula (M)	Ion	Meas. m/z	Pred. m/z	Df. (mDa)	Df. (ppm)	Iso	DBE
1	96.53	C22 H19 N3 O4 S2	[M+H]+	454.0880	454.0890	-1.0	-2.20	99.51	15.0

Spectra 26. HRMS spectra of compound **4g**

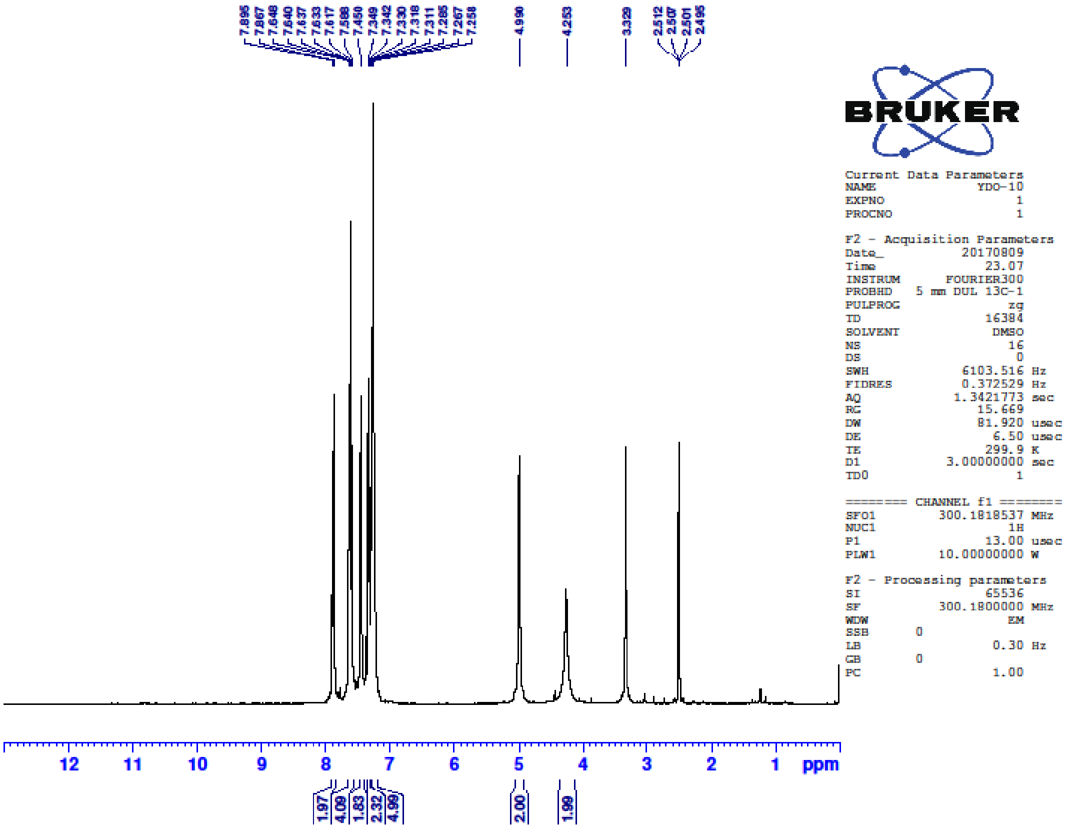

Spectra 27. ^{1}H-NMR spectra of compound **4g**

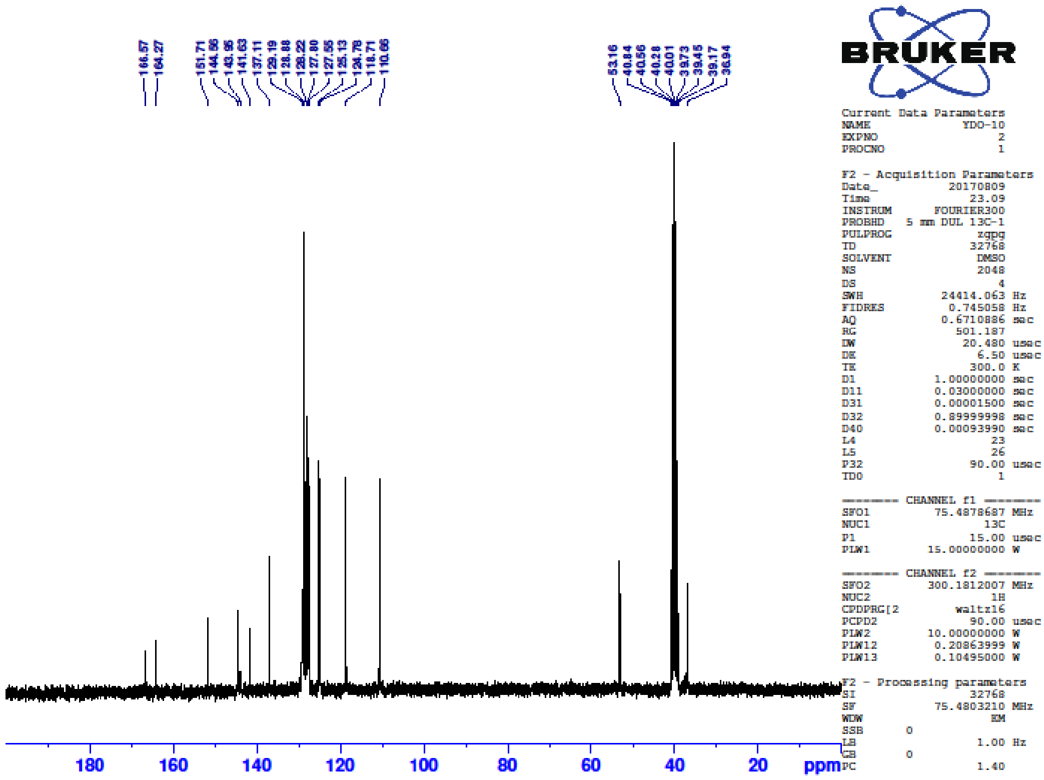

Spectra 28. ^{13}C-NMR spectra of compound **4g**

DOPNALAB

Item	Value
Acquired Date&Time	3.05.2018 13:29:13
Acquired by	System Administrator
Filename	C:\Users\dopnalab\Desktop\derya\ydo sensi\ydo-111.ispd
Spectrum name	ydo-111
Sample name	ydo-11
Sample ID	
Option	
Comment	
No. of Scans	10
Resolution	4 [cm-1]
Apodization	Happ-Genzel

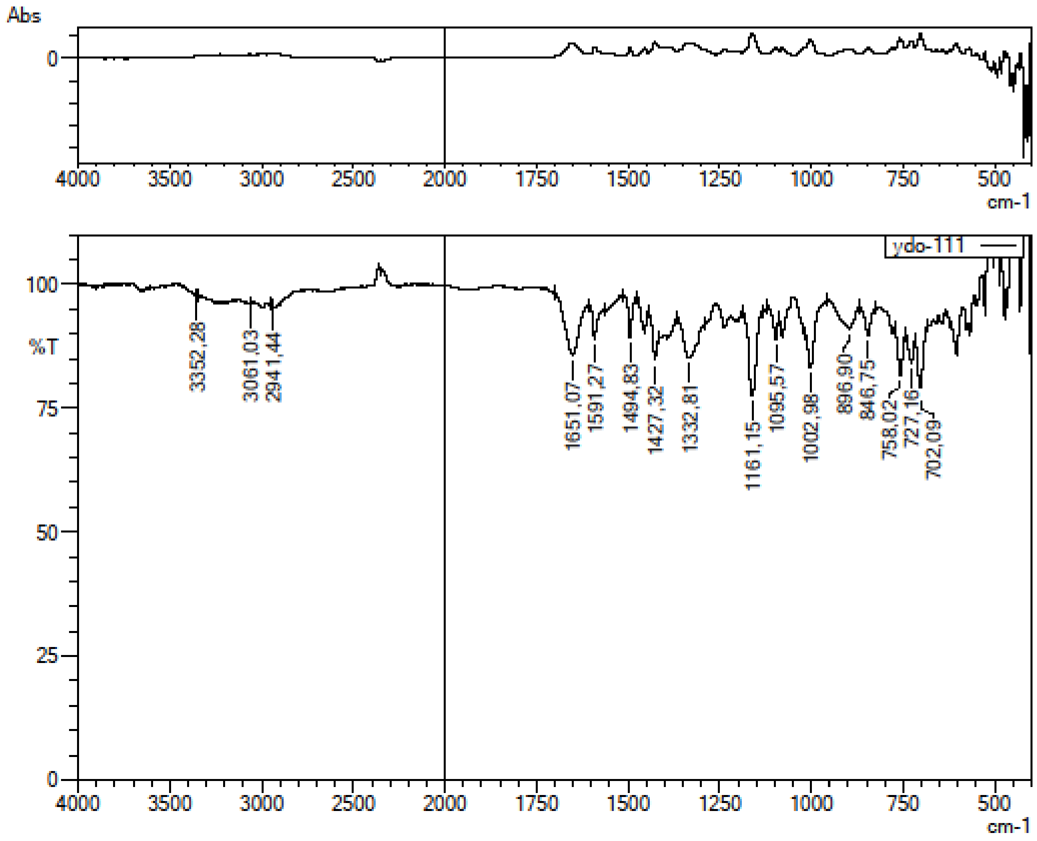

Spectra 29. IR spectra of compound **4h**

Formula Predictor Report - YD0-11_29.lcd Page 1 of 1

Data File: C:\LabSolutions\Data\Analiz\derya\YD0-11_29.lcd

Elmt	Val.	Min	Max	Elmt	Val.	Min	Max	Elmt	Val.	Min	Max	Elmt	Val.	Min	Max	Use Adduct
H	1	5	40	O	2	2	4	S	2	0	3	Ru	2	0	0	H
C	4	0	35	F	1	0	0	Cl	1	0	0	I	3	0	0	
N	3	3	6	P	3	0	0	Br	1	0	0					

Error Margin (ppm):	5	DBE Range:	6.0 - 20.0	Electron Ions:	both
HC Ratio:	unlimited	Apply N Rule:	yes	Use MSn Info:	yes
Max Isotopes:	3	Isotope RI (%):	1.00	Isotope Res:	9000
MSn Iso RI (%):	10.00	MSn Logic Mode:	AND	Max Results:	500

Event#: 1 MS(E+) Ret. Time : 6.467 -> 6.640 Scan# : 971 -> 997

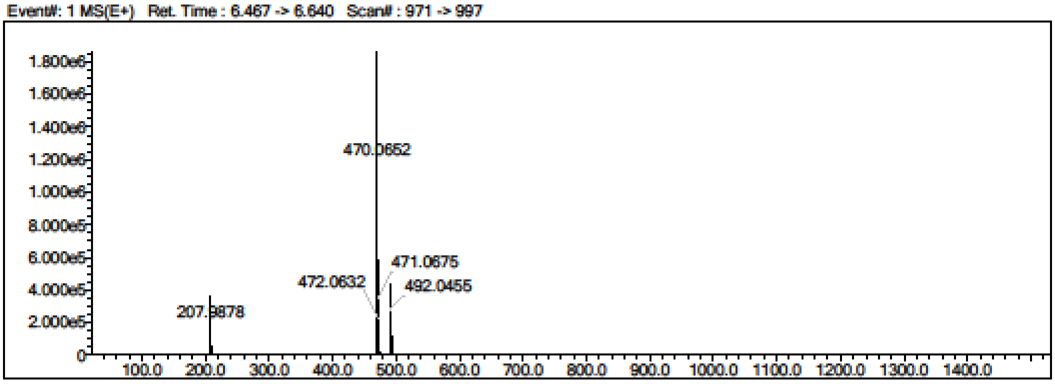

Measured region for 470.0652 m/z

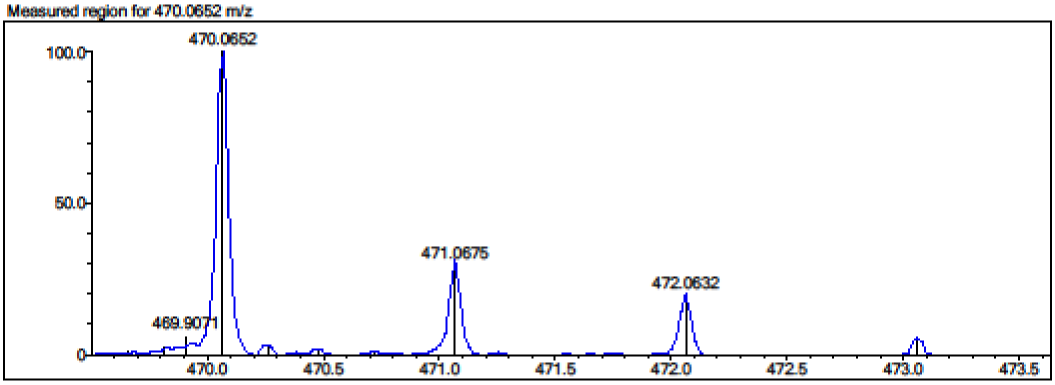

C22 H19 N3 O3 S3 [M+H]+ : Predicted region for 470.0661 m/z

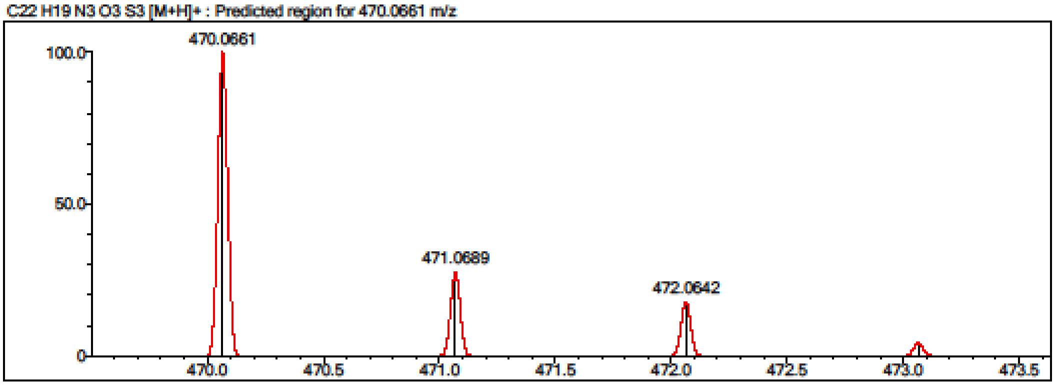

Rank	Score	Formula (M)	Ion	Meas. m/z	Pred. m/z	Df. (mDa)	Df. (ppm)	Iso	DBE
1	92.83	C22 H19 N3 O3 S3	[M+H]+	470.0652	470.0661	-0.9	-1.91	94.99	15.0

Spectra 30. HRMS spectra of compound **4h**

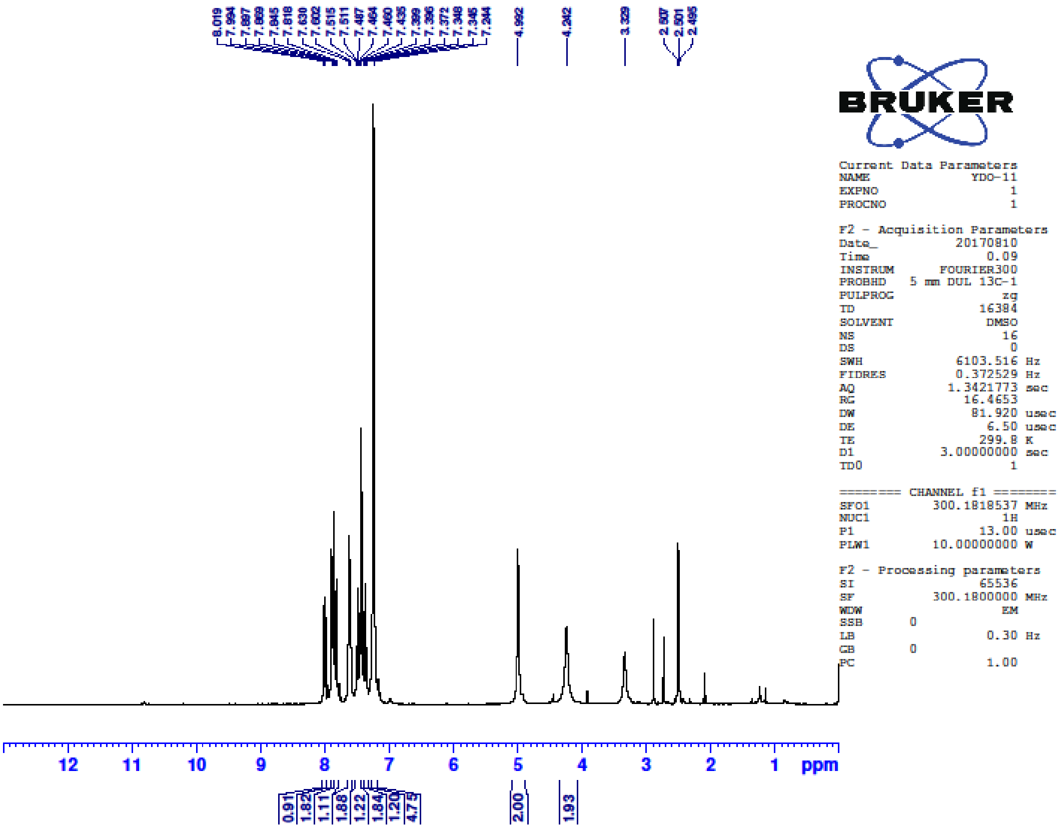

Spectra 31. ^{1}H-NMR spectra of compound **4h**

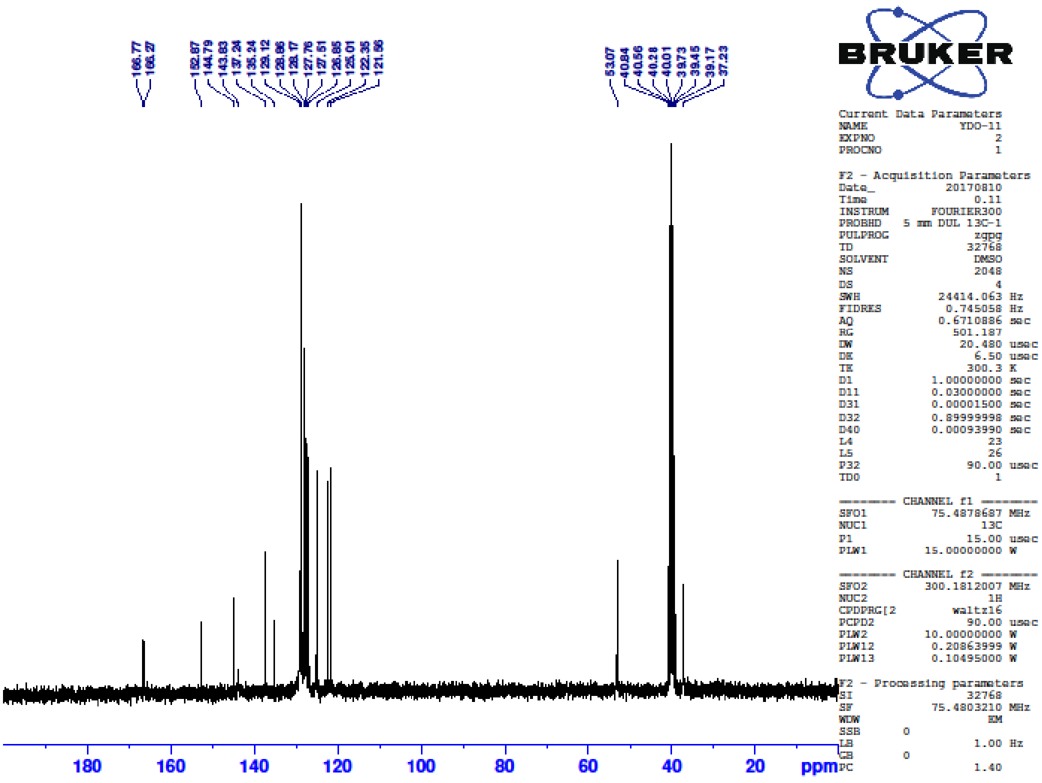

Spectra 32. ^{13}C-NMR spectra of compound **4h**

DOPNALAB

	Item	Value
	Acquired Date&Time	3.05.2018 13:32:48
	Acquired by	System Administrator
	Filename	C:\Users\dopnalab\Desktop\derya\ydo sensi\ydo-121.ispd
	Spectrum name	ydo-121
	Sample name	ydo-12
	Sample ID	
	Option	
	Comment	
	No. of Scans	10
	Resolution	4 [cm-1]
	Apodization	Happ-Genzel

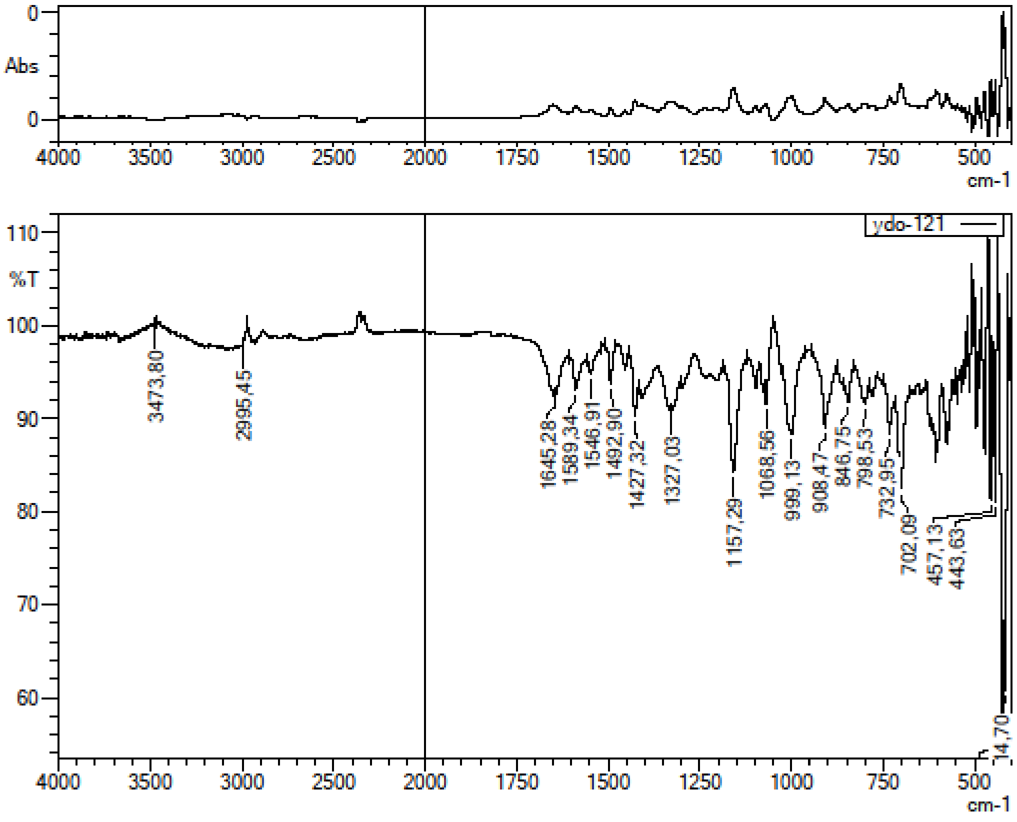

Spectra 33. IR spectra of compound **4i**

Formula Predictor Report - YD0-12_30.lcd Page 1 of 1

Data File: C:\LabSolutions\Data\Analiz\derya\YD0-12_30.lcd

Elmt	Val.	Min	Max	Elmt	Val.	Min	Max	Elmt	Val.	Min	Max	Elmt	Val.	Min	Max	Use Adduct
H	1	5	40	O	2	0	3	S	2	0	3	Ru	2	0	0	H
C	4	0	35	F	1	0	0	Cl	1	0	1	I	3	0	0	
N	3	3	7	P	3	0	0	Br	1	0	0					

Error Margin (ppm):	7	DBE Range:	6.0 - 19.0	Electron Ions:	both
HC Ratio:	unlimited	Apply N Rule:	yes	Use MSn Info:	yes
Max Isotopes:	3	Isotope RI (%):	1.00	Isotope Res:	9000
MSn Iso RI (%):	10.00	MSn Logic Mode:	AND	Max Results:	500

Event#: 1 MS(E+) Ret. Time : 7.027 Scan# : 1055

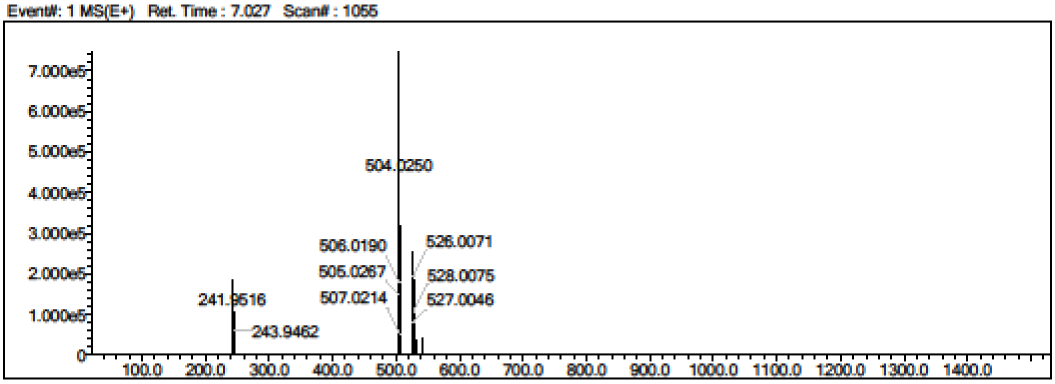

Measured region for 504.0250 m/z

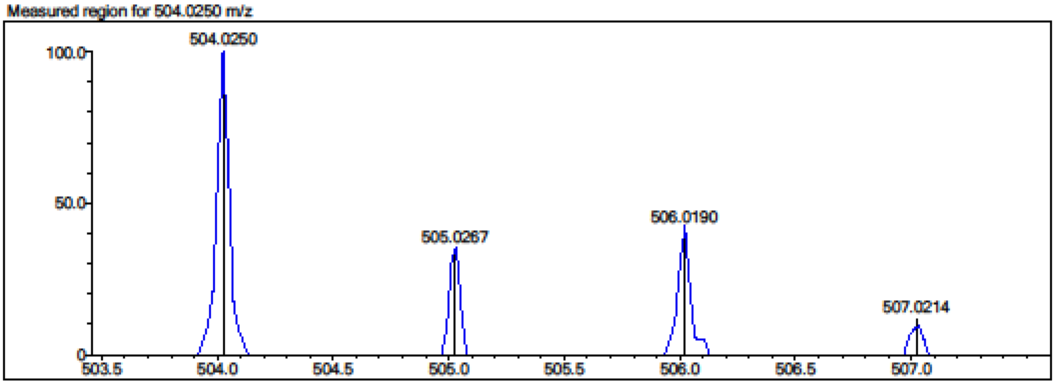

C22 H18 N3 O3 S3 Cl [M+H]+ : Predicted region for 504.0272 m/z

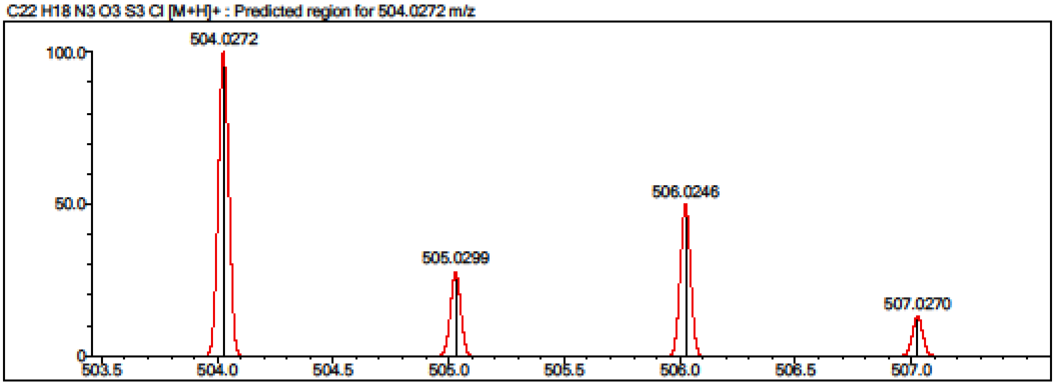

Rank	Score	Formula (M)	Ion	Meas. m/z	Pred. m/z	Df. (mDa)	Df. (ppm)	Iso	DBE
1	60.23	C22 H18 N3 O3 S3 Cl	[M+H]+	504.0250	504.0272	-2.2	-4.36	65.75	15.0

Spectra 34. HRMS spectra of compound **4i**

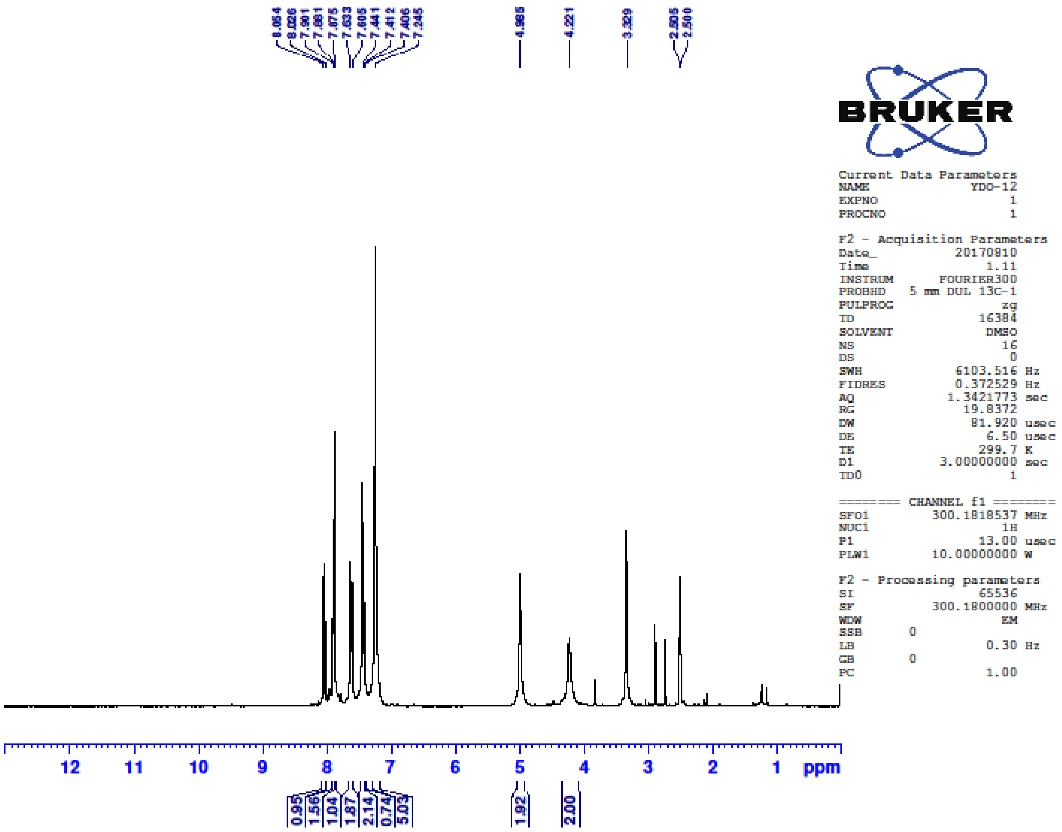

Spectra 35. ^{1}H-NMR spectra of compound **4i**

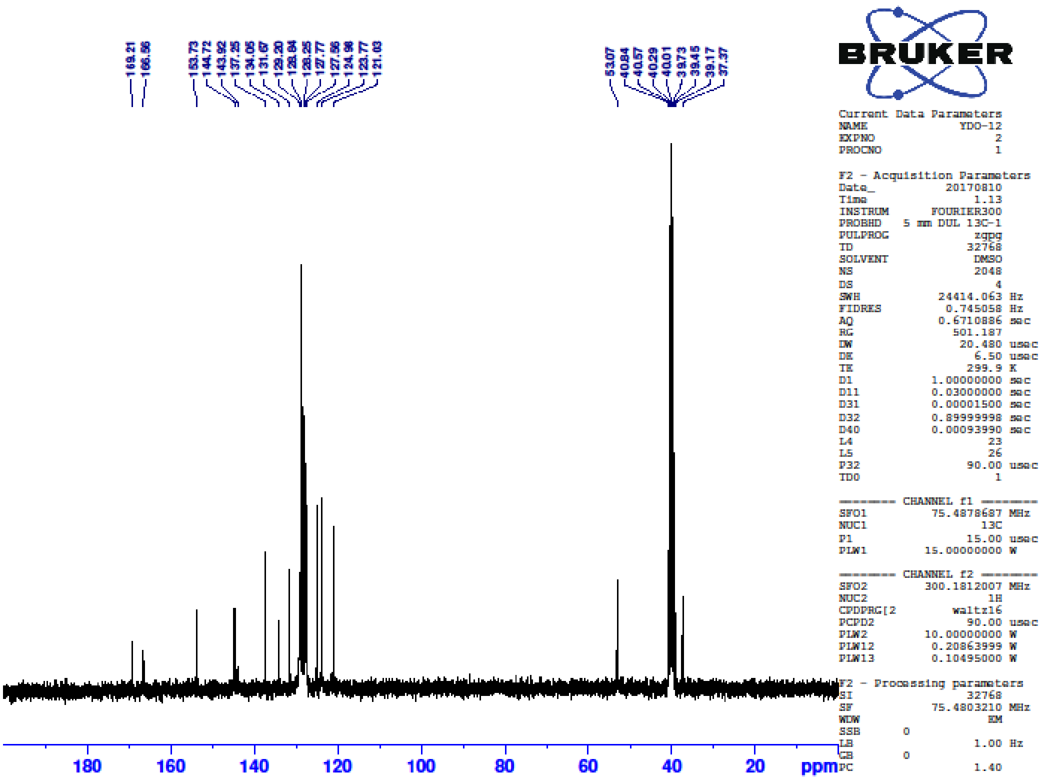

Spectra 36. ^{13}C-NMR spectra of compound **4i**

DOPNALAB

Item	Value
Acquired Date&Time	3.05.2018 13:35:40
Acquired by	System Administrator
Filename	C:\Users\dopnalab\Desktop\derya\ydo sersi\ydo-131.ispd
Spectrum name	ydo-131
Sample name	ydo-13
Sample ID	
Option	
Comment	
No. of Scans	10
Resolution	4 [cm-1]
Apodization	Happ-Genzel

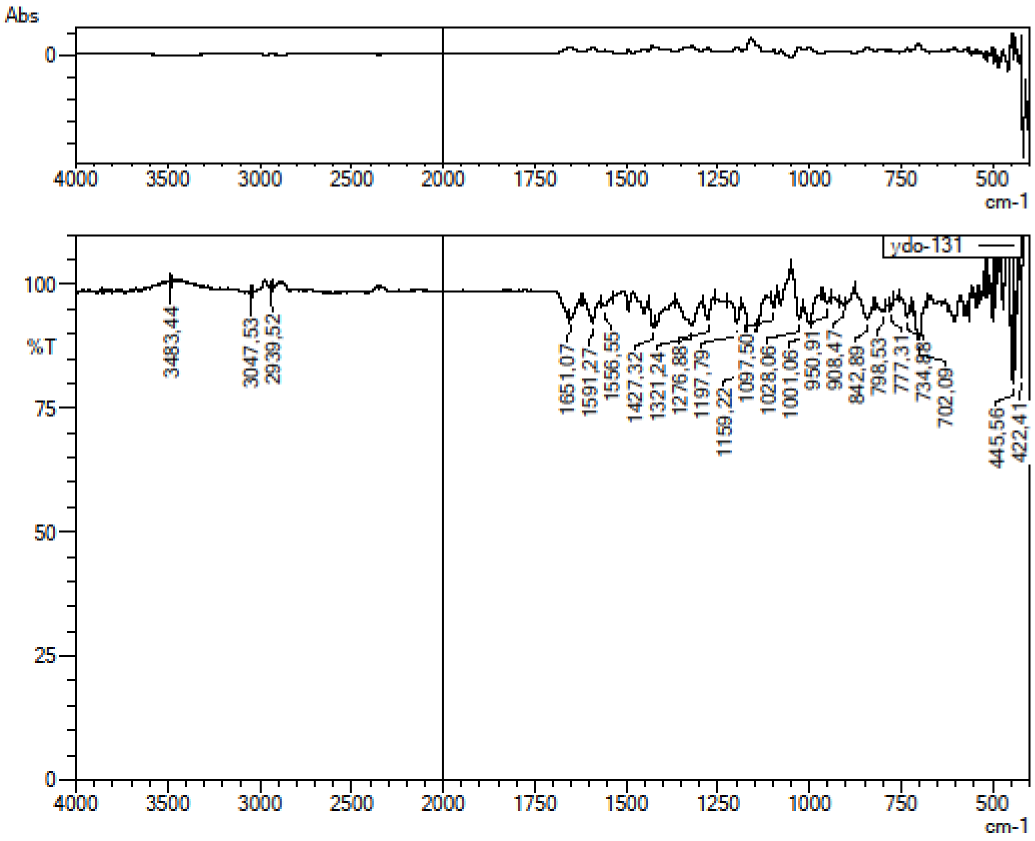

Spectra 37. IR spectra of compound **4j**

Formula Predictor Report - YD0-13_31.lcd

Data File: C:\LabSolutions\Data\Analiz\derya\YD0-13_31.lcd

Elmt	Val.	Min	Max	Elmt	Val.	Min	Max	Elmt	Val.	Min	Max	Elmt	Val.	Min	Max	Use Adduct
H	1	5	40	O	2	2	4	S	2	0	3	Ru	2	0	0	H
C	4	0	35	F	1	0	0	Cl	1	0	1	I	3	0	0	
N	3	3	4	P	3	0	0	Br	1	0	0					

Error Margin (ppm): 5
HC Ratio: unlimited
Max Isotopes: 3
MSn Iso RI (%): 10.00

DBE Range: 6.0 - 20.0
Apply N Rule: yes
Isotope RI (%): 1.00
MSn Logic Mode: AND

Electron Ions: both
Use MSn Info: yes
Isotope Res: 9000
Max Results: 500

Event#: 1 MS(E+) Ret. Time : 6.667 Scan# : 1001

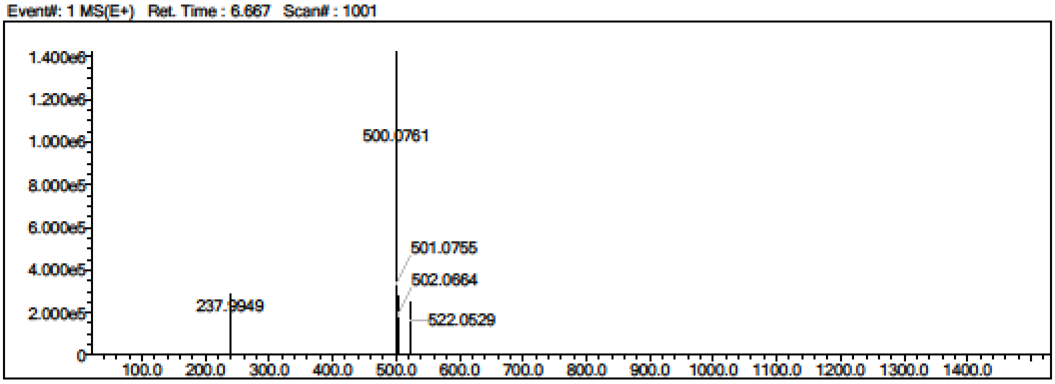

Measured region for 500.0761 m/z

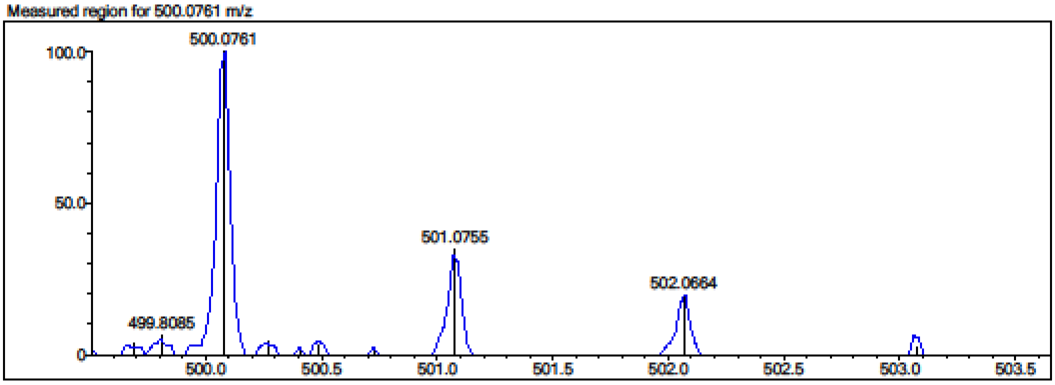

C23 H21 N3 O4 S3 [M+H]+ : Predicted region for 500.0767 m/z

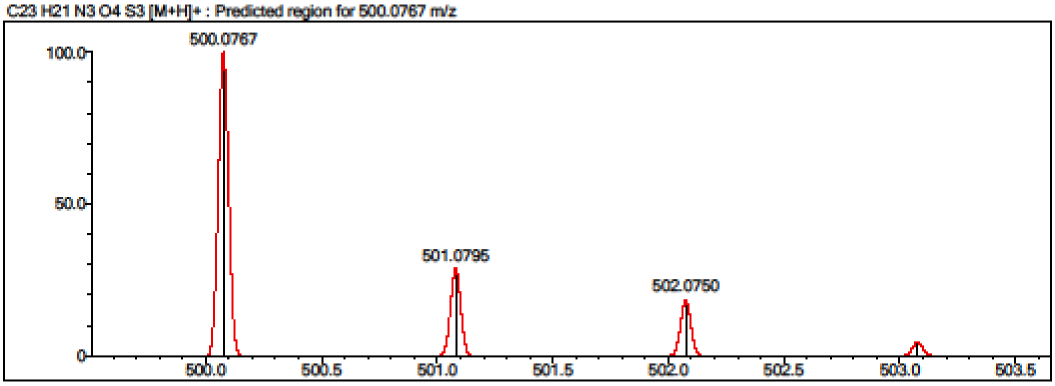

Rank	Score	Formula (M)	Ion	Meas. m/z	Pred. m/z	Df. (mDa)	Df. (ppm)	Iso	DBE
1	79.12	C23 H21 N3 O4 S3	[M+H]+	500.0761	500.0767	-0.6	-1.20	79.52	15.0

Spectra 38. HRMS spectra of compound **4j**

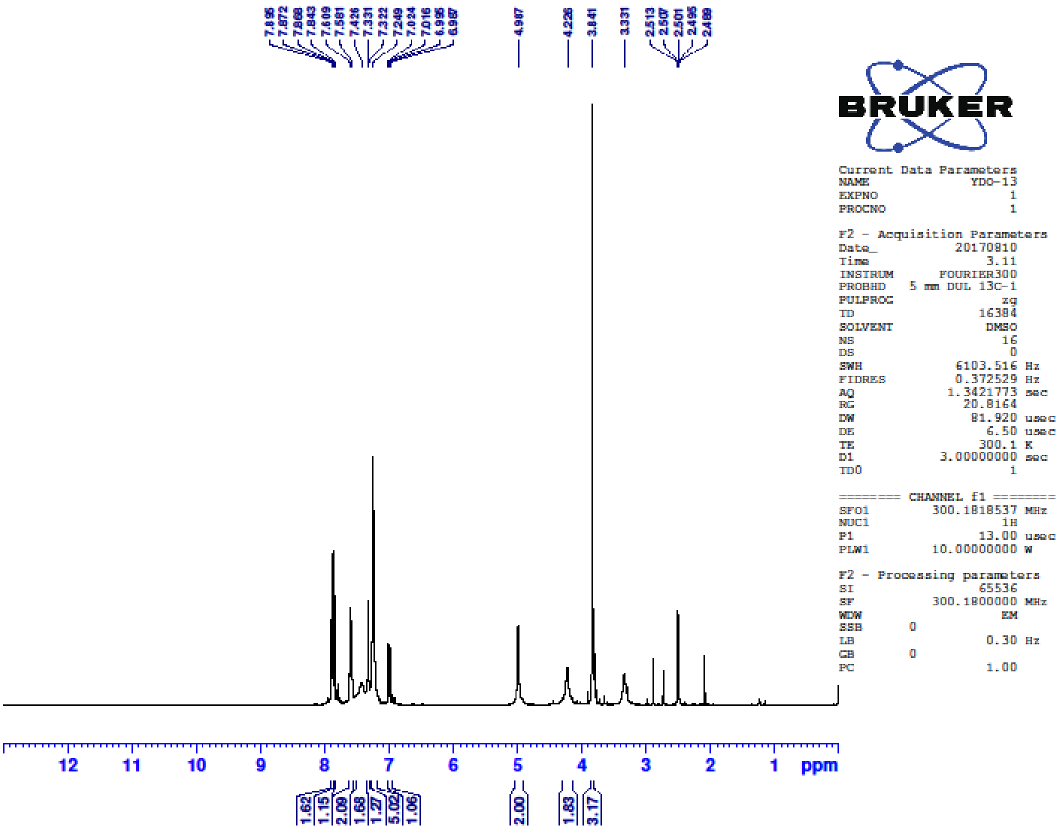

Spectra 39. ^{1}H-NMR spectra of compound **4j**

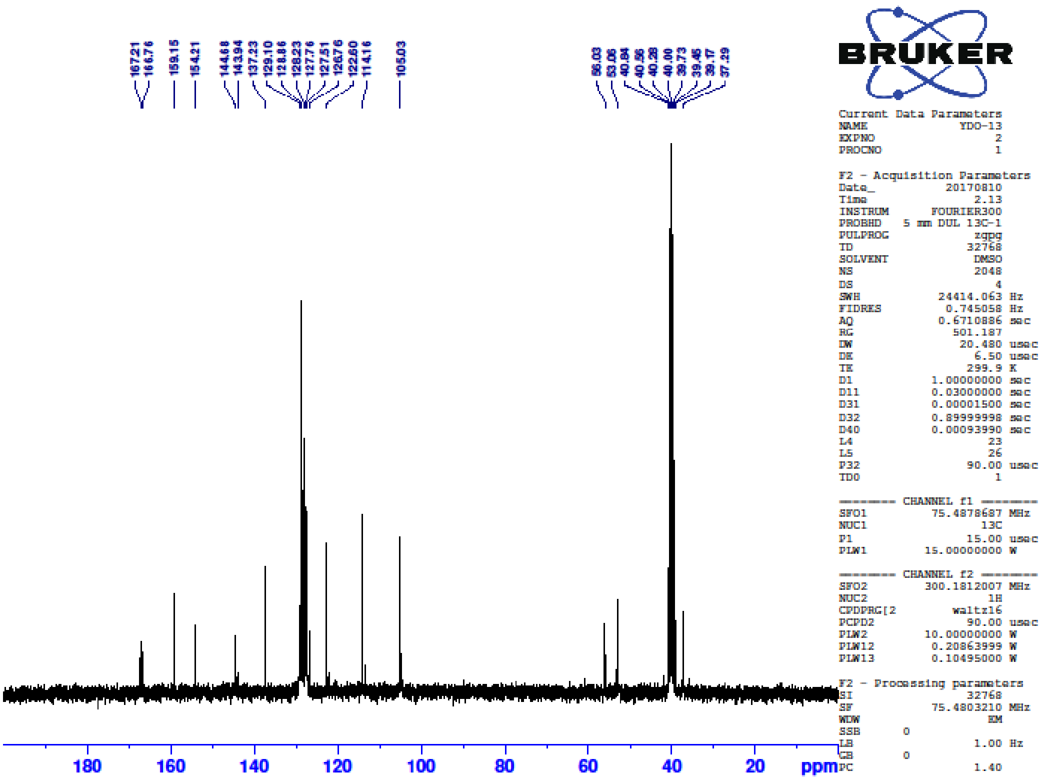

Spectra 40. ^{13}C-NMR spectra of compound **4j**

DOPNALAB

Item	Value
Acquired Date&Time	3.05.2018 13:40:24
Acquired by	System Administrator
Filename	C:\Users\dopnalab\Desktop\derya\ydo serisi\ydo-141.ispd
Spectrum name	ydo-141
Sample name	ydo-14
Sample ID	
Option	
Comment	
No. of Scans	10
Resolution	4 [cm-1]
Apodization	Happ-Genzel

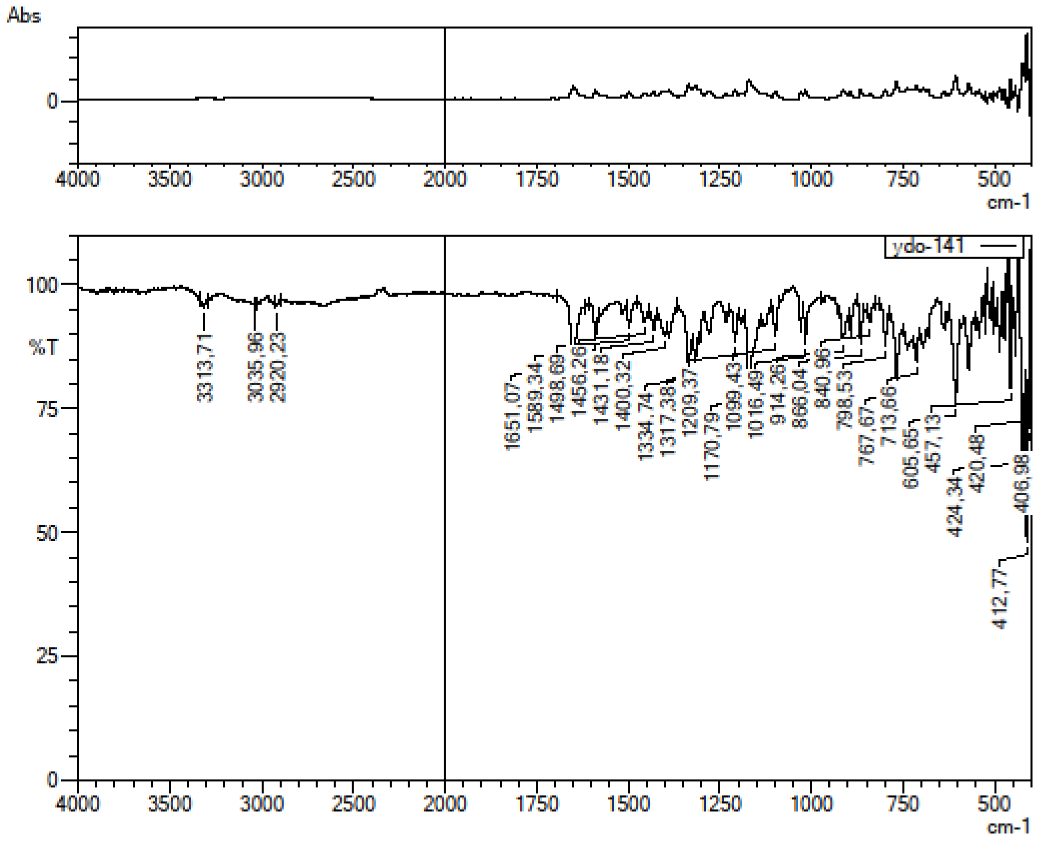

Spectra 41. IR spectra of compound **4k**

Data File: C:\LabSolutions\Data\Analiz\derya\YD0-14_32.lcd

Elmt	Val.	Min	Max	Elmt	Val.	Min	Max	Elmt	Val.	Min	Max	Elmt	Val.	Min	Max	Use Adduct
H	1	5	40	O	2	2	4	S	2	0	3	Ru	2	0	0	H
C	4	0	35	F	1	0	0	Cl	1	0	1	I	3	0	0	
N	3	3	4	P	3	0	0	Br	1	0	0					

Error Margin (ppm): 5
HC Ratio: unlimited
Max Isotopes: 3
MSn Iso RI (%): 10.00

DBE Range: 6.0 - 20.0
Apply N Rule: yes
Isotope RI (%): 1.00
MSn Logic Mode: AND

Electron Ions: both
Use MSn Info: yes
Isotope Res: 9000
Max Results: 500

Event#: 1 MS(E+) Ret. Time : 3.520 -> 3.707 Scan# : 529 -> 557

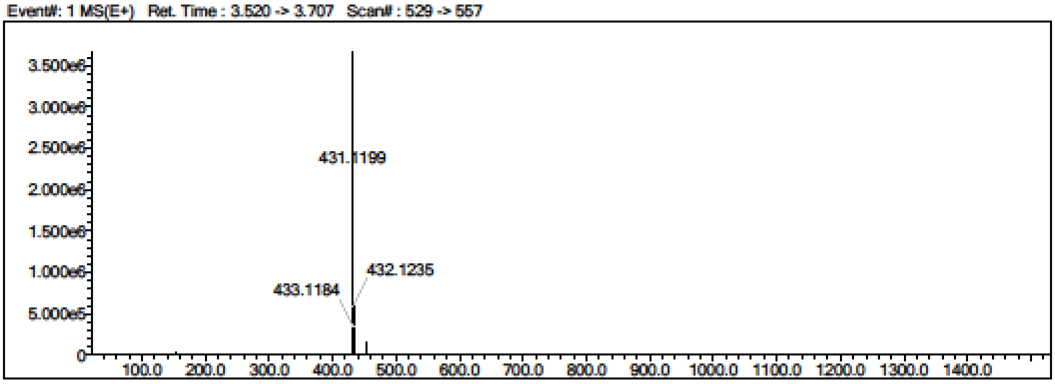

Measured region for 431.1199 m/z

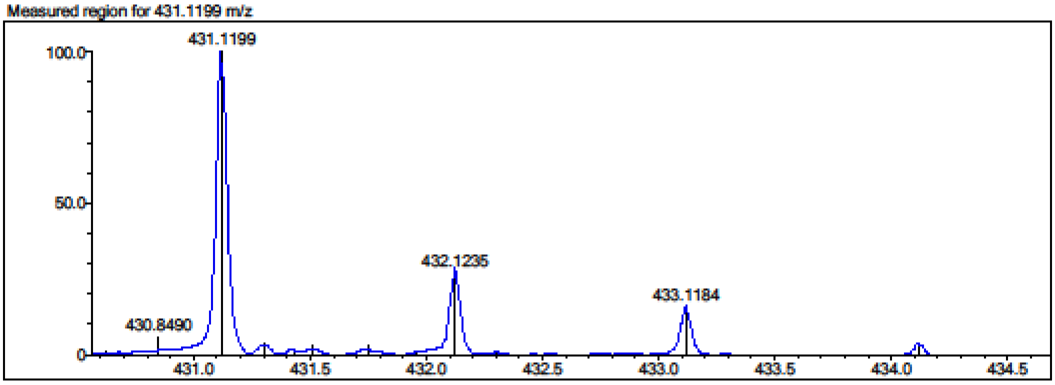

C20 H22 N4 O3 S2 [M+H]+ : Predicted region for 431.1206 m/z

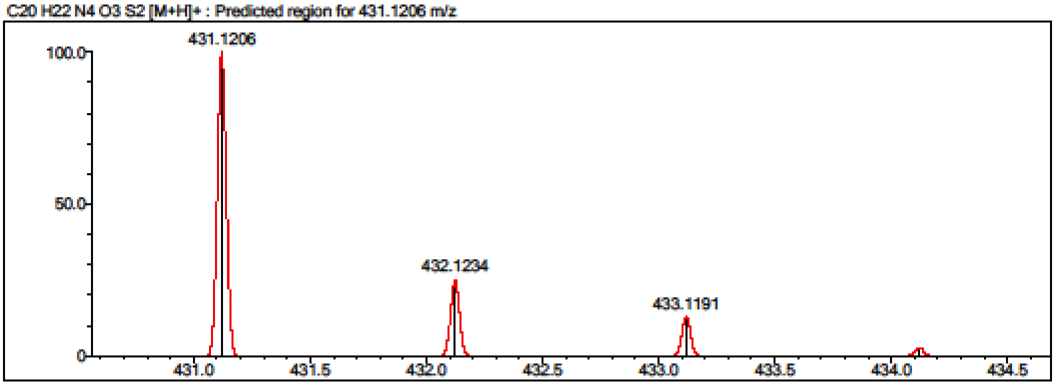

Rank	Score	Formula (M)	Ion	Meas. m/z	Pred. m/z	Df. (mDa)	Df. (ppm)	Iso	DBE
1	88.15	C20 H22 N4 O3 S2	[M+H]+	431.1199	431.1206	-0.7	-1.62	89.53	12.0

Spectra 42. HRMS spectra of compound **4k**

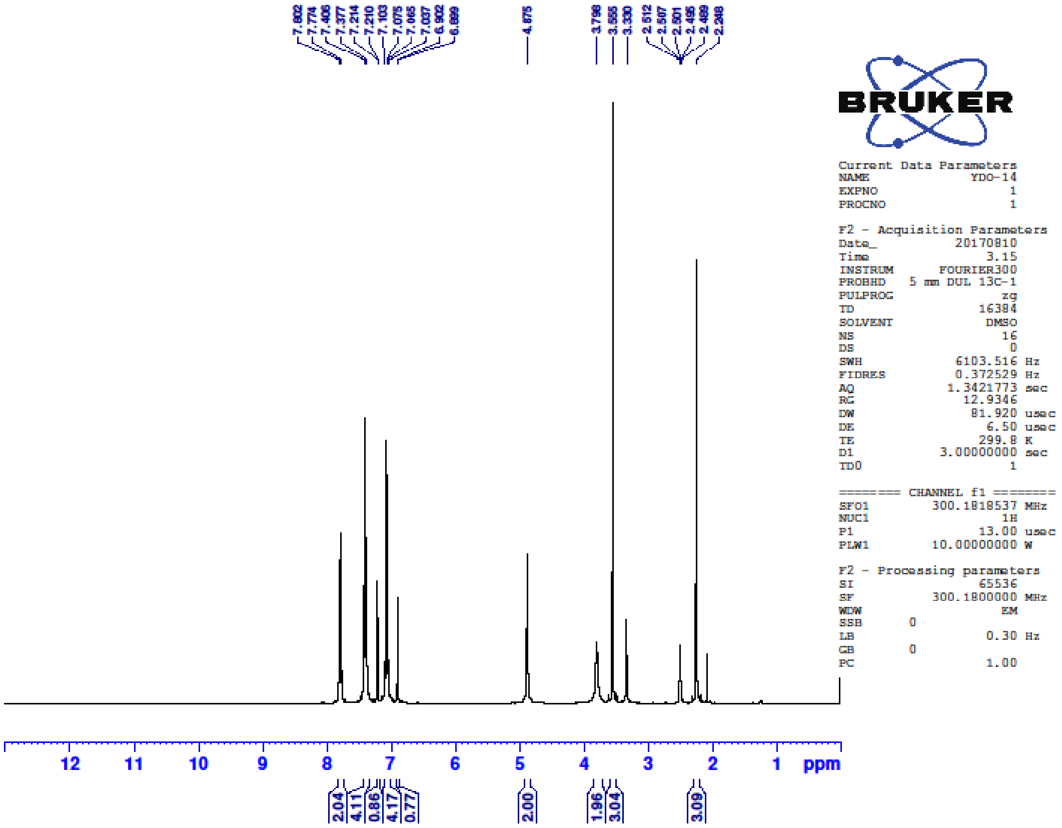

Spectra 43. ^{1}H-NMR spectra of compound **4k**

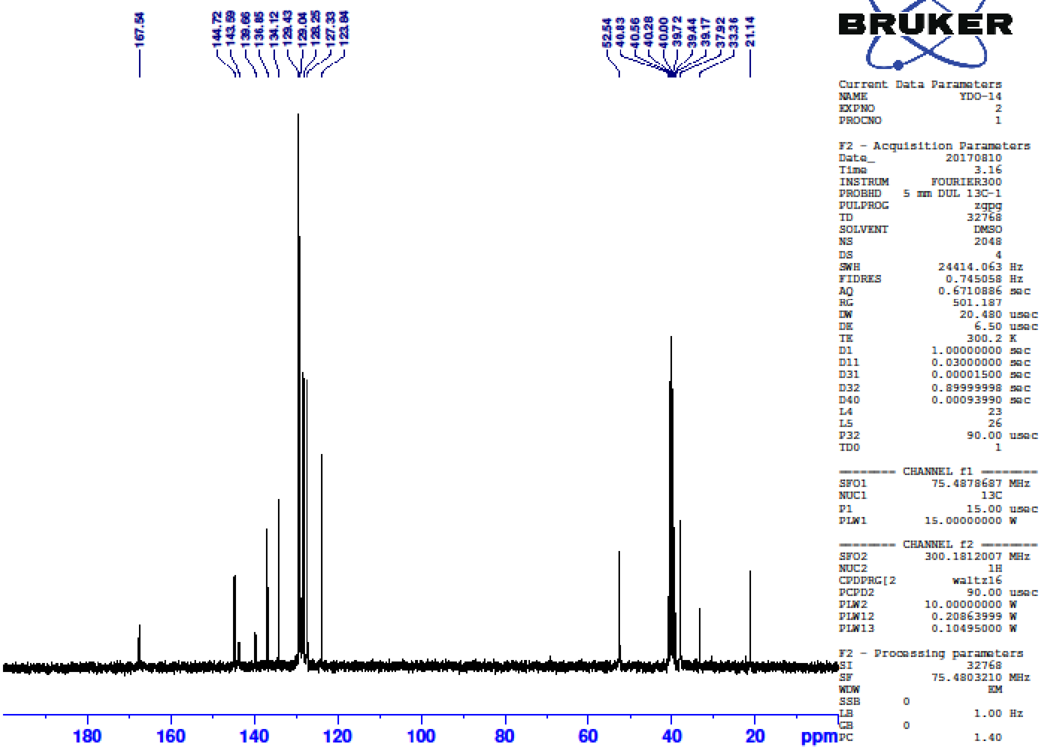

Spectra 44. ^{13}C-NMR spectra of compound **4k**

DOPNALAB

Item	Value
Acquired Date&Time	3.05.2018 13:43:27
Acquired by	System Administrator
Filename	C:\Users\dopnalab\Desktop\derya\ydo serisi\ydo-161.ispd
Spectrum name	ydo-161
Sample name	ydo-16
Sample ID	
Option	
Comment	
No. of Scans	10
Resolution	4 [cm-1]
Apodization	Happ-Genzel

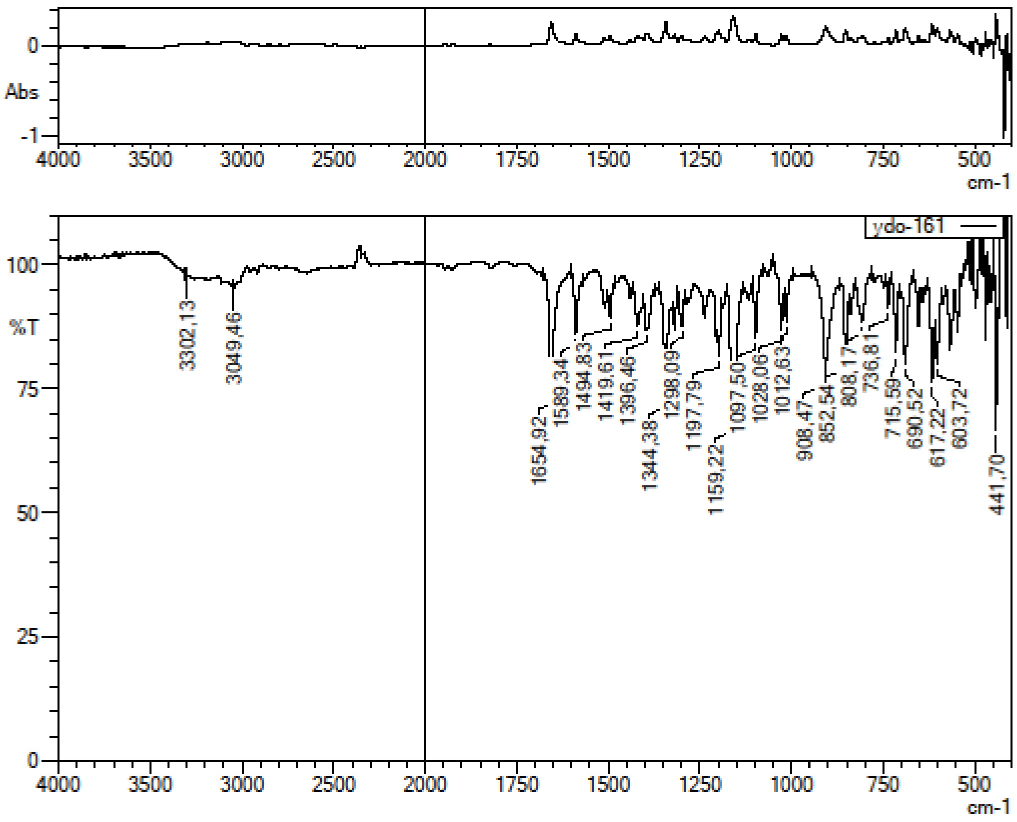

Spectra 45. IR spectra of compound **4l**

Formula Predictor Report - YDO-16_33.lcd

Data File: C:\LabSolutions\Data\Analiz\derya\YDO-16_33.lcd

Elmt	Val.	Min	Max	Elmt	Val.	Min	Max	Elmt	Val.	Min	Max	Elmt	Val.	Min	Max	Use Adduct
H	1	5	40	O	2	2	4	S	2	0	3	Ru	2	0	0	H
C	4	0	35	F	1	0	0	Cl	1	0	0	I	3	0	0	
N	3	3	5	P	3	0	0	Br	1	0	0					

Error Margin (ppm):	5	DBE Range:	6.0 - 20.0	Electron Ions:	both
HC Ratio:	unlimited	Apply N Rule:	yes	Use MSn Info:	yes
Max Isotopes:	3	Isotope RI (%):	1.00	Isotope Res:	9000
MSn Iso RI (%):	10.00	MSn Logic Mode:	AND	Max Results:	500

Event#: 1 MS(E+) Ret. Time : 4.853 -> 5.027 Scan# : 729 -> 755

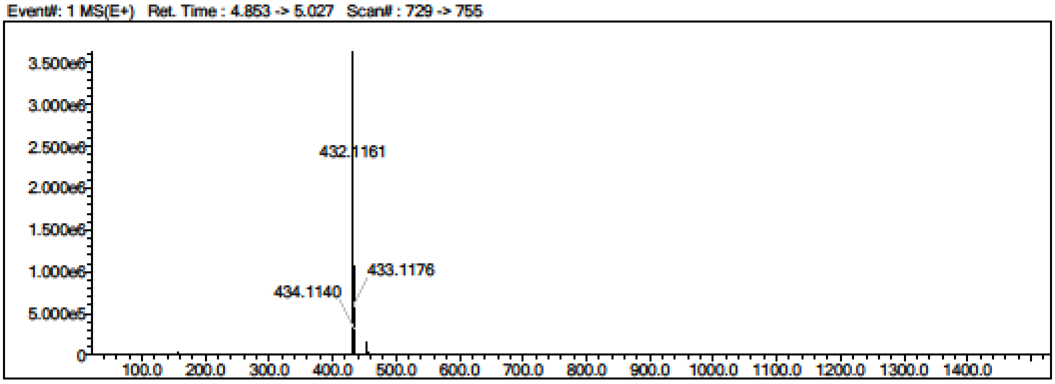

Measured region for 432.1161 m/z

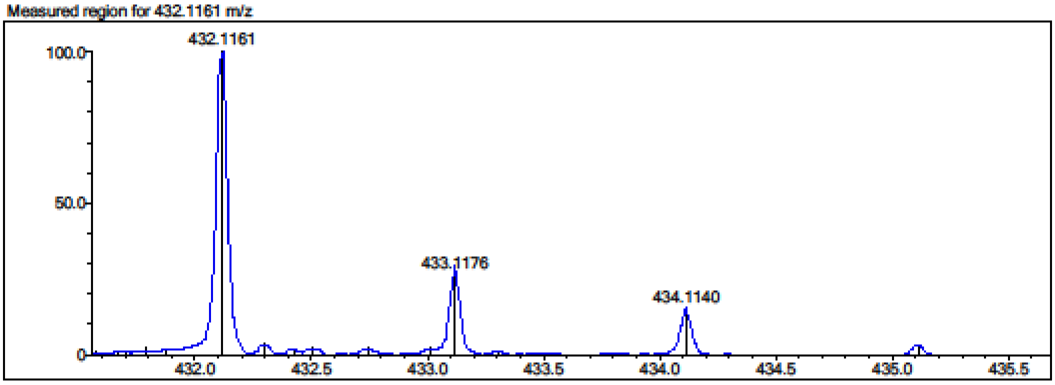

C19 H21 N5 O3 S2 [M+H]+ : Predicted region for 432.1159 m/z

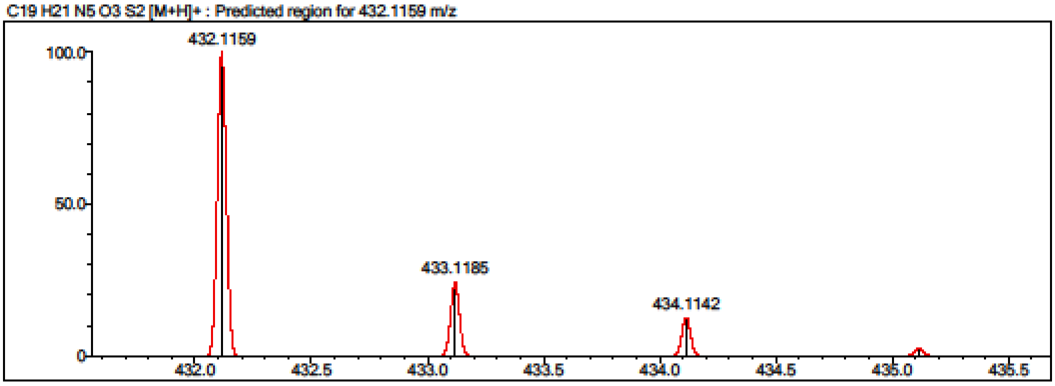

Rank	Score	Formula (M)	Ion	Meas. m/z	Pred. m/z	Df. (mDa)	Df. (ppm)	Iso	DBE
1	93.17	C19 H21 N5 O3 S2	[M+H]+	432.1161	432.1159	0.2	0.46	93.17	12.0

Spectra 46. HRMS spectra of compound **4l**

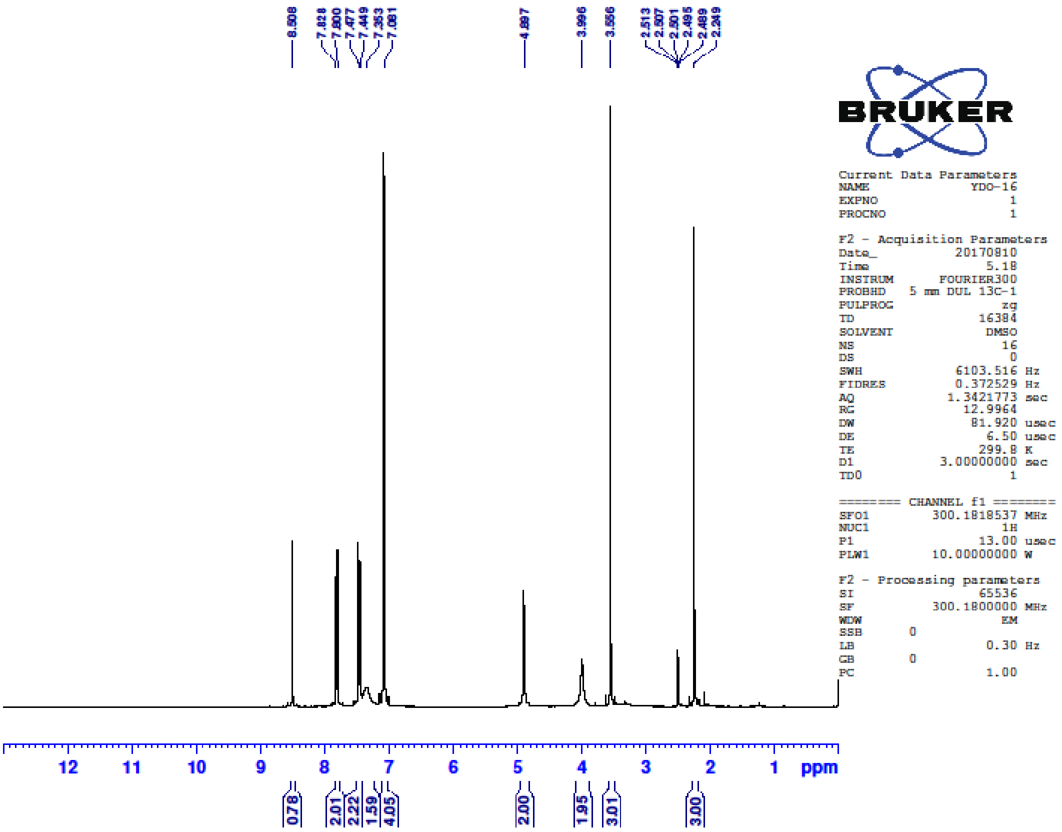

Spectra 47. ^{1}H-NMR spectra of compound **4l**

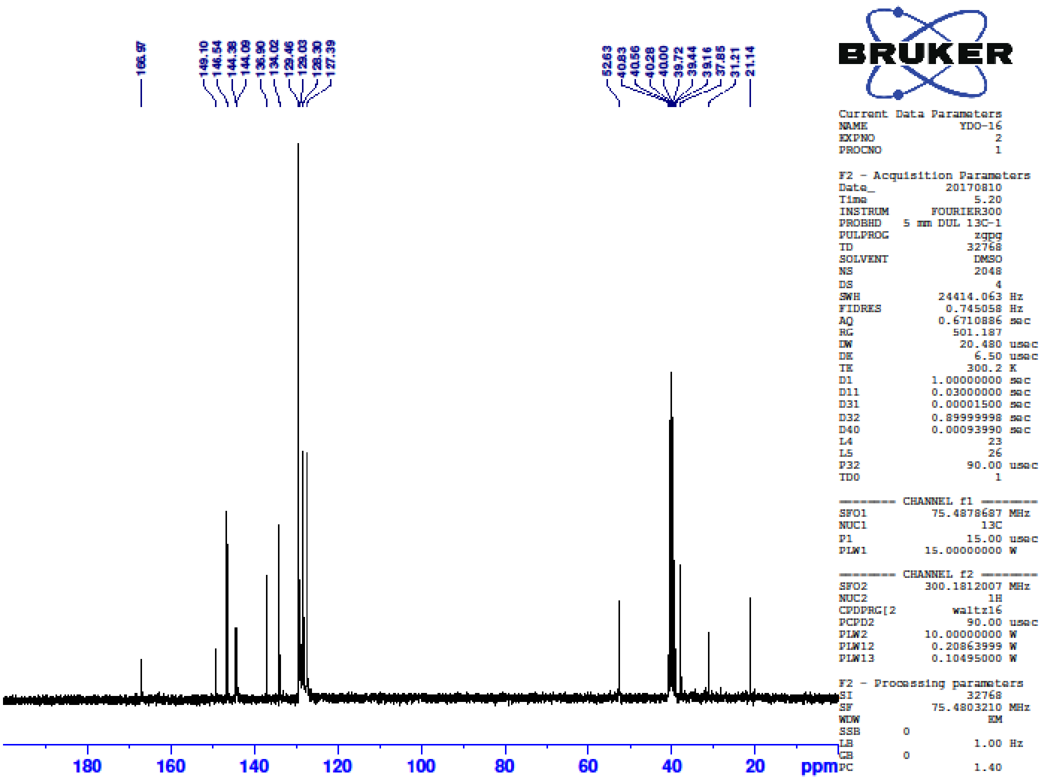

Spectra 48. ^{13}C-NMR spectra of compound **4l**

DOPNALAB

Item	Value
Acquired Date&Time	3.05.2018 13:47:06
Acquired by	System Administrator
Filename	C:\Users\dopnalab\Desktop\derya\ydo serisi\ydo-171.ispd
Spectrum name	ydo-171
Sample name	ydo-17
Sample ID	
Option	
Comment	
No. of Scans	10
Resolution	4 [cm-1]
Apodization	Happ-Genzel

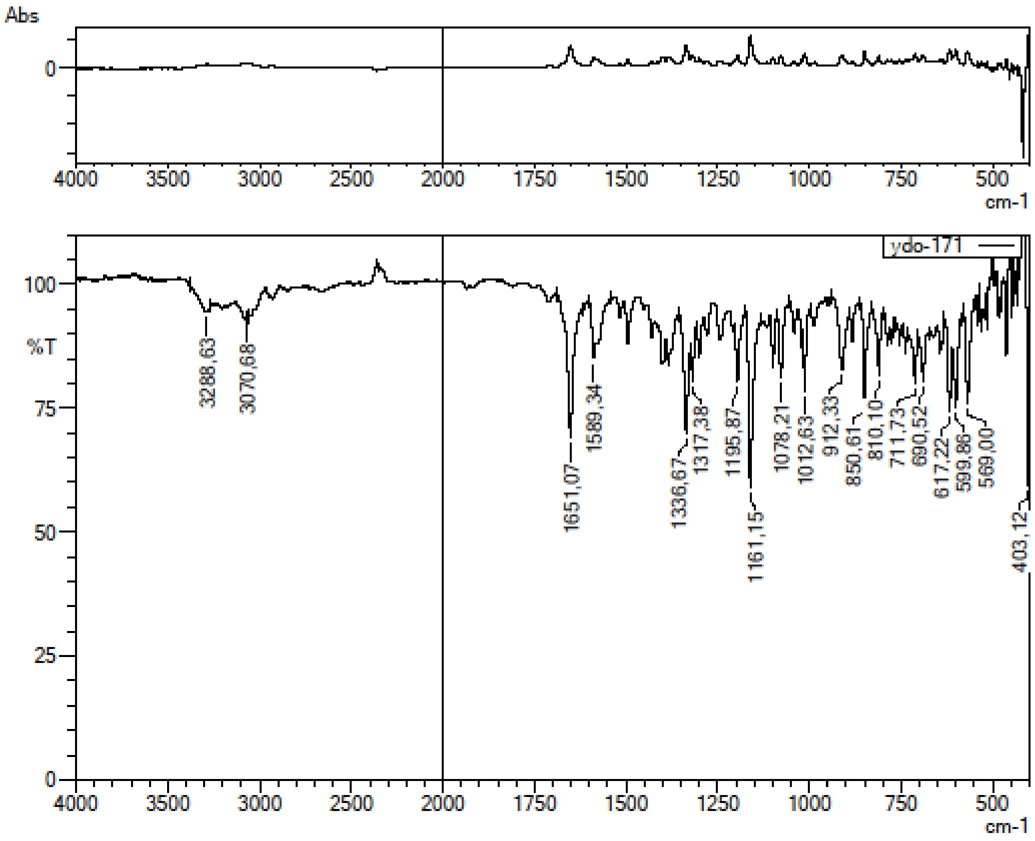

Spectra 49. IR spectra of compound **4m**

Formula Predictor Report - YDO-17_34.lcd

Data File: C:\LabSolutions\Data\Analiz\derya\YDO-17_34.lcd

Elmt	Val.	Min	Max	Elmt	Val.	Min	Max	Elmt	Val.	Min	Max	Elmt	Val.	Min	Max	Use Adduct
H	1	5	40	O	2	2	4	S	2	0	3	Ru	2	0	0	H
C	4	0	35	F	1	0	0	Cl	1	0	0	I	3	0	0	
N	3	3	5	P	3	0	0	Br	1	0	0					

Error Margin (ppm): 5
HC Ratio: unlimited
Max Isotopes: 3
MSn Iso RI (%): 10.00

DBE Range: 6.0 - 16.0
Apply N Rule: yes
Isotope RI (%): 1.00
MSn Logic Mode: AND

Electron Ions: both
Use MSn Info: yes
Isotope Res: 9000
Max Results: 500

Event#: 1 MS(E+) Ret. Time : 5.693 -> 5.773 Scan# : 855 -> 867

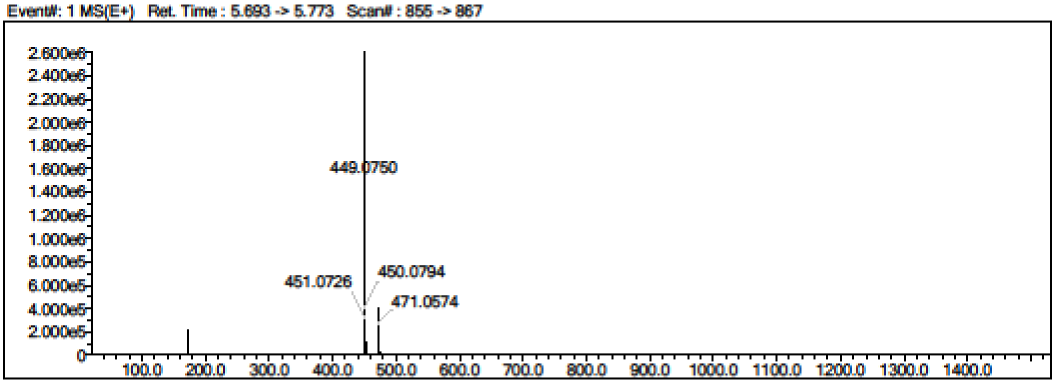

Measured region for 449.0750 m/z

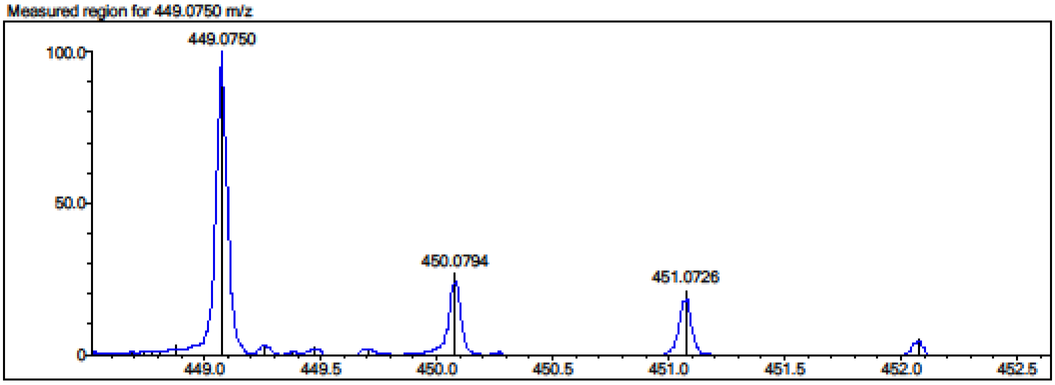

C19 H20 N4 O3 S3 [M+H]+ : Predicted region for 449.0770 m/z

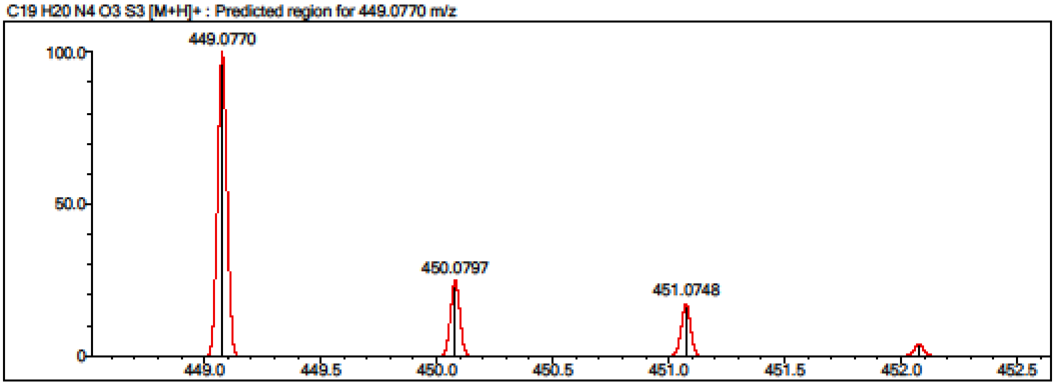

Rank	Score	Formula (M)	Ion	Meas. m/z	Pred. m/z	Df. (mDa)	Df. (ppm)	Iso	DBE
1	81.85	C19 H20 N4 O3 S3	[M+H]+	449.0750	449.0770	-2.0	-4.45	89.58	12.0

Spectra 50. HRMS spectra of compound **4m**

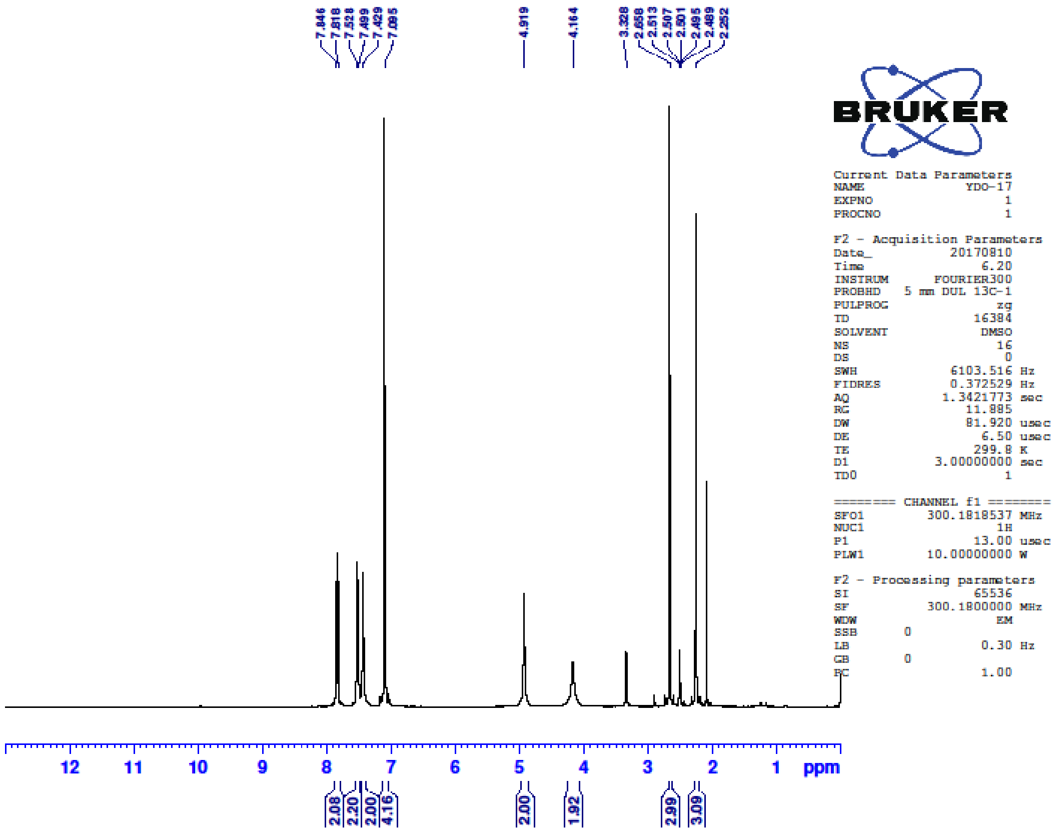

Spectra 51. ^{1}H-NMR spectra of compound **4m**

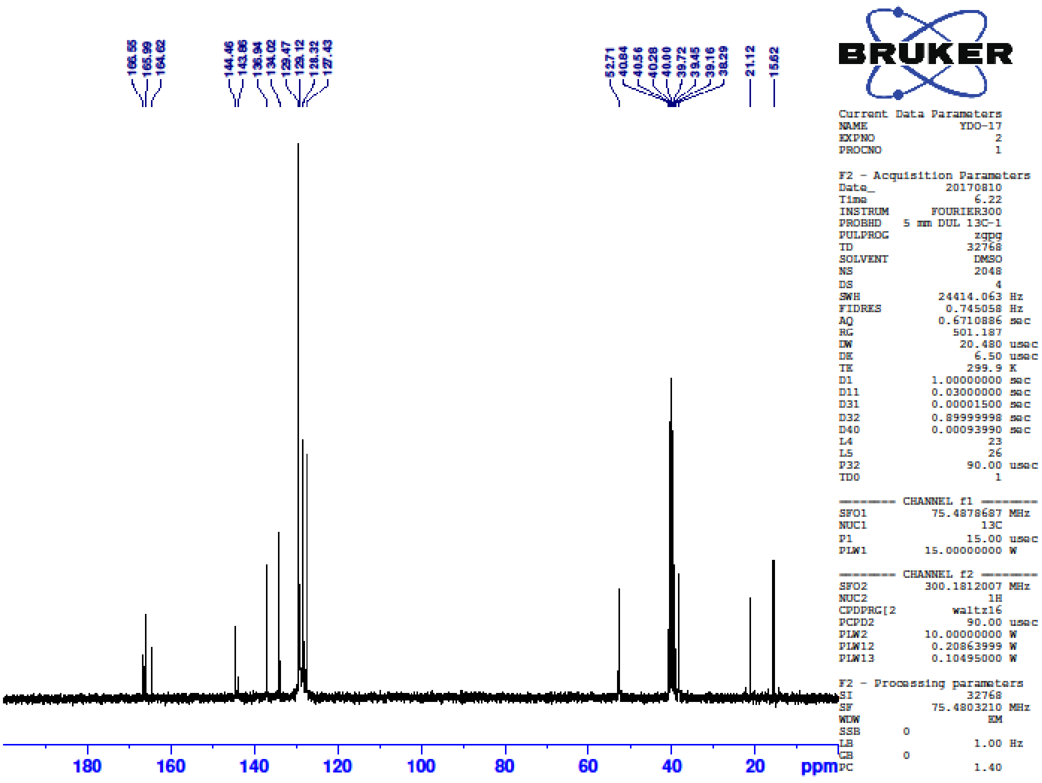

Spectra 52. ^{13}C-NMR spectra of compound **4m**

DOPNALAB

	Item	Value
	Acquired Date&Time	3.05.2018 13:49:35
	Acquired by	System Administrator
	Filename	C:\Users\dopnalab\Desktop\derya\ydo sensi\ydo-181.ispd
	Spectrum name	ydo-181
	Sample name	ydo-18
	Sample ID	
	Option	
	Comment	
	No. of Scans	10
	Resolution	4 [cm-1]
	Apodization	Happ-Genzel

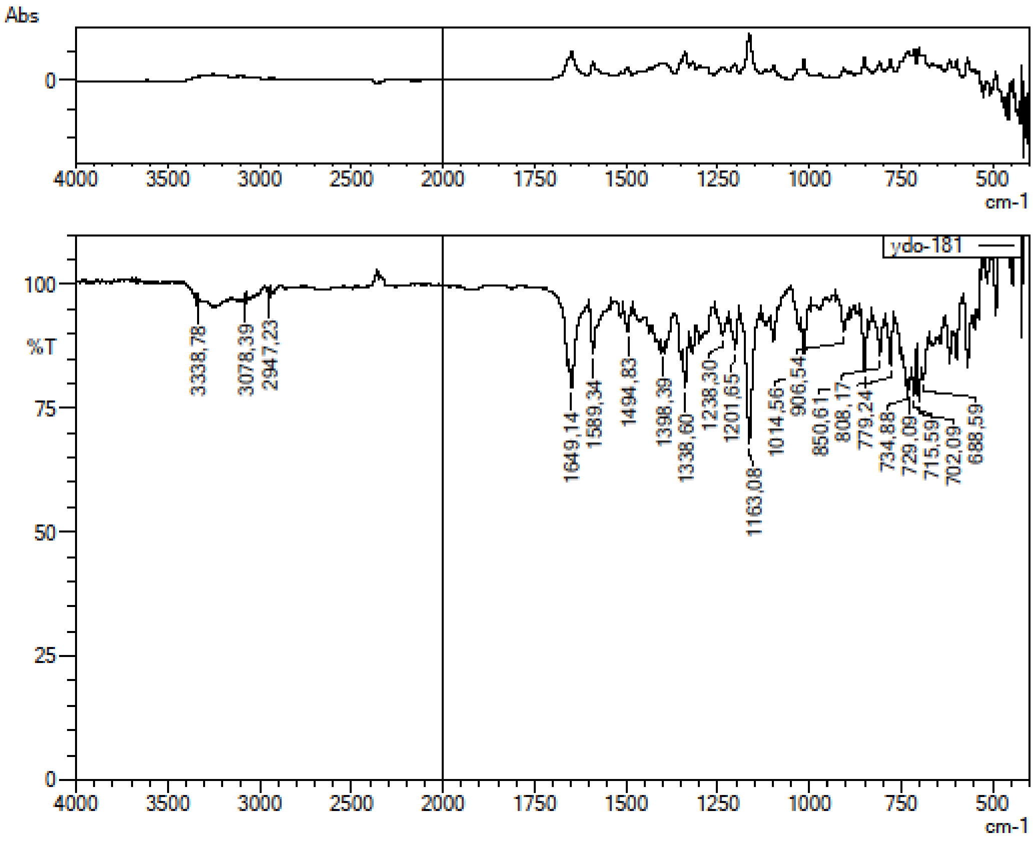

Spectra 53. IR spectra of compound **4n**

Data File: C:\LabSolutions\Data\Analiz\derya\YDO-18_35.lcd

Elmt	Val.	Min	Max	Elmt	Val.	Min	Max	Elmt	Val.	Min	Max	Elmt	Val.	Min	Max	Use Adduct
H	1	5	40	O	2	2	4	S	2	0	3	Ru	2	0	0	H
C	4	0	35	F	1	0	0	Cl	1	0	0	I	3	0	0	
N	3	3	6	P	3	0	0	Br	1	0	0					

Error Margin (ppm):	5	DBE Range:	6.0 - 16.0	Electron Ions:	both
HC Ratio:	unlimited	Apply N Rule:	yes	Use MSn Info:	yes
Max Isotopes:	3	Isotope RI (%):	1.00	Isotope Res:	9000
MSn Iso RI (%):	10.00	MSn Logic Mode:	AND	Max Results:	500

Event#: 1 MS(E+) Ret. Time : 5.600 -> 5.693 Scan# : 841 -> 855

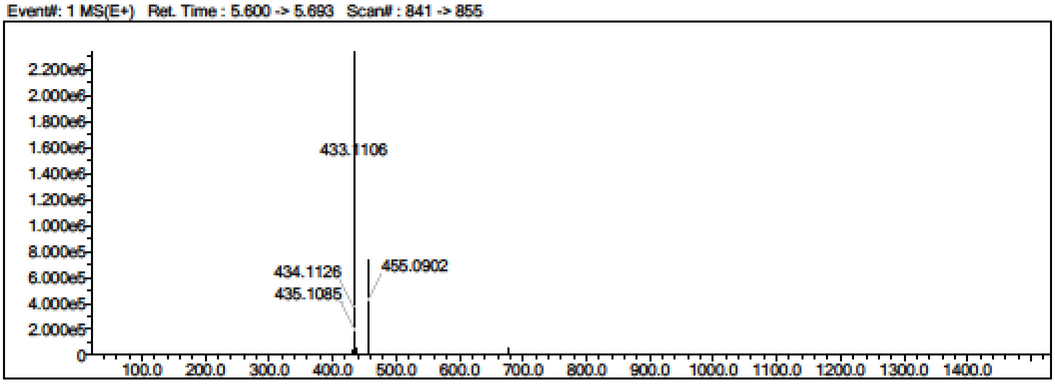

Measured region for 433.1106 m/z

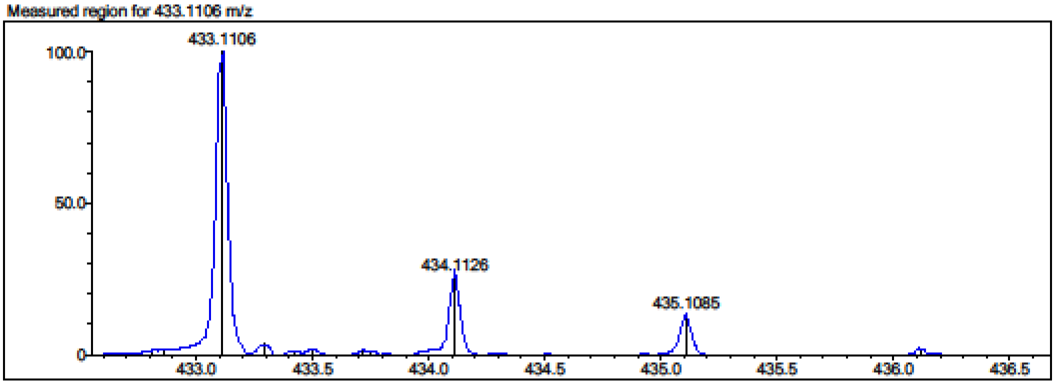

C18 H20 N6 O3 S2 [M+H]+ : Predicted region for 433.1111 m/z

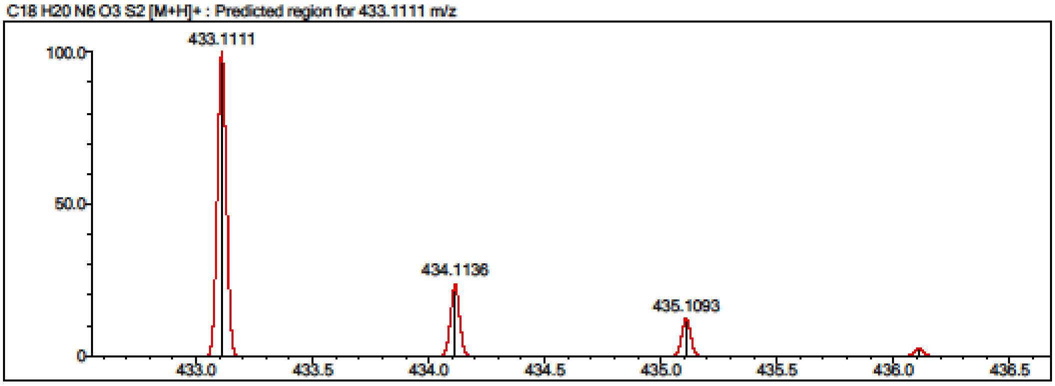

Rank	Score	Formula (M)	Ion	Meas. m/z	Pred. m/z	Df. (mDa)	Df. (ppm)	Iso	DBE
1	94.35	C18 H20 N6 O3 S2	[M+H]+	433.1106	433.1111	-0.5	-1.15	94.71	12.0

Spectra 54. HRMS spectra of compound **4n**

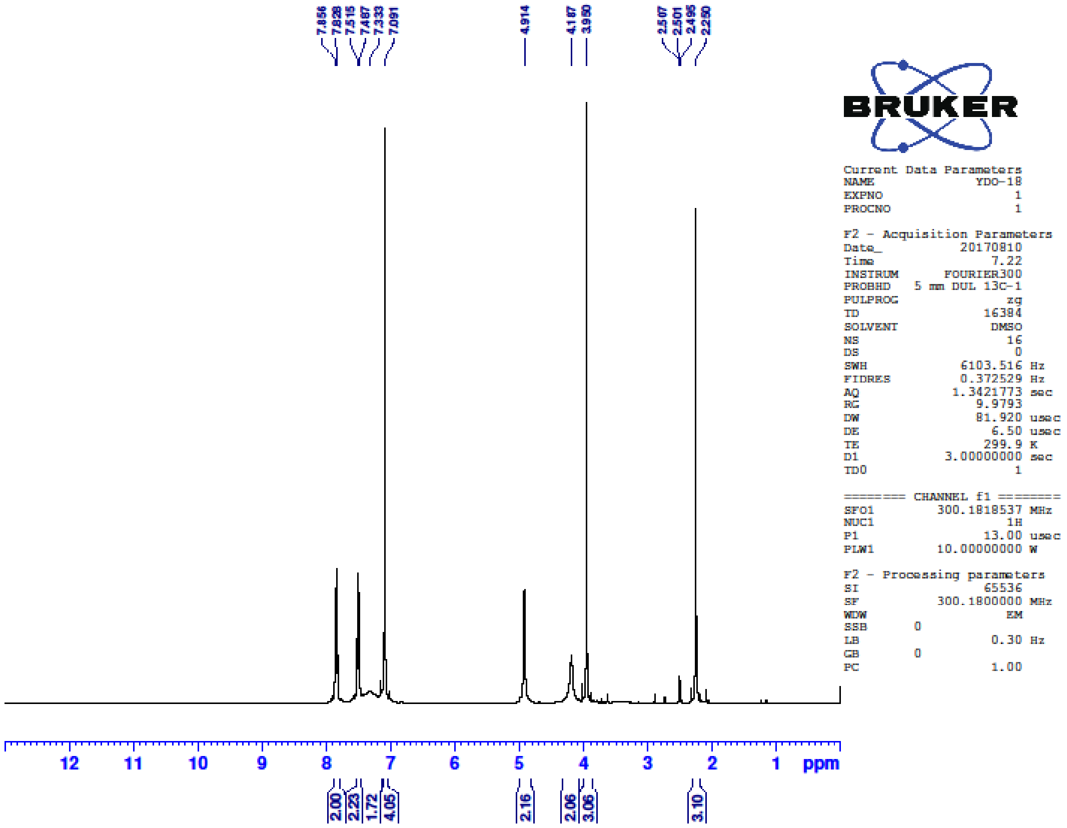

Spectra 55. ¹H-NMR spectra of compound **4n**

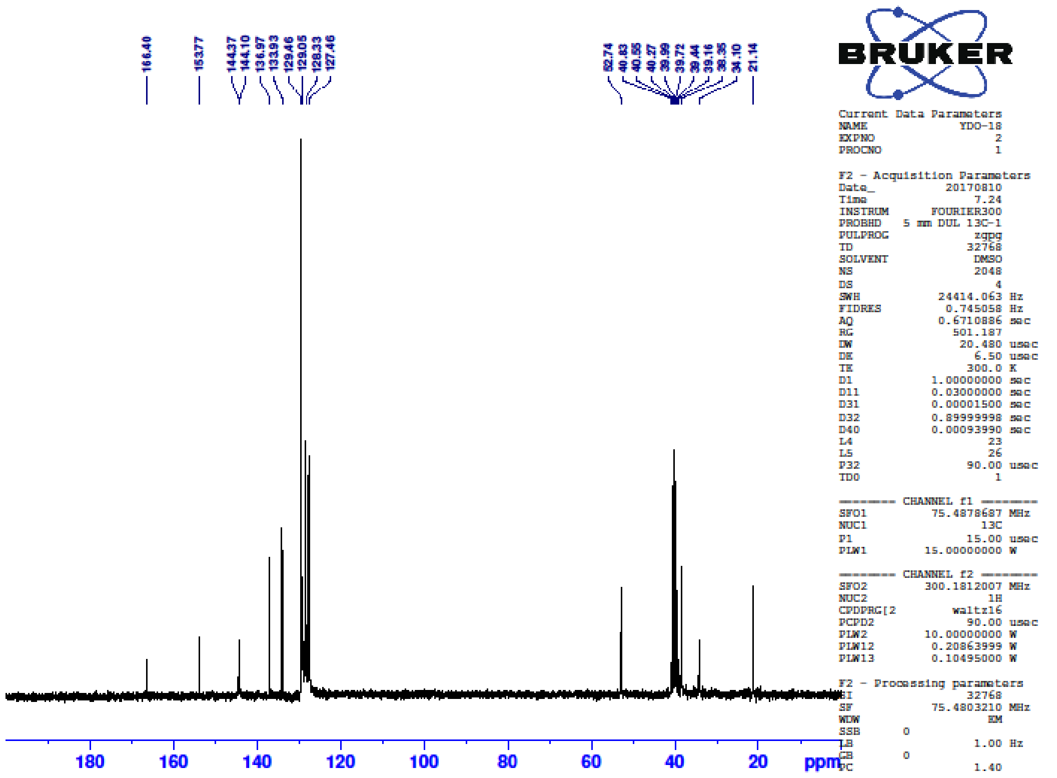

Spectra 56. ¹³C-NMR spectra of compound **4n**

DOPNALAB

Item	Value
Acquired Date&Time	3.05.2018 13:52:06
Acquired by	System Administrator
Filename	C:\Users\dopnalab\Desktop\derya\ydo sensi\ydo-191.ispd
Spectrum name	ydo-191
Sample name	ydo-19
Sample ID	
Option	
Comment	
No. of Scans	10
Resolution	4 [cm-1]
Apodization	Happ-Genzel

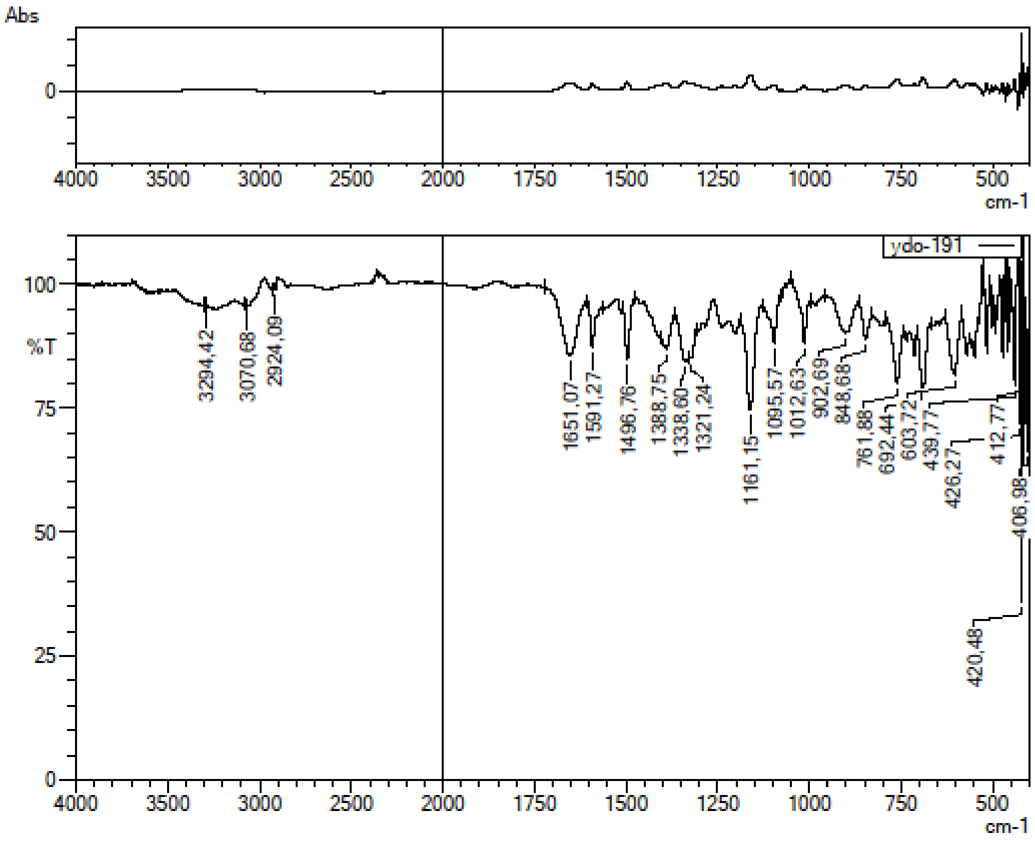

Spectra 57. IR spectra of compound **4o**

Data File: C:\LabSolutions\Data\Analiz\derya\YDO-19_36.lcd

Elmt	Val.	Min	Max	Elmt	Val.	Min	Max	Elmt	Val.	Min	Max	Elmt	Val.	Min	Max	Use Adduct
H	1	5	40	O	2	2	4	S	2	0	3	Ru	2	0	0	H
C	4	0	35	F	1	0	0	Cl	1	0	0	I	3	0	0	
N	3	3	6	P	3	0	0	Br	1	0	0					

Error Margin (ppm):	5	DBE Range:	6.0 - 20.0	Electron Ions:	both
HC Ratio:	unlimited	Apply N Rule:	yes	Use MSn Info:	yes
Max Isotopes:	3	Isotope RI (%):	1.00	Isotope Res:	9000
MSn Iso RI (%):	10.00	MSn Logic Mode:	AND	Max Results:	500

Event#: 1 MS(E+) Ret. Time : 6.653 Scan# : 999

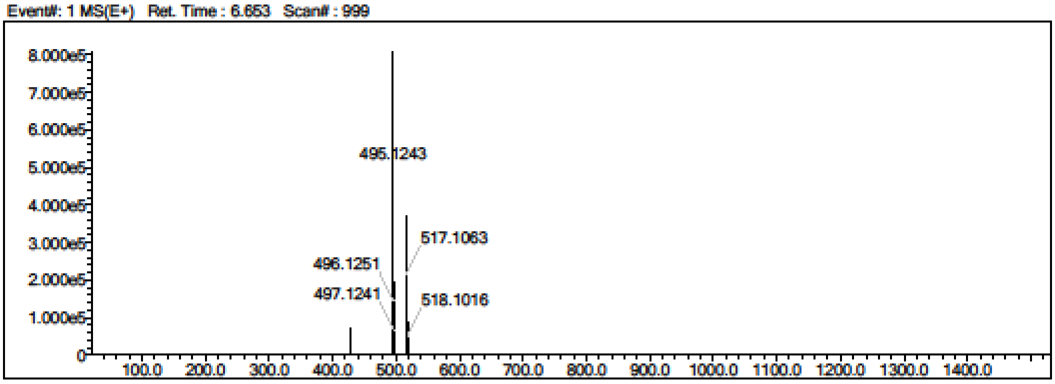

Measured region for 495.1243 m/z

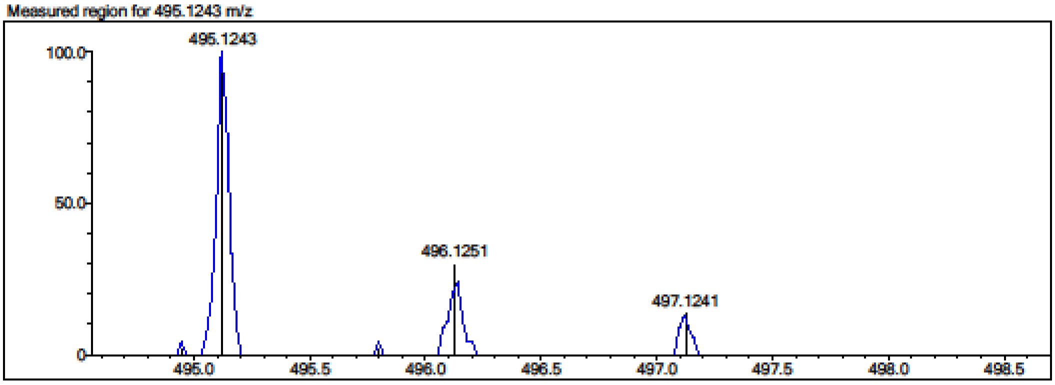

C23 H22 N6 O3 S2 [M+H]+ : Predicted region for 495.1268 m/z

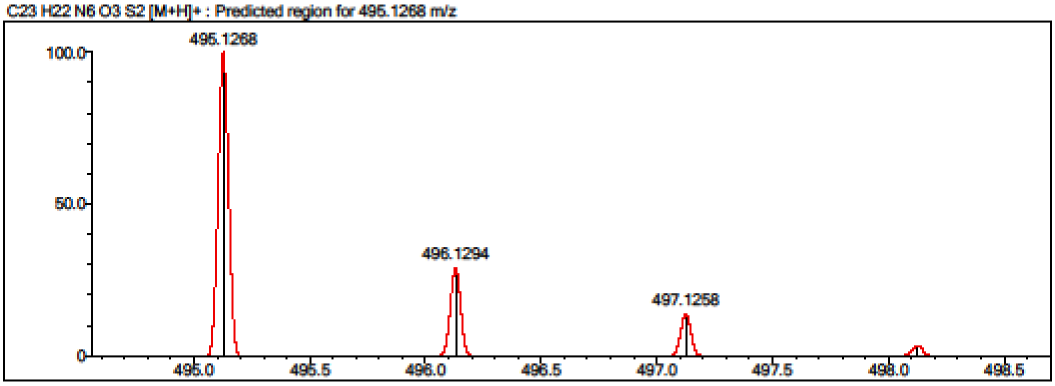

Rank	Score	Formula (M)	Ion	Meas. m/z	Pred. m/z	Df. (mDa)	Df. (ppm)	Iso	DBE
1	57.18	C23 H22 N6 O3 S2	[M+H]+	495.1243	495.1268	-2.5	-5.05	63.89	16.0

Spectra 58. HRMS spectra of compound **4o**

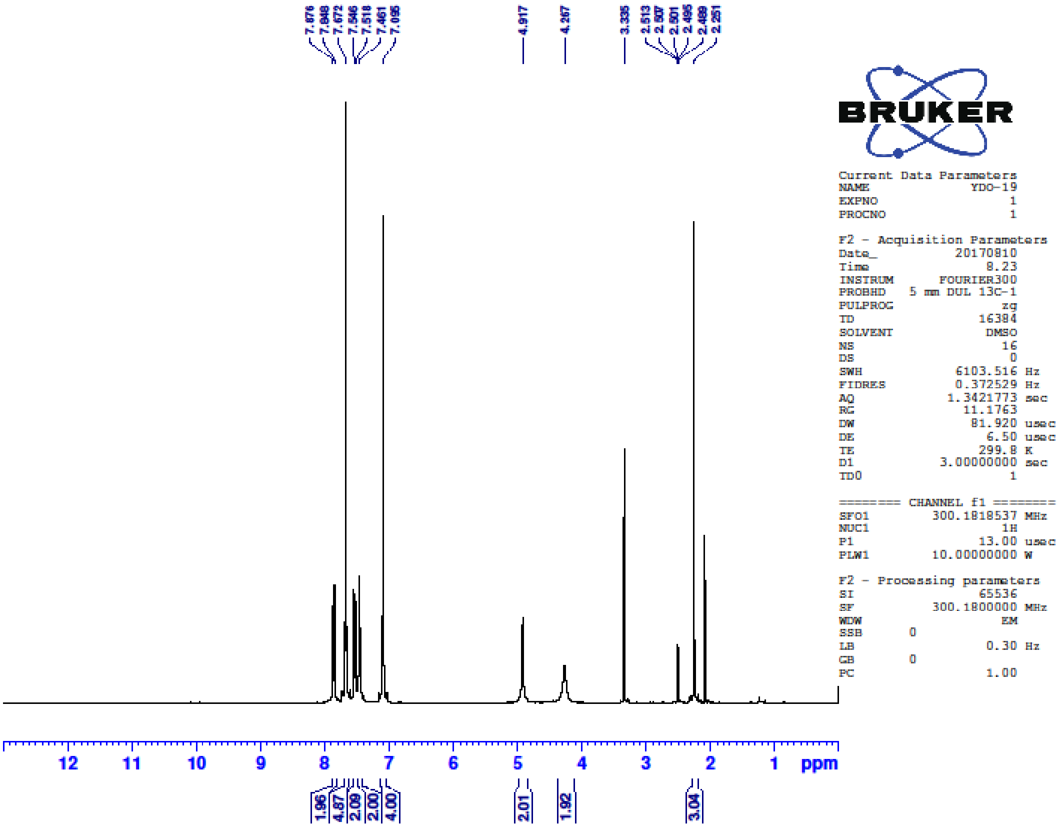

Spectra 59. ^{1}H-NMR spectra of compound **4o**

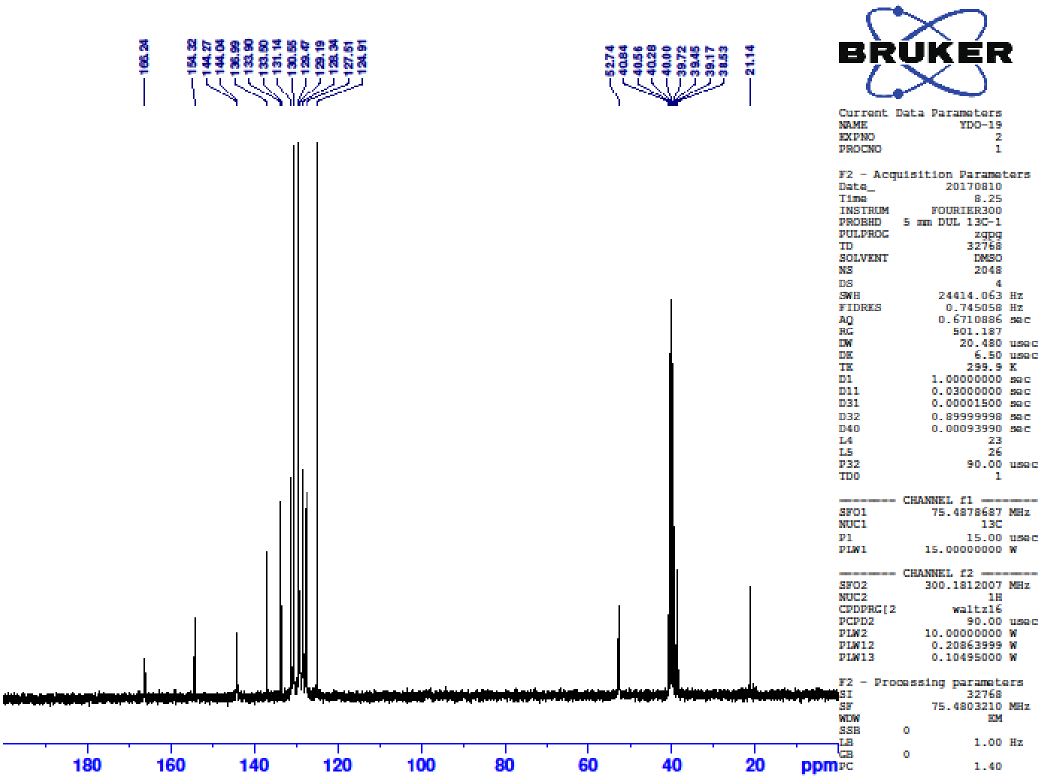

Spectra 60. ^{13}C-NMR spectra of compound **4o**

DOPNALAB

	Item	Value
	Acquired Date&Time	3.05.2018 13:54:40
	Acquired by	System Administrator
	Filename	C:\Users\dopnalab\Desktop\derya\ydo serisi\ydo-201.ispd
	Spectrum name	ydo-201
	Sample name	ydo-20
	Sample ID	
	Option	
	Comment	
	No. of Scans	10
	Resolution	4 [cm-1]
	Apodization	Happ-Genzel

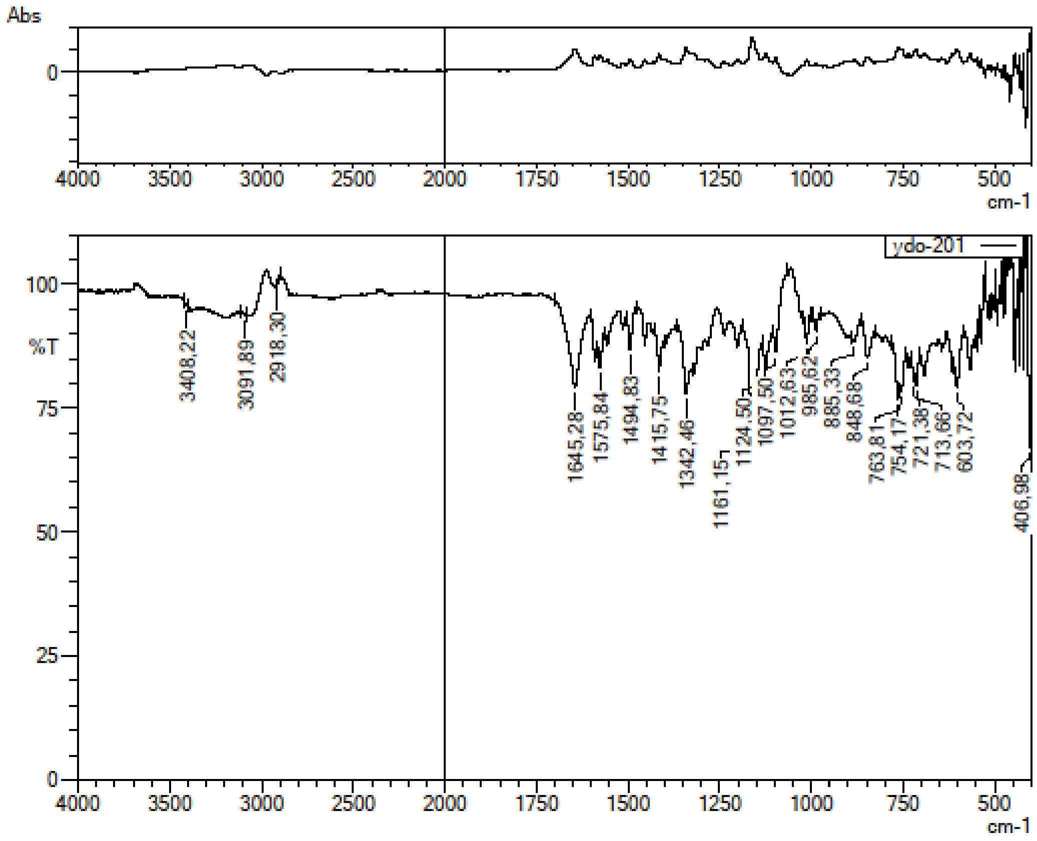

Spectra 61. IR spectra of compound **4p**

Data File: C:\LabSolutions\Data\Analiz\derya\YDO-20_37.lcd

Elmt	Val.	Min	Max	Elmt	Val.	Min	Max	Elmt	Val.	Min	Max	Elmt	Val.	Min	Max	Use Adduct
H	1	5	40	O	2	2	4	S	2	0	3	Ru	2	0	0	H
C	4	0	35	F	1	0	0	Cl	1	0	0	I	3	0	0	
N	3	3	6	P	3	0	0	Br	1	0	0					

Error Margin (ppm):	5	DBE Range:	6.0 - 20.0	Electron Ions:	both
HC Ratio:	unlimited	Apply N Rule:	yes	Use MSn Info:	yes
Max Isotopes:	3	Isotope RI (%):	1.00	Isotope Res:	9000
MSn Iso RI (%):	10.00	MSn Logic Mode:	AND	Max Results:	500

Event#: 1 MS(E+) Ret. Time : 5.973 -> 6.107 Scan# : 897 -> 917

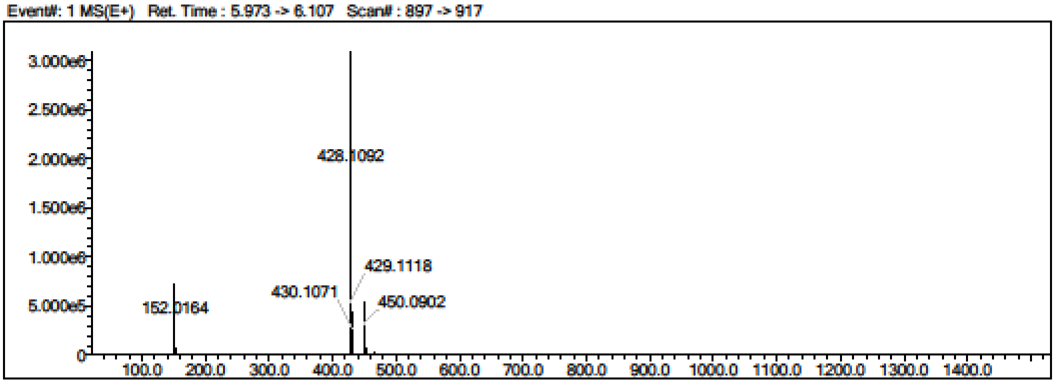

Measured region for 428.1092 m/z

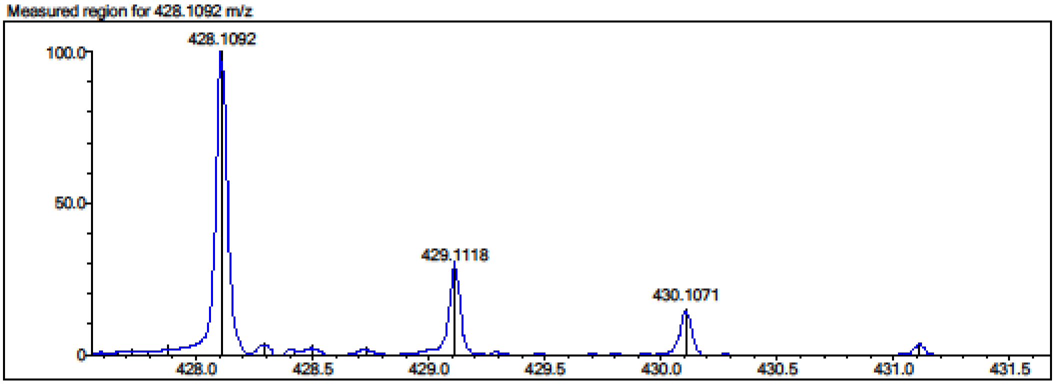

C21 H21 N3 O3 S2 [M+H]+ : Predicted region for 428.1097 m/z

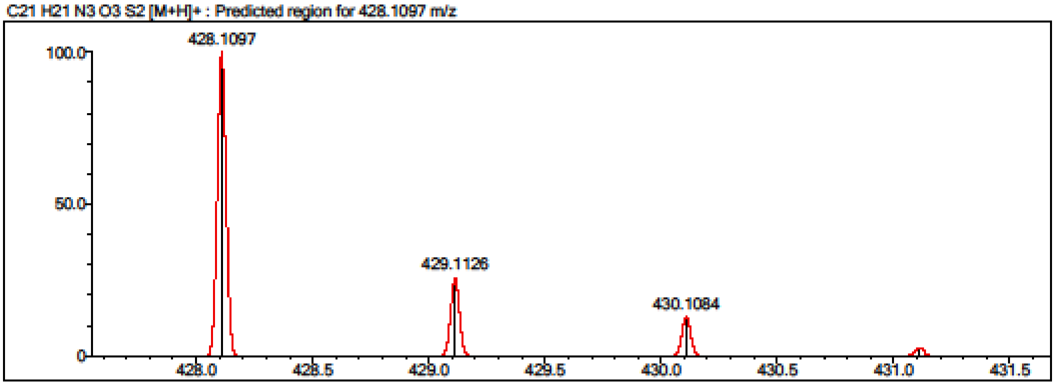

Rank	Score	Formula (M)	Ion	Meas. m/z	Pred. m/z	Df. (mDa)	Df. (ppm)	Iso	DBE
1	93.19	C21 H21 N3 O3 S2	[M+H]+	428.1092	428.1097	-0.5	-1.17	93.59	13.0

Spectra 62. HRMS spectra of compound **4p**

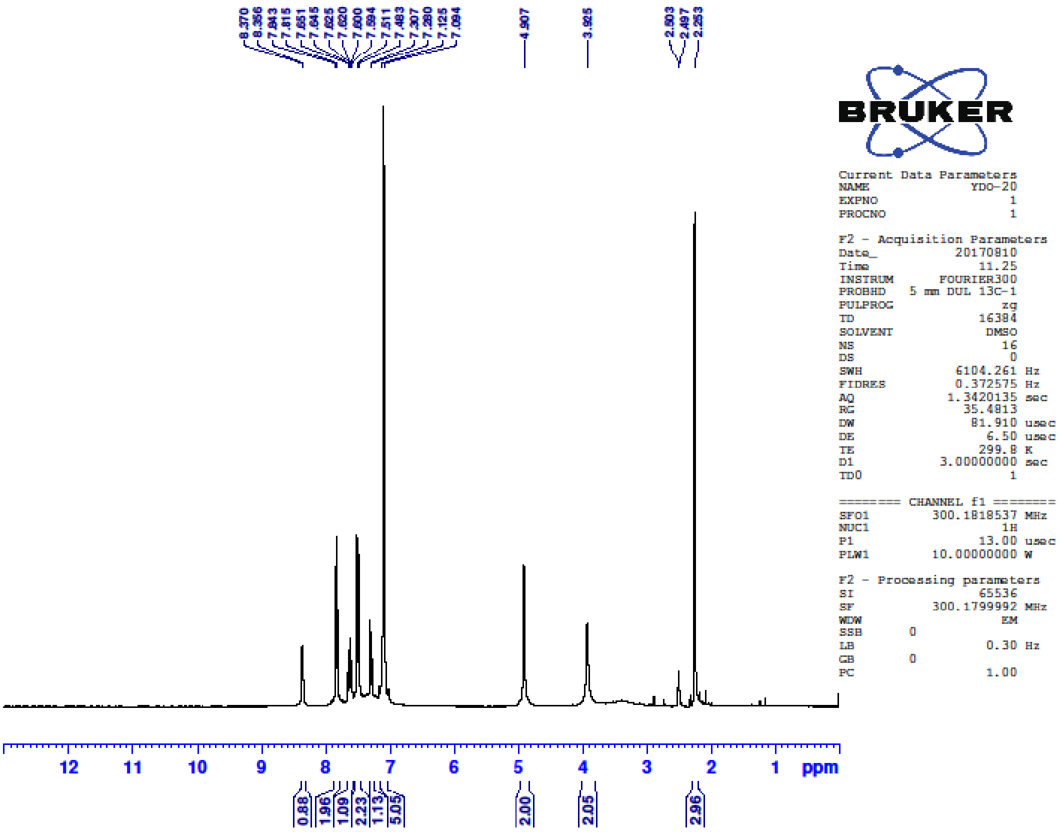

Spectra 63. ^{1}H-NMR spectra of compound **4p**

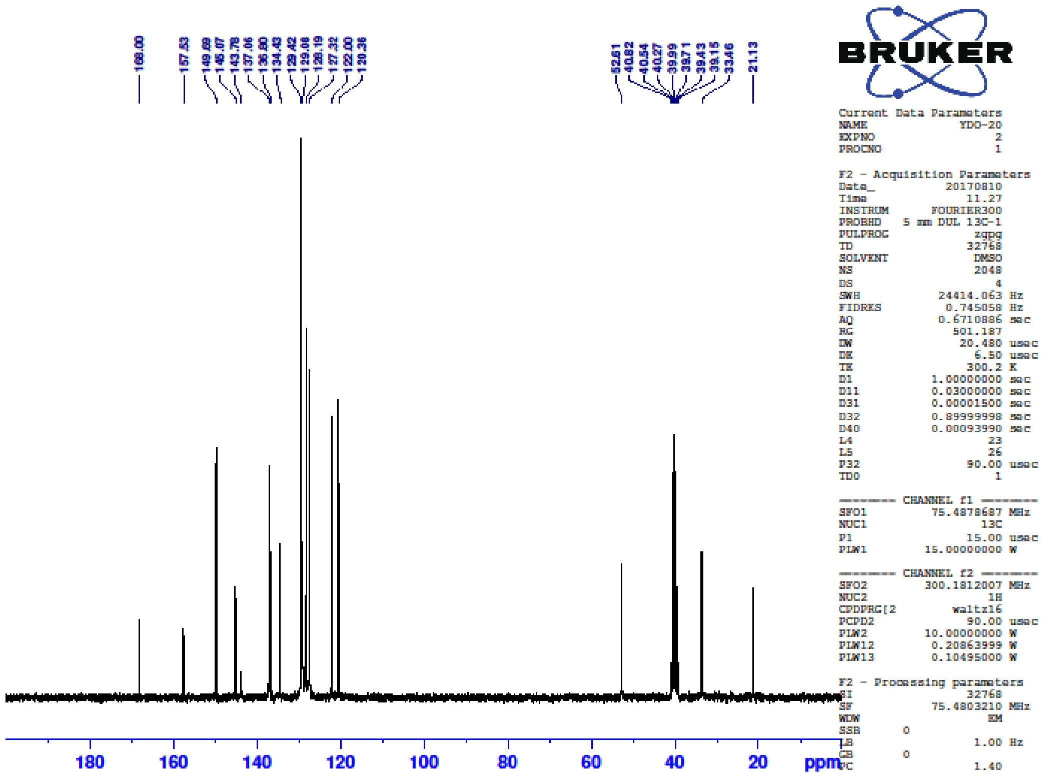

Spectra 64. ^{13}C-NMR spectra of compound **4p**

DOPNALAB

	Item	Value
	Acquired Date&Time	3.05.2018 13:57:14
	Acquired by	System Administrator
	Filename	C:\Users\dopnalab\Desktop\derya\ydo serisi\ydo-221.ispd
	Spectrum name	ydo-221
	Sample name	ydo-22
	Sample ID	
	Option	
	Comment	
	No. of Scans	10
	Resolution	4 [cm-1]
	Apodization	Happ-Genzel

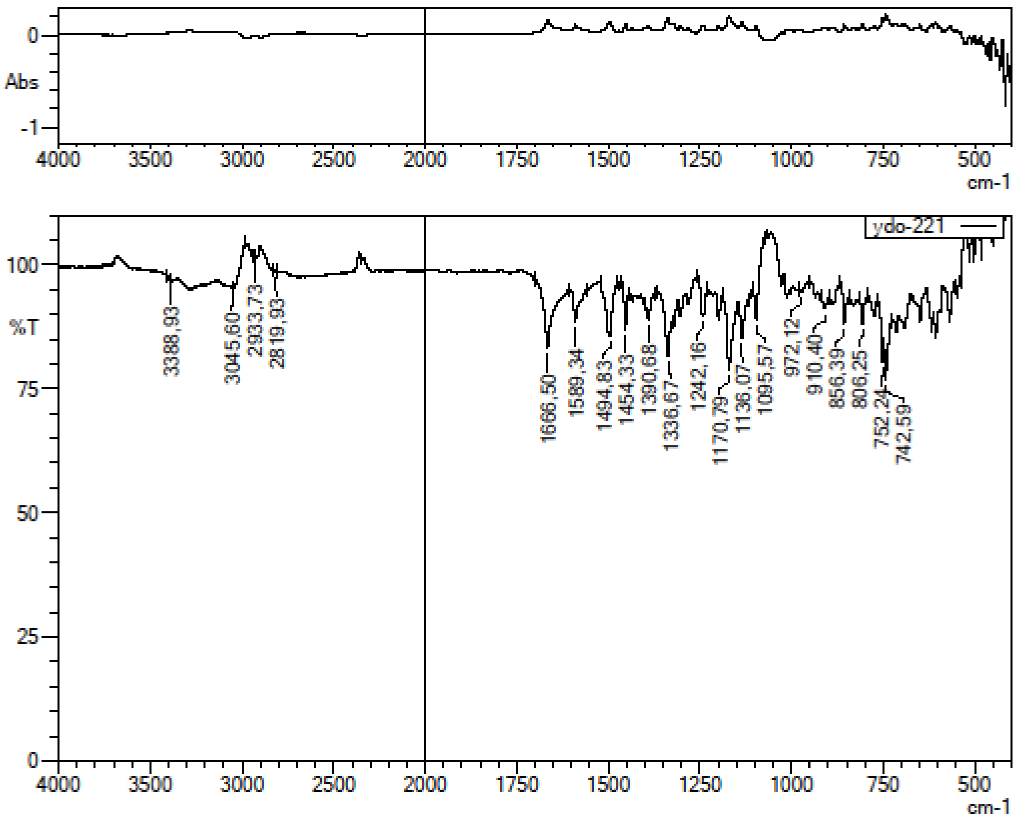

Spectra 65. IR spectra of compound **4r**

Data File: C:\LabSolutions\Data\Analiz\derya\YDO-22_39.lcd

Elmt	Val.	Min	Max	Elmt	Val.	Min	Max	Elmt	Val.	Min	Max	Elmt	Val.	Min	Max	Use Adduct
H	1	5	40	O	2	2	4	S	2	0	3	Ru	2	0	0	H
C	4	0	35	F	1	0	0	Cl	1	0	0	I	3	0	0	
N	3	3	6	P	3	0	0	Br	1	0	0					

Error Margin (ppm):	5	DBE Range:	6.0 - 19.0	Electron Ions:	both
HC Ratio:	unlimited	Apply N Rule:	yes	Use MSn Info:	yes
Max Isotopes:	3	Isotope RI (%):	1.00	Isotope Res:	9000
MSn Iso RI (%):	10.00	MSn Logic Mode:	AND	Max Results:	500

Event#: 1 MS(E+) Ret. Time : 6.573 -> 6.853 Scan# : 987 -> 1029

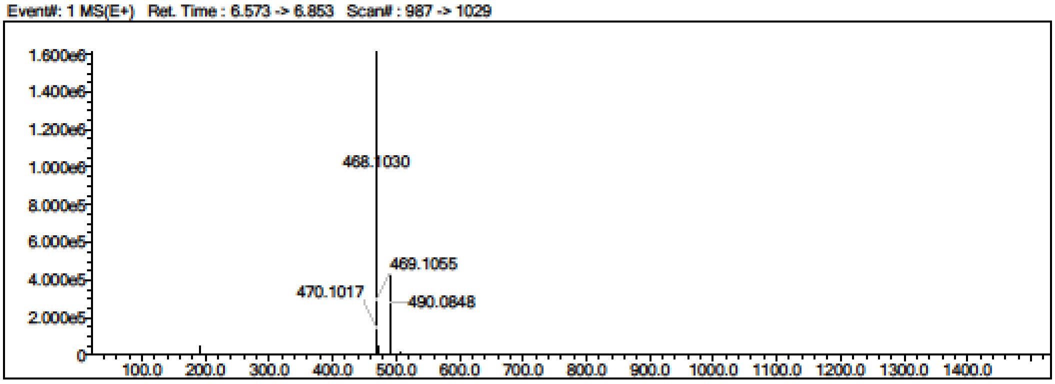

Measured region for 468.1030 m/z

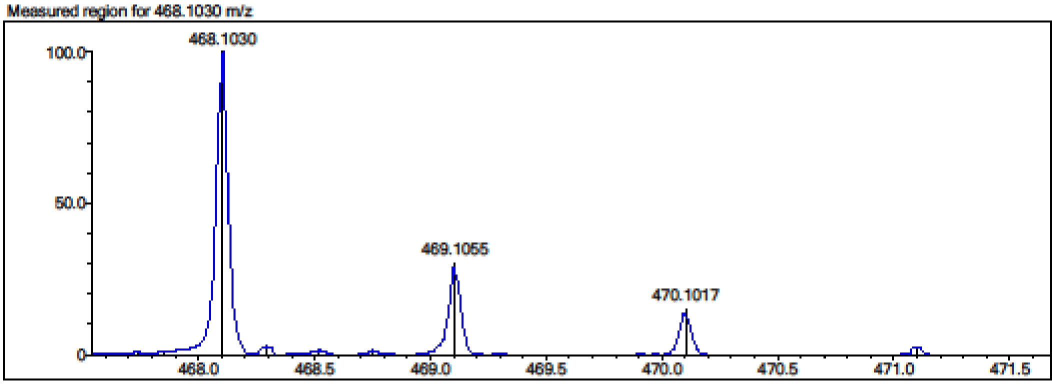

C23 H21 N3 O4 S2 [M+H]+ : Predicted region for 468.1046 m/z

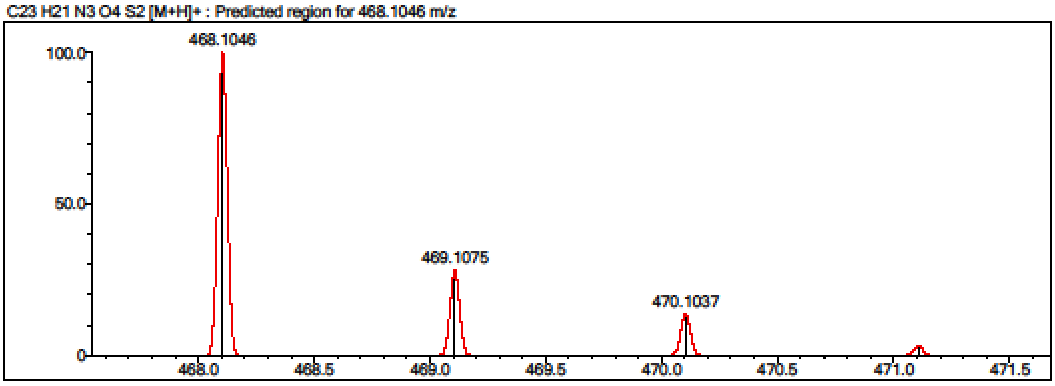

Rank	Score	Formula (M)	Ion	Meas. m/z	Pred. m/z	Df. (mDa)	Df. (ppm)	Iso	DBE
1	87.21	C23 H21 N3 O4 S2	[M+H]+	468.1030	468.1046	-1.6	-3.42	92.83	15.0

Spectra 66. HRMS spectra of compound **4r**

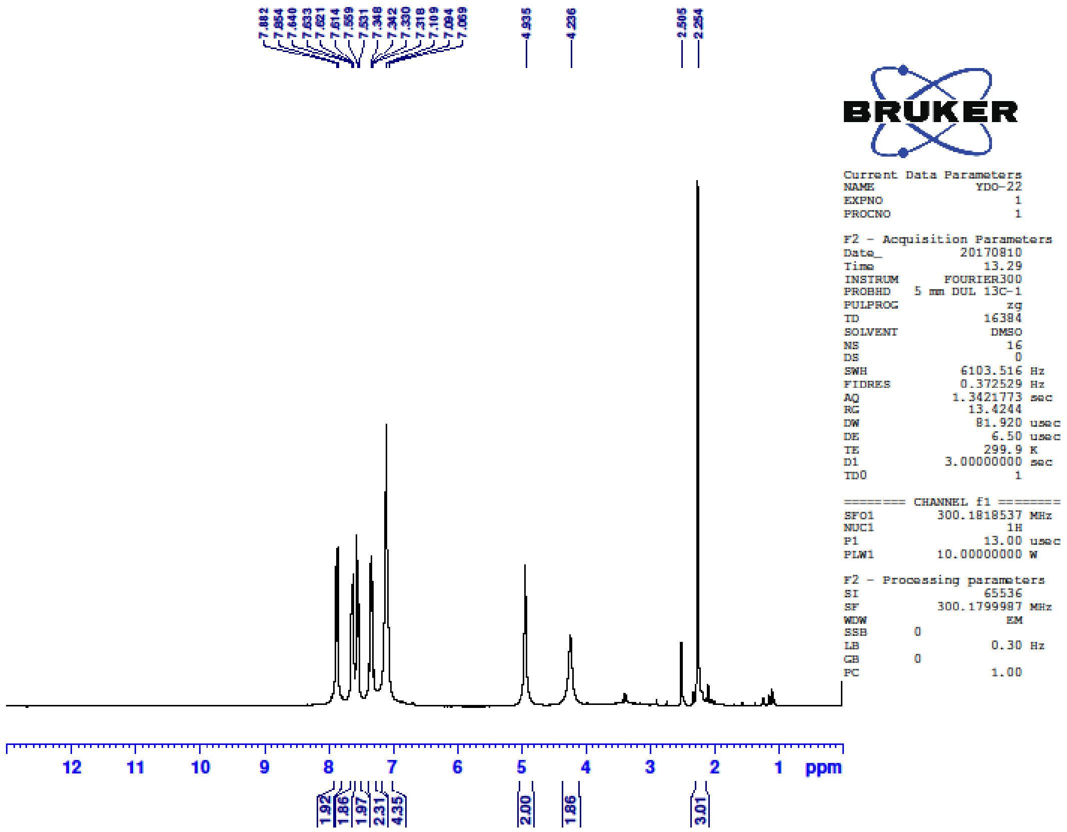

Spectra 67. ^{1}H-NMR spectra of compound **4r**

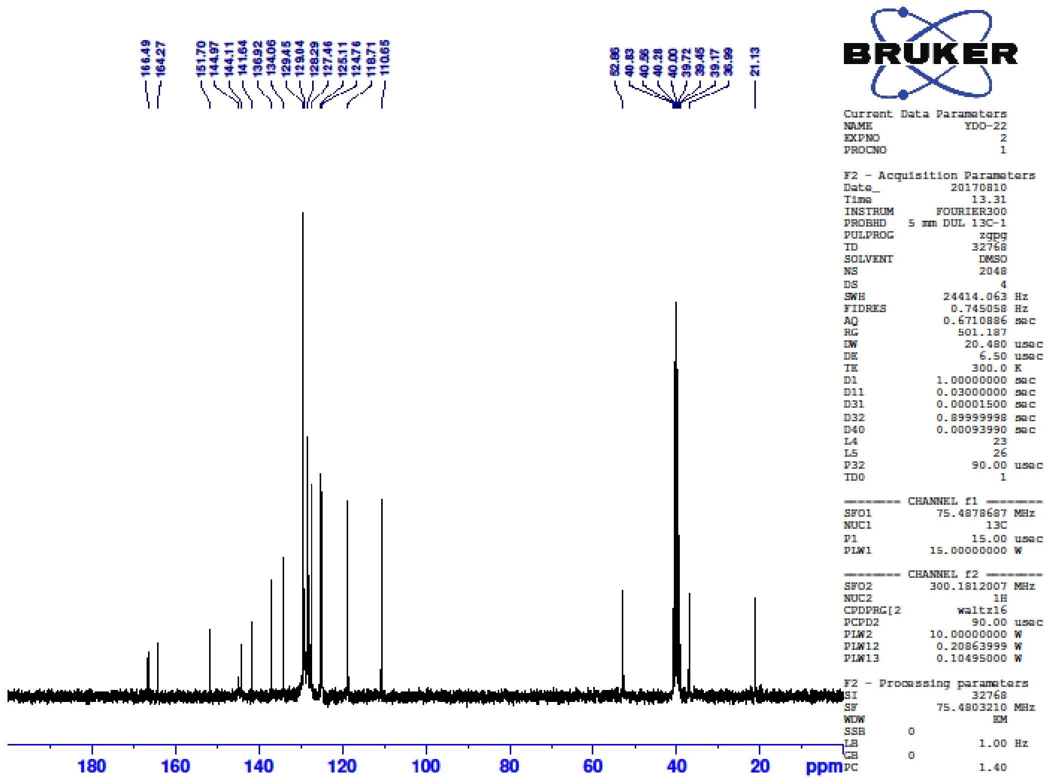

Spectra 68. ^{13}C-NMR spectra of compound **4r**

DOPNALAB

	Item	Value
	Acquired Date&Time	3.05.2018 14:01:30
	Acquired by	System Administrator
	Filename	C:\Users\dopnalab\Desktop\derya\ydo sensi\ydo-231.ispd
	Spectrum name	ydo-231
	Sample name	ydo-23
	Sample ID	
	Option	
	Comment	
	No. of Scans	10
	Resolution	4 [cm-1]
	Apodization	Happ-Genzel

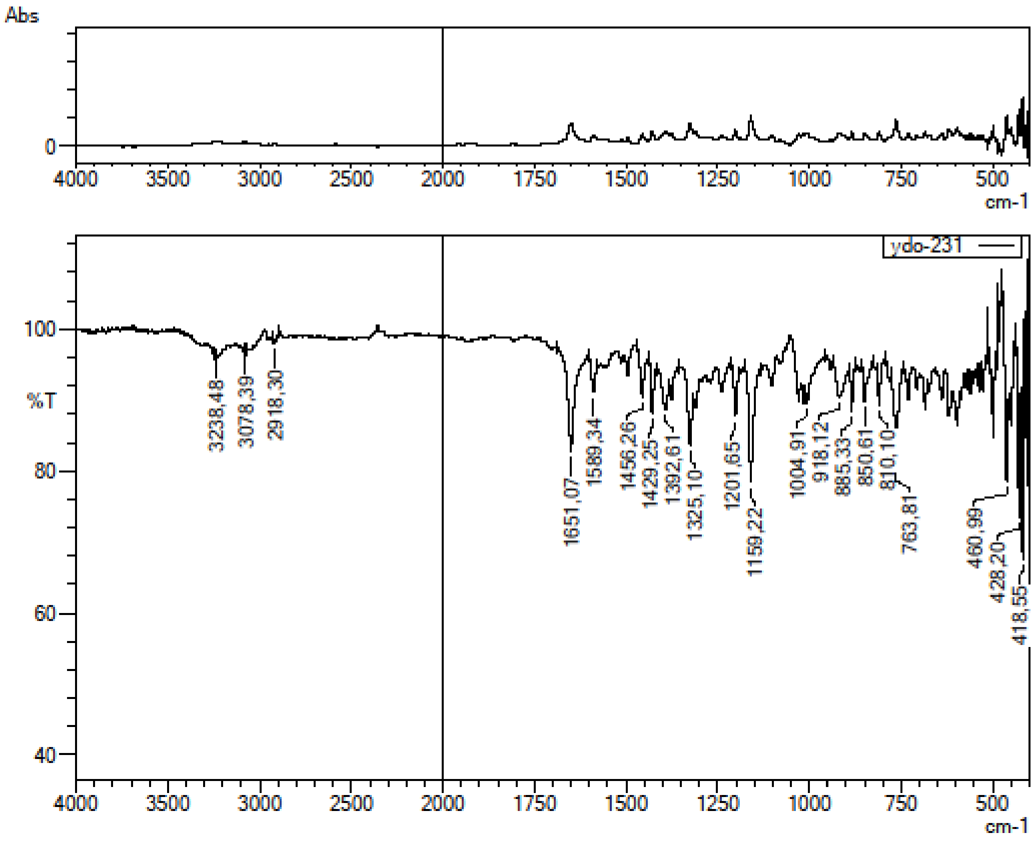

Spectra 69. IR spectra of compound **4s**

Formula Predictor Report - YDO-23_40.lcd

Data File: C:\LabSolutions\Data\Analiz\derya\YDO-23_40.lcd

Elmt	Val.	Min	Max	Elmt	Val.	Min	Max	Elmt	Val.	Min	Max	Elmt	Val.	Min	Max	Use Adduct
H	1	5	40	O	2	2	4	S	2	0	3	Ru	2	0	0	H
C	4	0	35	F	1	0	0	Cl	1	0	0	I	3	0	0	
N	3	3	6	P	3	0	0	Br	1	0	0					

Error Margin (ppm): 7
HC Ratio: unlimited
Max Isotopes: 3
MSn Iso RI (%): 10.00

DBE Range: 6.0 - 19.0
Apply N Rule: yes
Isotope RI (%): 1.00
MSn Logic Mode: AND

Electron Ions: both
Use MSn Info: yes
Isotope Res: 9000
Max Results: 500

Event#: 1 MS(E+) Ret. Time : 6.867 -> 7.093 Scan# : 1031 -> 1065

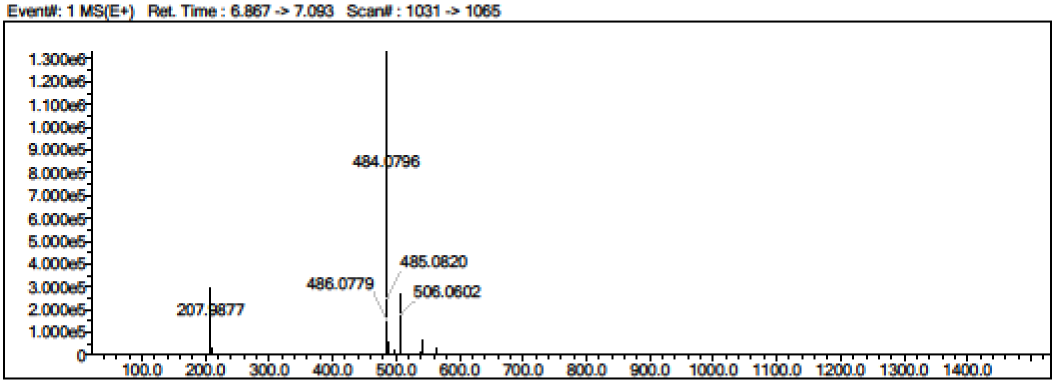

Measured region for 484.0796 m/z

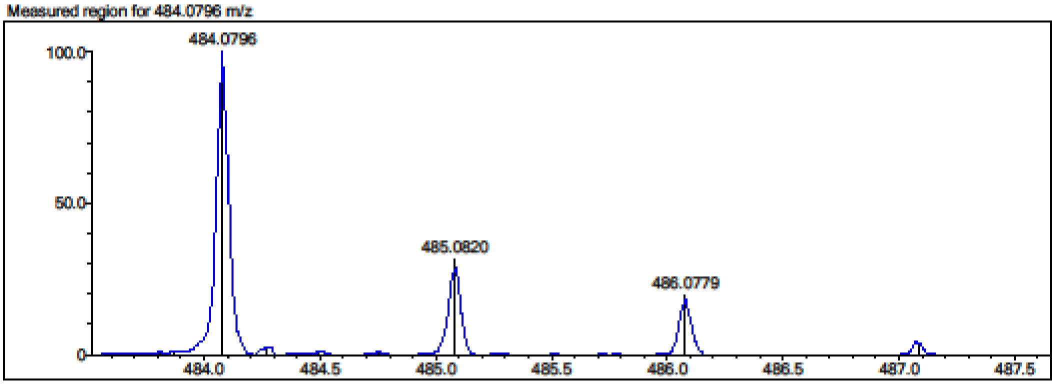

C23 H21 N3 O3 S3 [M+H]+ : Predicted region for 484.0818 m/z

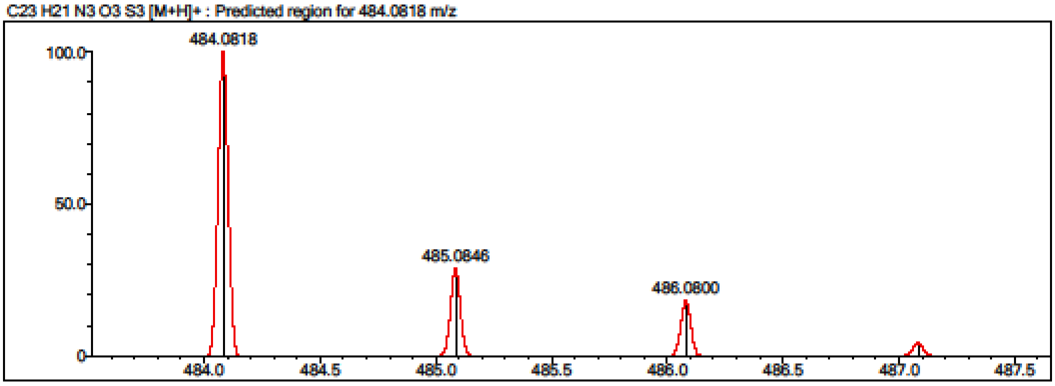

Rank	Score	Formula (M)	Ion	Meas. m/z	Pred. m/z	Df. (mDa)	Df. (ppm)	Iso	DBE
1	90.40	C23 H21 N3 O3 S3	[M+H]+	484.0796	484.0818	-2.2	-4.54	99.18	15.0

Spectra 70. HRMS spectra of compound **4s**

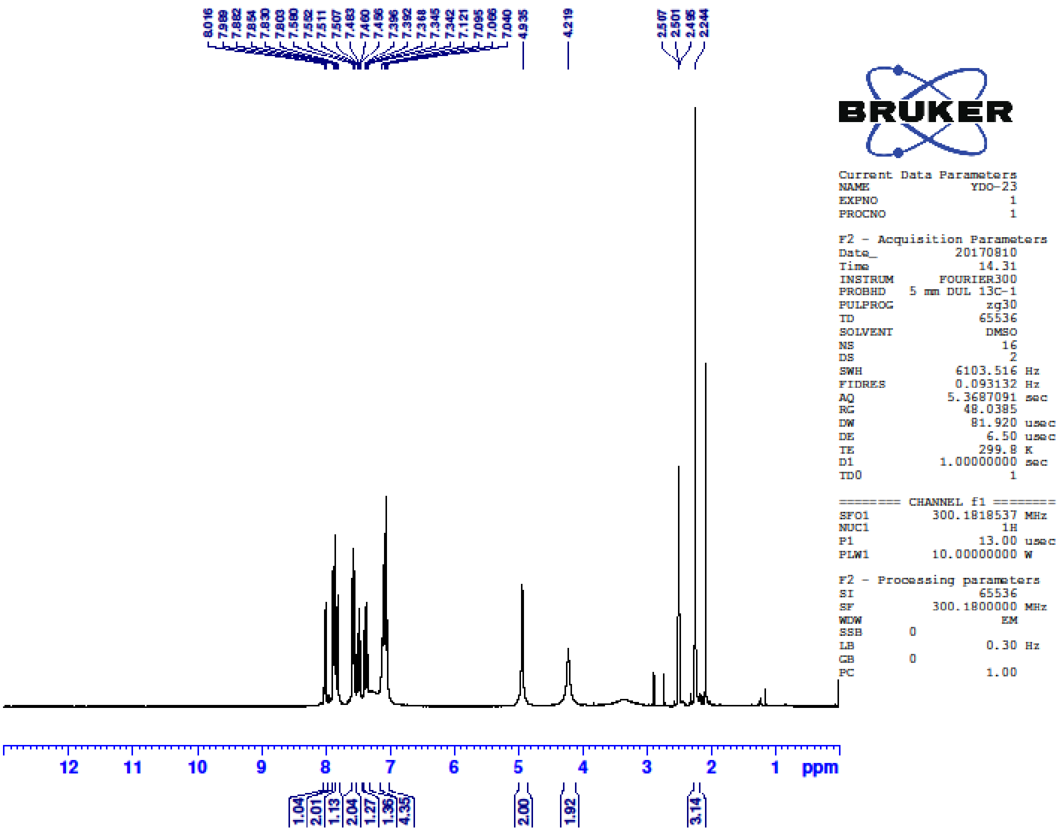

Spectra 71. ^{1}H-NMR spectra of compound **4s**

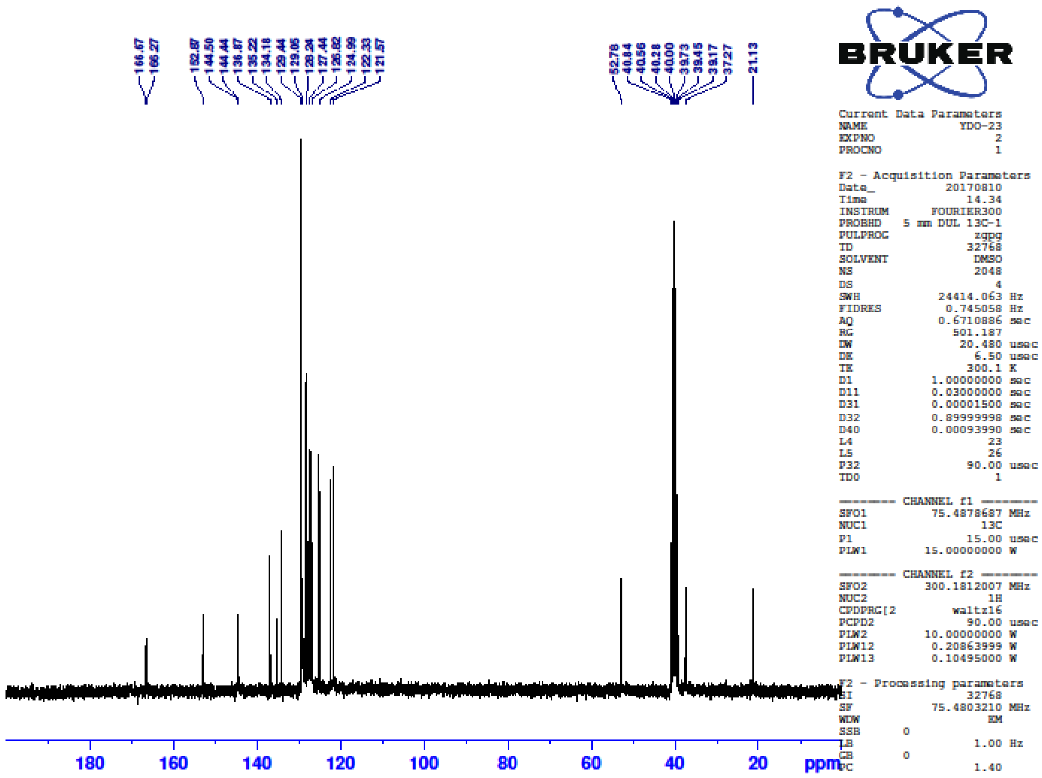

Spectra 72. ^{13}C-NMR spectra of compound **4s**

DOPNALAB

Item	Value
Acquired Date&Time	3.05.2018 14:03:28
Acquired by	System Administrator
Filename	C:\Users\dopnalab\Desktop\derya\ydo serisi\ydo-241.ispd
Spectrum name	ydo-241
Sample name	ydo-24
Sample ID	
Option	
Comment	
No. of Scans	10
Resolution	4 [cm-1]
Apodization	Happ-Genzel

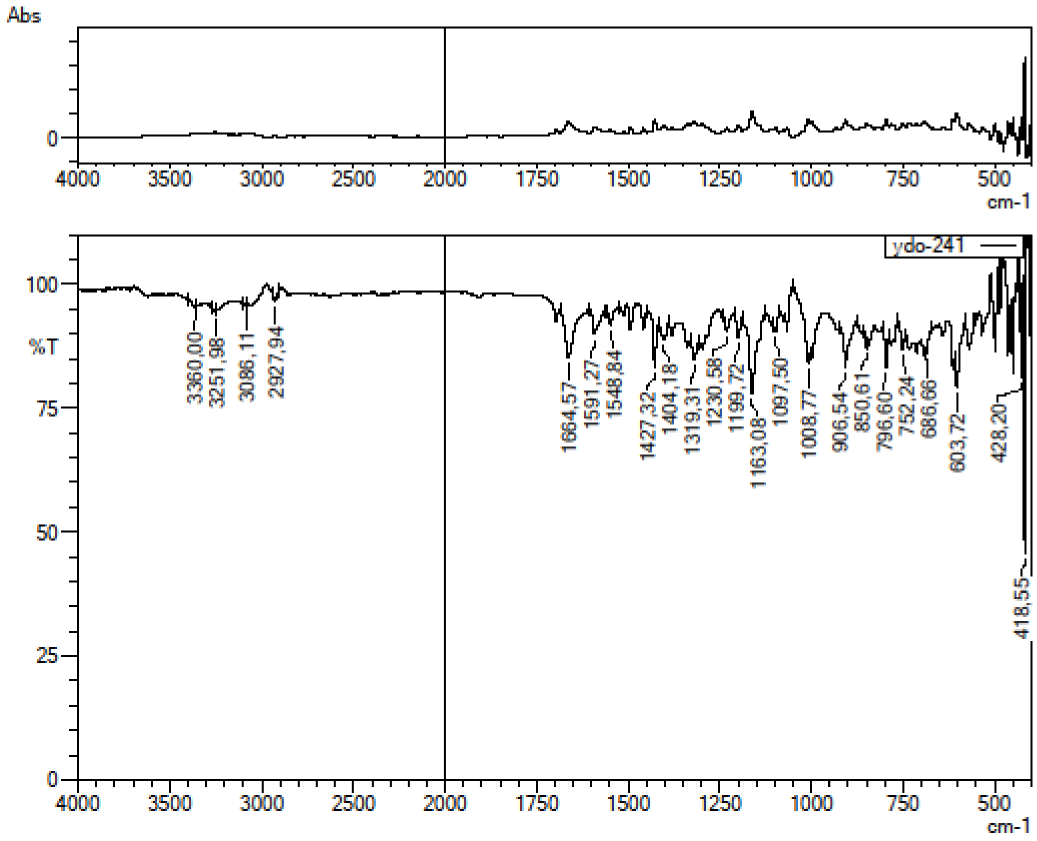

Spectra 73. IR spectra of compound **4t**

Data File: C:\LabSolutions\Data\Analiz\derya\YDO-24_41.lcd

Elmt	Val.	Min	Max	Elmt	Val.	Min	Max	Elmt	Val.	Min	Max	Elmt	Val.	Min	Max	Use Adduct
H	1	5	40	O	2	2	4	S	2	0	3	Ru	2	0	0	H
C	4	0	35	F	1	0	0	Cl	1	0	1	I	3	0	0	
N	3	3	6	P	3	0	0	Br	1	0	0					

Error Margin (ppm): 7
HC Ratio: unlimited
Max Isotopes: 3
MSn Iso RI (%): 10.00

DBE Range: 6.0 - 19.0
Apply N Rule: yes
Isotope RI (%): 1.00
MSn Logic Mode: AND

Electron Ions: both
Use MSn Info: yes
Isotope Res: 9000
Max Results: 500

Event#: 1 MS(E+) Ret. Time : 7.093 -> 7.240 Scan# : 1065 -> 1087

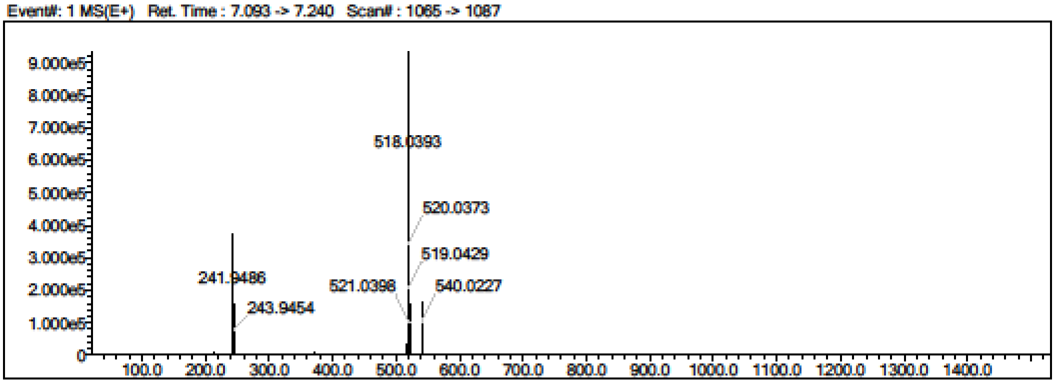

Measured region for 518.0393 m/z

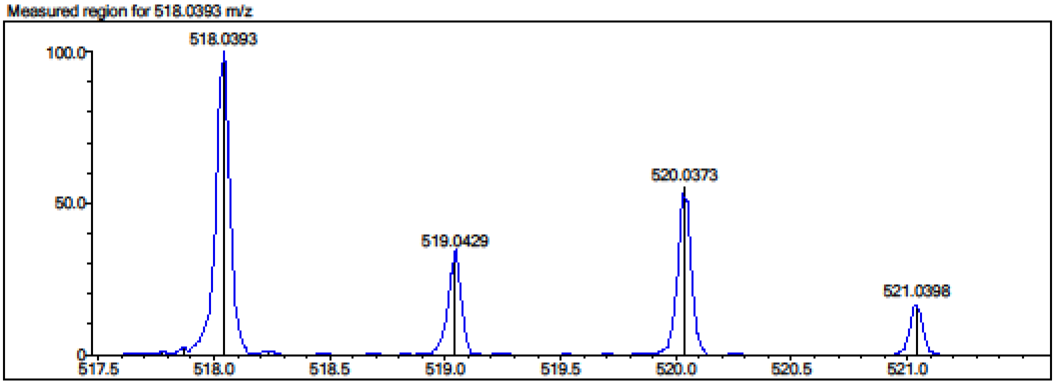

C23 H20 N3 O3 S3 Cl [M+H]+ : Predicted region for 518.0428 m/z

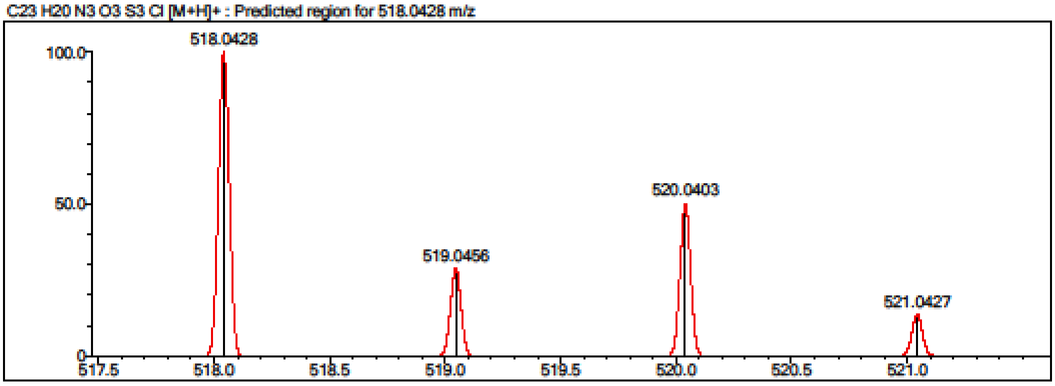

Rank	Score	Formula (M)	Ion	Meas. m/z	Pred. m/z	Df. (mDa)	Df. (ppm)	Iso	DBE
1	63.39	C23 H20 N3 O3 S3 Cl	[M+H]+	518.0393	518.0428	-3.5	-6.76	87.56	15.0

Spectra 74. HRMS spectra of compound **4t**

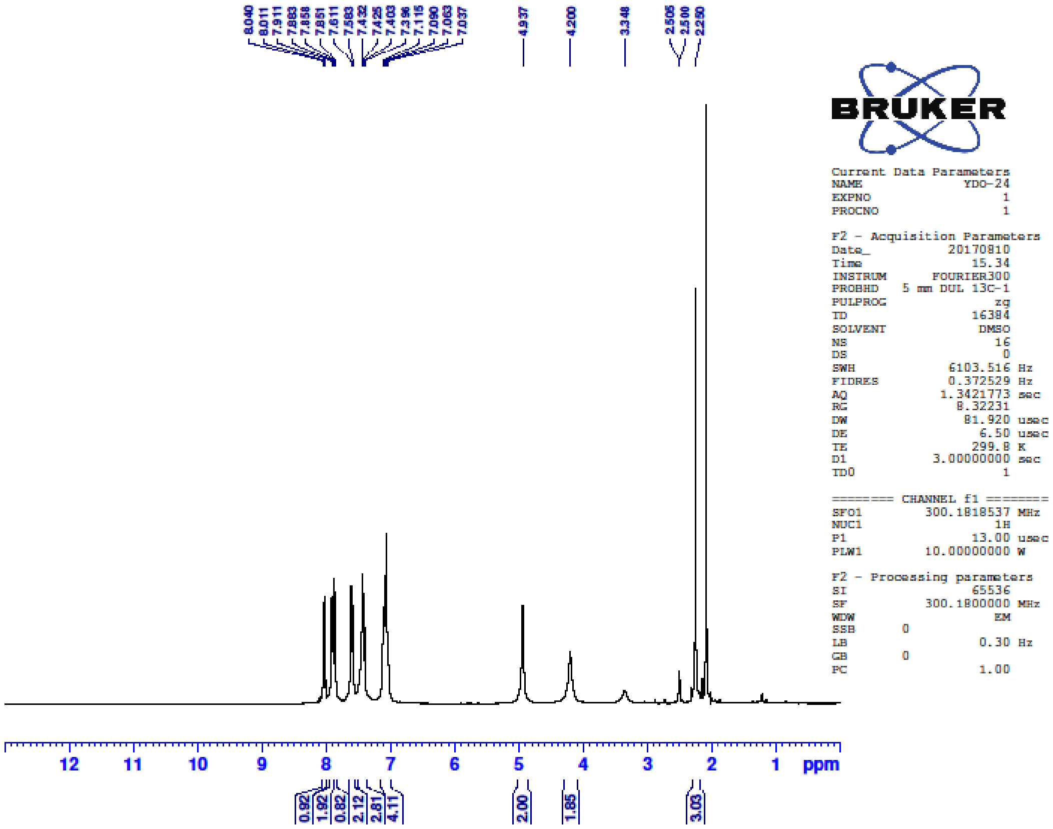

Spectra 75. ^{1}H-NMR spectra of compound **4t**

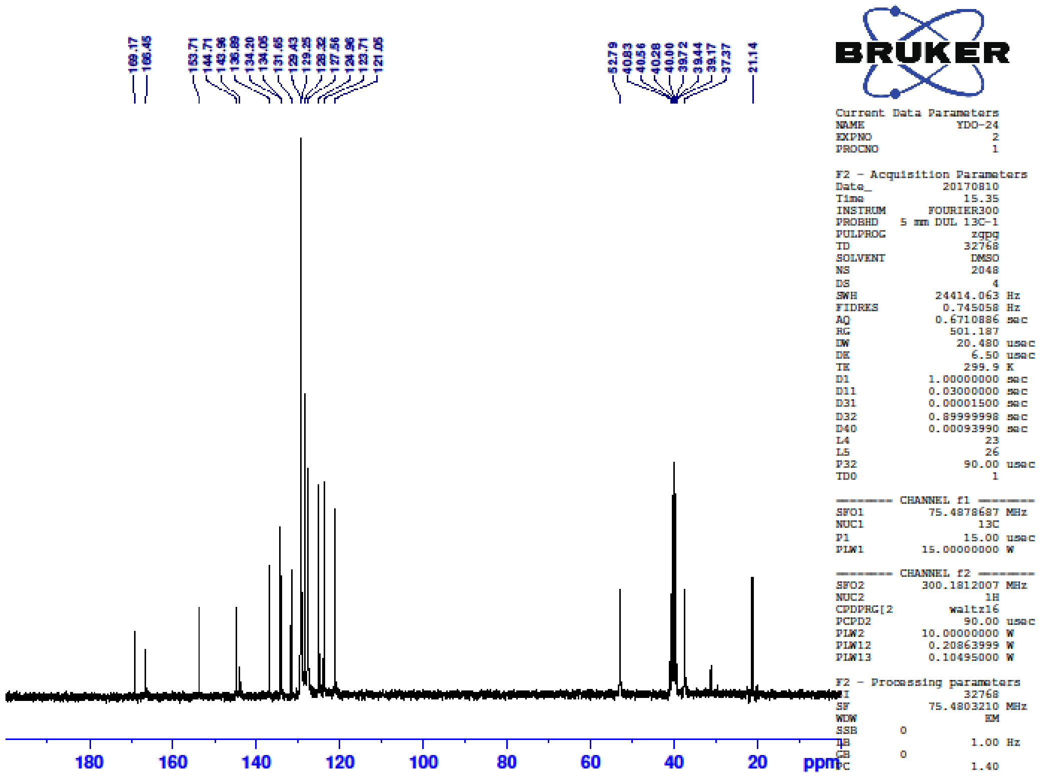

Spectra 76. ^{13}C-NMR spectra of compound **4t**

DOPNALAB

Item	Value
Acquired Date&Time	3.05.2018 14:06:00
Acquired by	System Administrator
Filename	C:\Users\dopnalab\Desktop\derya\ydo sensi\ydo-251.ispd
Spectrum name	ydo-251
Sample name	ydo-25
Sample ID	
Option	
Comment	
No. of Scans	10
Resolution	4 [cm-1]
Apodization	Happ-Genzel

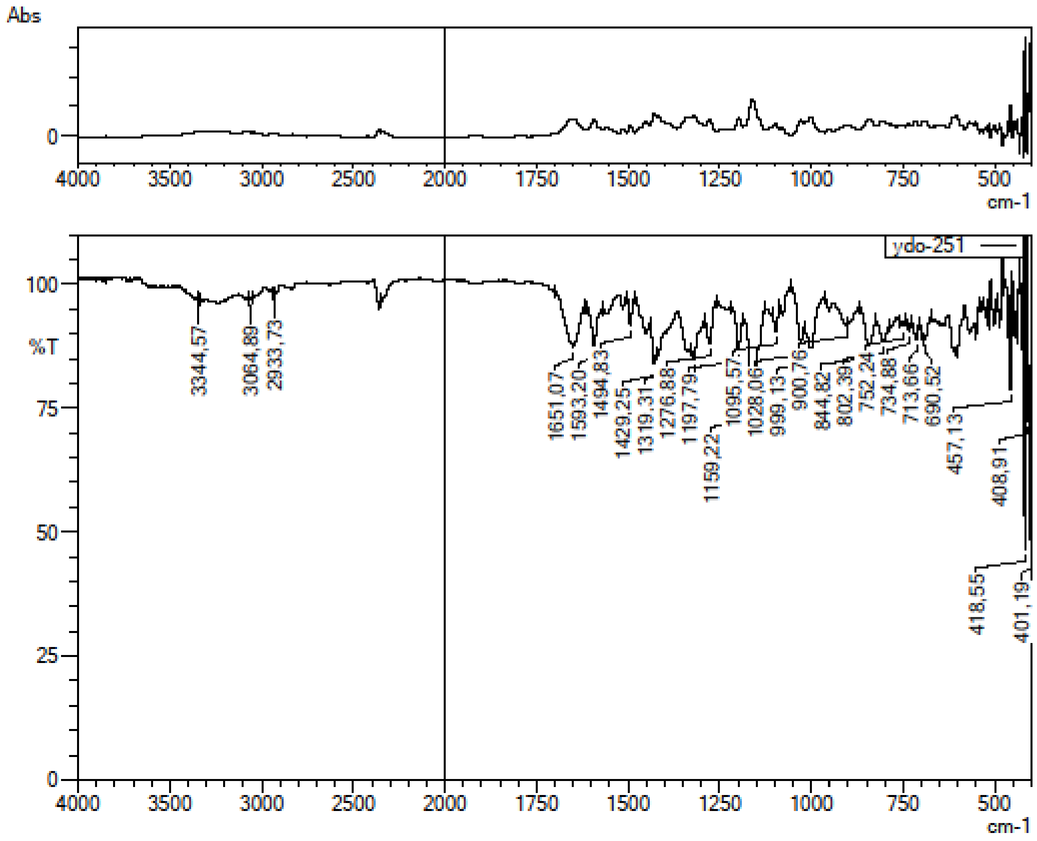

Spectra 77. IR spectra of compound **4u**

Formula Predictor Report - YDO-25_42.lcd Page 1 of 1

Data File: C:\LabSolutions\Data\Analiz\derya\YDO-25_42.lcd

Elmt	Val.	Min	Max	Elmt	Val.	Min	Max	Elmt	Val.	Min	Max	Elmt	Val.	Min	Max	Use Adduct
H	1	5	40	O	2	2	4	S	2	0	3	Ru	2	0	0	H
C	4	0	35	F	1	0	0	Cl	1	0	1	I	3	0	0	
N	3	3	6	P	3	0	0	Br	1	0	0					

Error Margin (ppm): 7
HC Ratio: unlimited
Max Isotopes: 3
MSn Iso RI (%): 10.00

DBE Range: 6.0 - 19.0
Apply N Rule: yes
Isotope RI (%): 1.00
MSn Logic Mode: AND

Electron Ions: both
Use MSn Info: yes
Isotope Res: 9000
Max Results: 500

Event#: 1 MS(E+) Ret. Time : 6.773 -> 6.960 Scan# : 1017 -> 1045

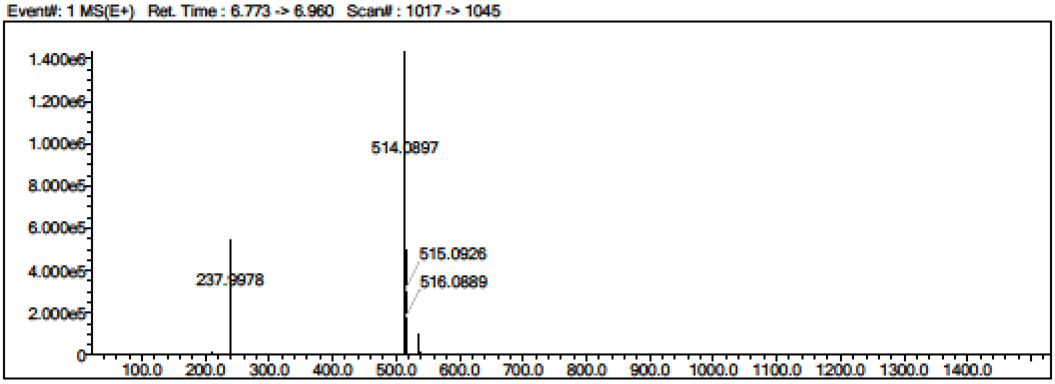

Measured region for 514.0897 m/z

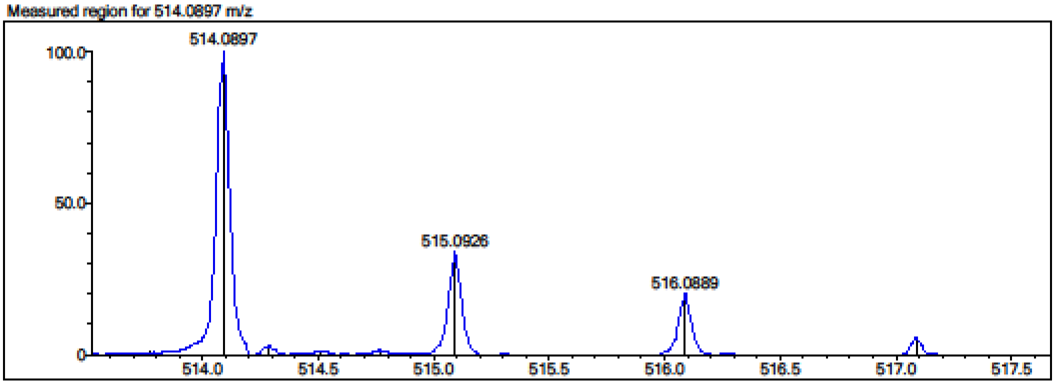

C24 H23 N3 O4 S3 [M+H]+ : Predicted region for 514.0923 m/z

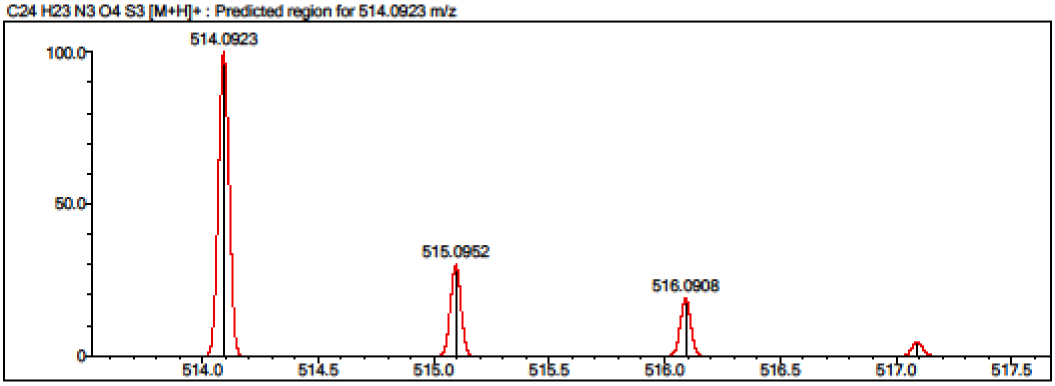

Rank	Score	Formula (M)	Ion	Meas. m/z	Pred. m/z	Df. (mDa)	Df. (ppm)	Iso	DBE
1	89.40	C24 H23 N3 O4 S3	[M+H]+	514.0897	514.0923	-2.6	-5.06	100.00	15.0

Spectra 78. HRMS spectra of compound **4u**

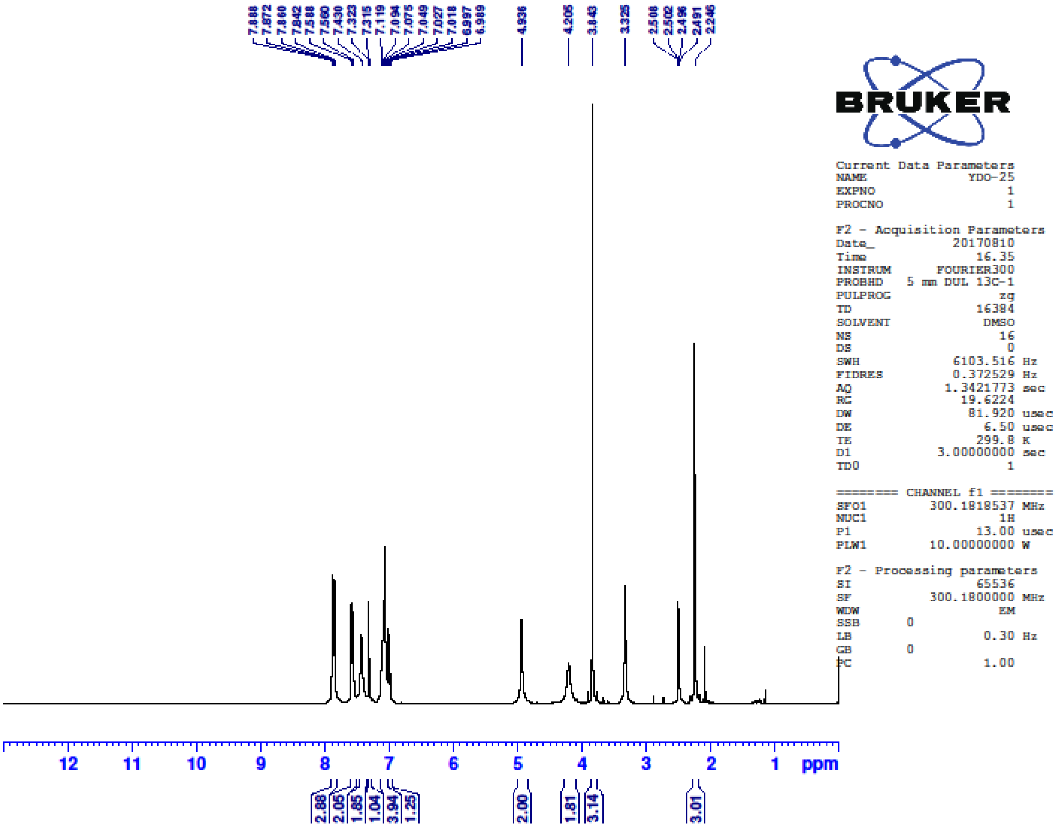

Spectra 79. ^{1}H-NMR spectra of compound **4u**

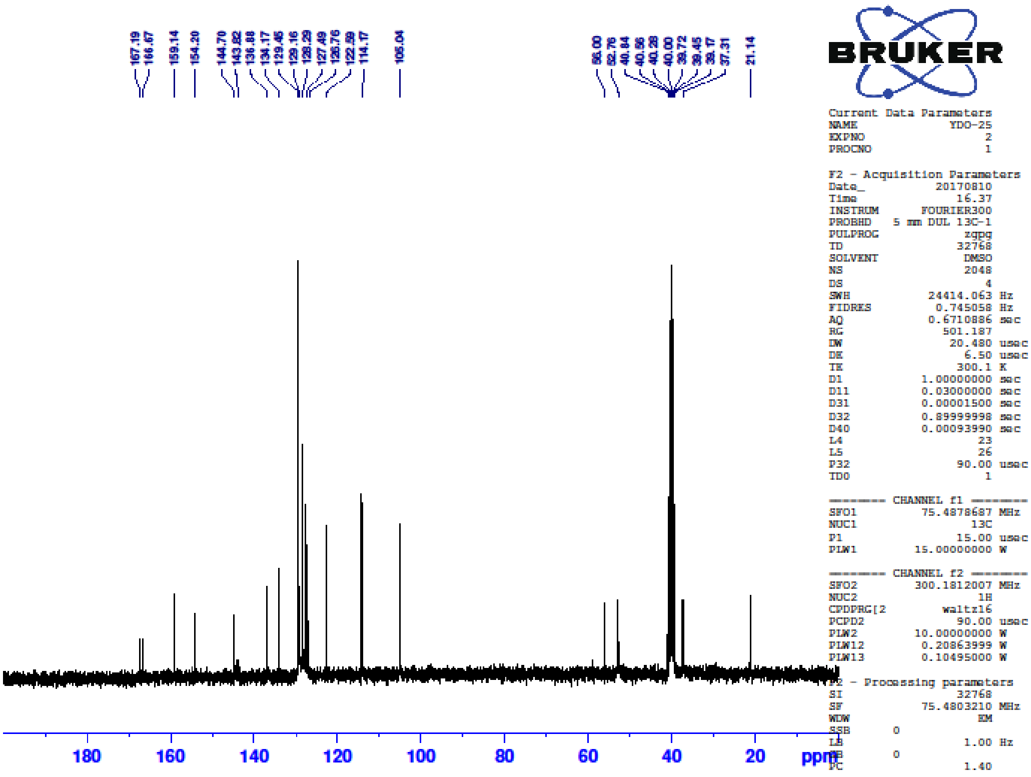

Spectra 80. ^{13}C-NMR spectra of compound **4u**
